# Synthesis, characterisation, and catalytic application of a soluble molecular carrier of sodium hydride activated by a substituted 4-(dimethylamino)pyridine

**DOI:** 10.1038/s42004-024-01184-5

**Published:** 2024-04-27

**Authors:** Peter A. Macdonald, Alan R. Kennedy, Catherine E. Weetman, Stuart D. Robertson, Robert E. Mulvey

**Affiliations:** WestCHEM, Department of Pure & Applied Chemistry, University of Strathclyde, Glasgow, G1 1XL UK

**Keywords:** Organometallic chemistry, Homogeneous catalysis

## Abstract

Recently main group compounds have stepped into the territory of precious transition metal compounds with respect to utility in the homogeneous catalysis of fundamentally important organic transformations. Inspired by the need to promote more sustainability in chemistry because of their greater abundance in nature, this change of direction is surprising since main group metals generally do not possess the same breadth of reactivity as precious transition metals. Here, we introduce the dihydropyridylsodium compound, Na-1,2-*t*Bu-DH(DMAP), and its monomeric variant [Na-1,2-*t*Bu-DH(DMAP)]·Me_6_TREN, and demonstrate their effectiveness in transfer hydrogenation catalysis of the representative alkene 1,1-diphenylethylene to the alkane 1,1-diphenylethane using 1,4-cyclohexadiene as hydrogen source [DMAP = 4-dimethylaminopyridine; Me_6_TREN = tris(*N*,*N*-dimethyl-2-aminoethyl)amine]. Sodium is appealing because of its high abundance in the earth’s crust and oceans, but organosodium compounds have been rarely used in homogeneous catalysis. The success of the dihydropyridylsodium compounds can be attributed to their high solubility and reactivity in organic solvents.

## Abstract (web summary)

Organosodium compounds are appealing sustainable alternatives to precious transition metal compounds, yet they have rarely been used in homogeneous catalysis. Here, the authors introduce a dihydropyridylsodium compound, Na1,2-*t*Bu-DH(DMAP), and its monomeric variant [Na-1,2-*t*BuDH(DMAP)]·Me_6_TREN, and demonstrate their effectiveness in transfer hydrogenation catalysis of a representative alkene, 1,1-diphenylethylene, using 1,4-cyclohexadiene as a hydrogen source.

## Introduction

Throughout the two decades of the 21st century, main group chemistry has been developing in areas previously considered out of bounds for these elements^1^. Sometimes these developments have been described collectively as a renaissance, but they are probably more accurately described as new beginnings. Following his discovery of a germanium compound^2^ able to break the strong covalent bond in H_2_, Power’s vision of main group compounds imitating transition metal chemistry^3^ was surely epiphanic in this regard. Since then, the number of main group compounds reported as catalysts or co-catalysts in reactions usually carried out by transition metal complexes has grown significantly, especially main group metal hydride compounds^4,5^. In another development the previously barren landscape of low valent aluminium (I) chemistry has been transformed into fertile land^6,7^ often realised through alkali metal mediation^8^.

Drilling down the main group chemistry literature, one element that is attracting more recent attention is sodium. Synthetic chemists have rarely considered organosodium compounds as useful chemical reagents, probably because of the success of organolithium reagents that prompted their commercial availability and the greater challenge in handling their perceived more reactive, but significantly less studied heavier sodium congeners. Motivation for the recent upsurge in the study of organosodium chemistry^9^ has been attributed to the World’s ever sharpening focus on sustainability, specifically in this case meaning finding a solution to the rising threat to supplies of lithium (and therefore to organolithium compounds that are ubiquitous in fine chemical manufacture) due to the escalating use of lithium in energy technology^10^. Plentiful in the earth’s crust and oceans, and the sixth most abundant element overall, sodium, about 1500 times more abundant than lithium^11^ is the obvious alternative to make up for any shortfall of lithium in the chemical industry. Therefore, different campaigns are underway to advance the chemistry of sodium organoamides, especially with diisopropylamide (DA), 1,1,3,3-hexamethyldisilazide (HMDS), and 2,2,6,6-tetramethylpiperidide (TMP), the so-called utility amides^12^, which have been so prolifically successful in their lithium form. Highlights include detailed studies of NaDA in synthesis^13^, unravelling solvent-modulated aggregation phenomena of NaHMDS^14^ and their influences on reactivity^15^, and establishing that NaTMP can catalyse perdeuteration of arenes via hydrogen isotope exchange when activated by *N,N,N’,N”,N”*-pentamethyldiethylenetriamine (PMDETA)^16^. Accessed using sodium dispersions and inexpensive arylchlorides, organosodium compounds have also proved effective transmetallating alternatives to organolithium compounds in transition metal catalysed cross-coupling reactions^17^. Remarkably, onward reactivity of intermediate organosodium compounds has even been achieved in reaction mixtures containing protic substances such as water, glycerol, deep eutectic solvents (DESs) or air, all generally nemeses of polar organometallic compounds^18^. A selection of these and other recent advances has been captured in a mini review^19^.

**Fig. 1:**
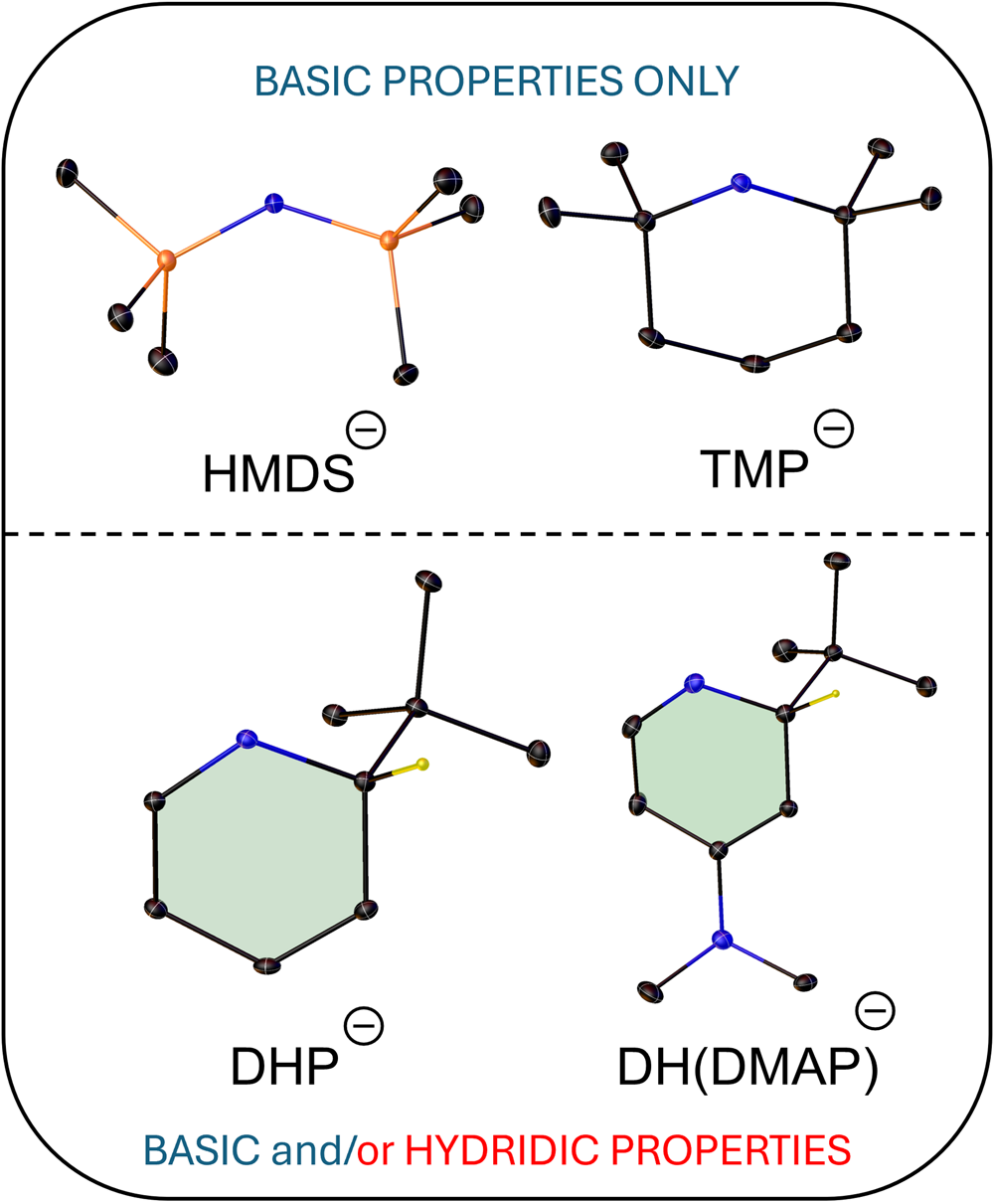
Empirical structures of amides relevant to this catalytic study. Atoms in each molecule are represented by colours as follows: Carbon (black), nitrogen (blue), silicon (orange) hydrogen (yellow). Note the non-planar DHP rings are shaded in grey.

The original research we report herein intersects two important developmental aspects of organosodium chemistry. First, the necessity to expand the number of monomeric compounds within the area^20,21^, since such small molecular compounds can be highly reactive and second, to provide exemplars for new catalysts in fundamental organic transformations^22^. To meet these objectives, we have focused on establishing unique sodium dihydropyridyl compounds. Alkali metal dihydropyridyl compounds, in which the alkali metal and an anionic ligand such as an alkyl have added across the azomethine C-N bond to break the aromaticity of the N-heterocycle are appealing being simple to prepare, and versatile in their reactivity since they can exhibit both protic and (surrogate) hydridic behaviour^23,24^. Herein, we report the synthesis of Na-1,2-*t*Bu-DH(DMAP), **1** (we use DH(DMAP) as a trivial acronym for dihydro 4-dimethylamino pyridine), its solvated monomeric derivative [Na-1,2-*t*Bu-DH(DMAP)]·Me_6_TREN, **1·Me**_**6**_**TREN** and compare their performances with more common sodium reagents in the transfer hydrogenation catalysis of the model alkene 1,1-diphenylethylene using 1,4-cyclohexadiene (see Fig. 1). Unlike saline sodium hydride, which is generally insoluble in organic solvents, a significant limiting factor in its utilisation, **1·Me**_**6**_**TREN** possesses good solubility in both arene and ether solvents and so can be regarded as a soluble carrier of a molecular NaH unit, a beneficial factor exploited here in our catalytic studies.

## Results and discussion

**Fig. 2:**
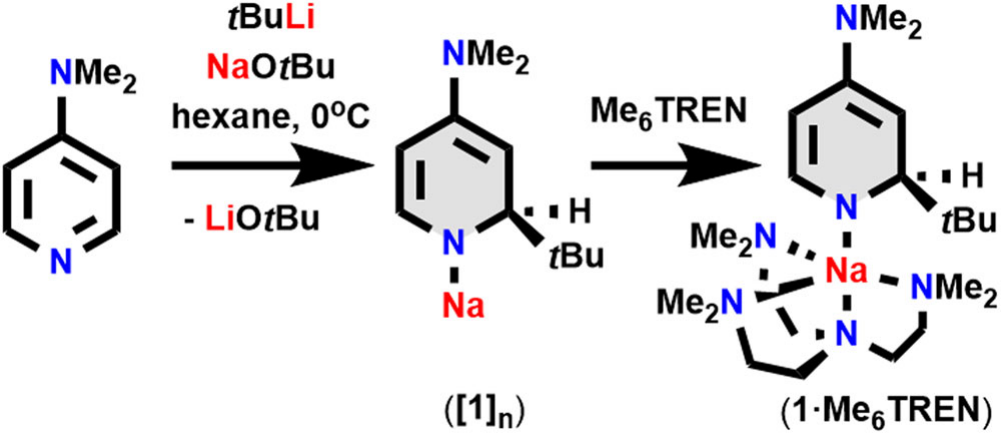
Synthetic protocol to obtain dihydropyridylsodium complexes. Reaction of 4-DMAP with *t*BuLi/NaO*t*Bu in hexane followed by monomerization using Me_6_TREN to yield the desired dihydropyridyl sodium complex.

Since Davidson and Mahon et al. introduced Me_6_TREN to alkali metal chemistry in 2010 and reported a monomeric sodium complex in the aryloxide ArONa·Me_6_TREN (Ar is 2,6-*t*Bu_2_-4-MeC_6_H_2_)^25^ then followed up by us introducing the first Me_6_TREN-complexed alkyl sodium monomers in ArCH_2_Na·Me_6_TREN (Ar is C_6_H_5_ or 3,5-Me_2_C_6_H_3_)^26,27^, we decided to stick to this polyamine as the monomerizing agent in this study. Me_6_TREN is also an effective monomerizing agent for other reactive s-block species^28^. For a potential catalytically active sodium complex we selected a modified dihydropyridyl complex. In previous catalytic transfer hydrogenation studies of imines to amines^24^ we have shown that 2-*t*BuC_5_H_5_NM, M(*t*BuDHP), complexes (M = alkali metal) can be effective pre-catalysts though results using M = sodium were disappointing in comparison to those of the heavier alkali metals. Therefore in this study we have synthesised [Na-1,2-*t*Bu-DH(DMAP)]·Me_6_TREN, in which a NMe_2_ substituent replaces a hydrogen atom at the 4-position of the pyridyl ring, in an attempt to see if this group modified the reactivity. This was indeed prepared by an in situ transmetallation reaction of the lithium 1,2-*t*Bu-DH(DMAP) congener and the bulky alkoxide NaO*t*Bu in hexane solution to yield **1·Me**_**6**_**TREN** (Fig. 2) and isolated in an 80% yield as yellow crystals after recrystallisation from hexane/Me_6_TREN. A direct route using *t*BuNa is not viable due to the instability of alkylsodium compounds as previously noted in M(*t*BuDHP) literature^29^.

Monomer **1·Me**_**6**_**TREN** has been characterised by solution NMR spectroscopic studies and single crystal X-ray diffraction (SCXRD) studies. Diagnostic of its asymmetric 2-substituted dihydro formulation and loss of aromaticity, resonances appear at 7.23, 4.69, 4.02 and 3.76 ppm in its ^1^H NMR spectrum in C_6_D_6_ solution (see Supplementary Fig. 5 for full details). Estimating the molecular weight of **1·Me**_**6**_**TREN** by diffusion ordered spectroscopy (DOSY) NMR studies^30^ in C_6_D_6_ solution strongly suggests that our target of a monomeric sodium complex has been successfully reached. The error in the DOSY data is low at either 4% or -5% assuming the molecule has a dissipated sphere and ellipsoid shape (DSE) or other shape (see the Supporting Information section 2 - Supplementary Figs. 13–16 and Supplementary Tables 2–9). Isolated as a white solid in a 90% yield, **[1]**_**n**_, made in the absence of Me_6_TREN also produced DOSY data consistent with a monomeric species but of formula **1·(d**_**8**_**-THF)**_**4**_, which seems logical from a coordination number perspective given the structure of **1·Me**_**6**_**TREN**.

**Fig. 3:**
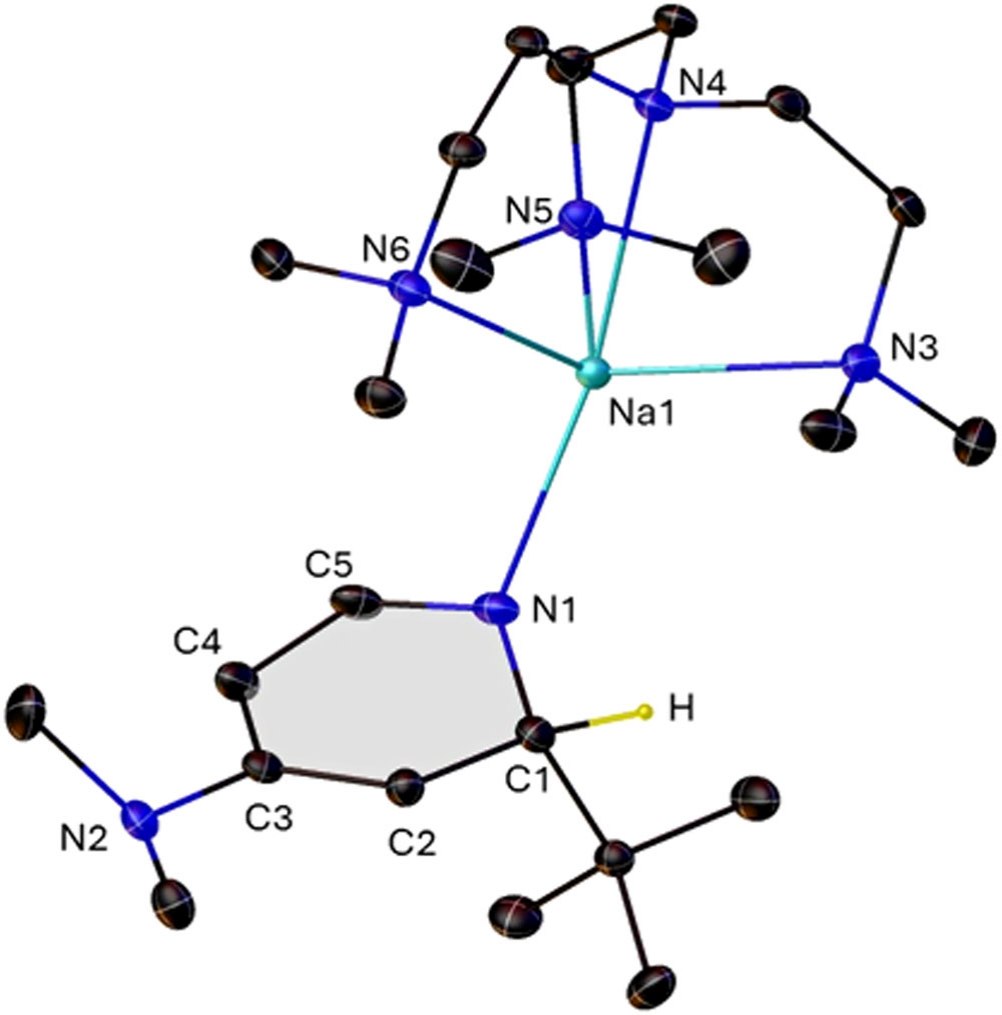
Molecular structure of 1·Me_6_TREN. The molecular structure of **1·Me**_**6**_**TREN** with ellipsoids shown at 50% probability and all hydrogen atoms except that on C1 and minor disordered components of Me_6_TREN ligand removed for clarity. Selected bond lengths and angles: Na1-N1, 2.344(2); Na1-N3, 2.557(2); Na1-N4, 2.564(2); Na1-N5, 2.484(2); Na1-N6, 2.528(2); N1-C1, 1.480(3); C1-C2, 1.513(3); C2-C3, 1.363(3); C3-C4, 1.425(3); C4-C5, 1.382(4); C5-N1, 1.317(4); C3-N2, 1.428(3); C1-N1-C5, 115.5(2); N1-C1-C2, 112.2(2); C1-C2-C3, 122.2(2); C2-C3-C4, 118.3(2); C3-C4-C5, 116.1(2); C4-C5-N1, 129.0(2); C2-C3-N2, 122.1(2); C4-C3-N2, 119.6(2).

While there are lithium examples in the literature^23,31^, complex **1·Me**_**6**_**TREN** represents the first monomeric dihydropyridyl sodium complex to be characterized crystallographically (Fig. 3). The Me_6_TREN ligates sodium via all four Lewis basic nitrogen atoms, consistent with all previously characterized Na/Me_6_TREN complexes^32–34^. The hydrogen atom on C1 could be located and refined, confirming its quaternary sp^3^ nature. Bond lengths around the ring are consistent with loss of aromaticity and confirm the conjugated double bond system running through the C_5_ unit as is typically the case in such DHP anions. Loss of aromaticity is further confirmed by the non-planar nature of the six-membered ring. The Na1-N1-C3 angle of 130.86(8)^o^ is consistent with an anionic ligand which has the negative charge localized at the ring nitrogen rather than a neutral Lewis donating ring which would be expected to bind linearly. The description of the DH(DMAP) ligand as a secondary amide is further supported by the Na1-N1 bond distance of 2.344(2) Å, which is intermediate between that of monomeric (HMDS)Na·PMDETA [2.285(1) Å; 4-coordinate Na]^35^ and Me_6_TREN solvated secondary amides generated via methyl deprotonation of picolines followed by relocalization of the negative charge on to the ring nitrogen [2.391(2) Å, 5-coordinate Na]^36,37^. Loss of aromaticity is further manifested through loss of planarity at the exocyclic NMe_2_ group. Neutral DMAP has a planar NMe_2_ on account of resonance stabilization experienced through lone pair donation into the ring and is exemplified by the average sum of bond angles in a recently reported [Li·4DMAP]^+^ moiety of 359.55^o^^38^, in **1·Me**_**6**_**TREN** this value is 342.97^o^, deviating considerably from trigonal planarity. The crystal structure of unsolvated **1** could not be determined due to its insolubility in non-Lewis-base donor solvents such as hexane. It is likely that this complex adopts a polymeric arrangement as is commonplace in unsolvated sodium amide chemistry. An infinite network made up of ionic Na-N bonds and non-covalent Na-π-DHP interactions, similar to those seen between alkali-metals and an Al-bound DHP ligand^39,40^, is likely.

**Fig. 4:**
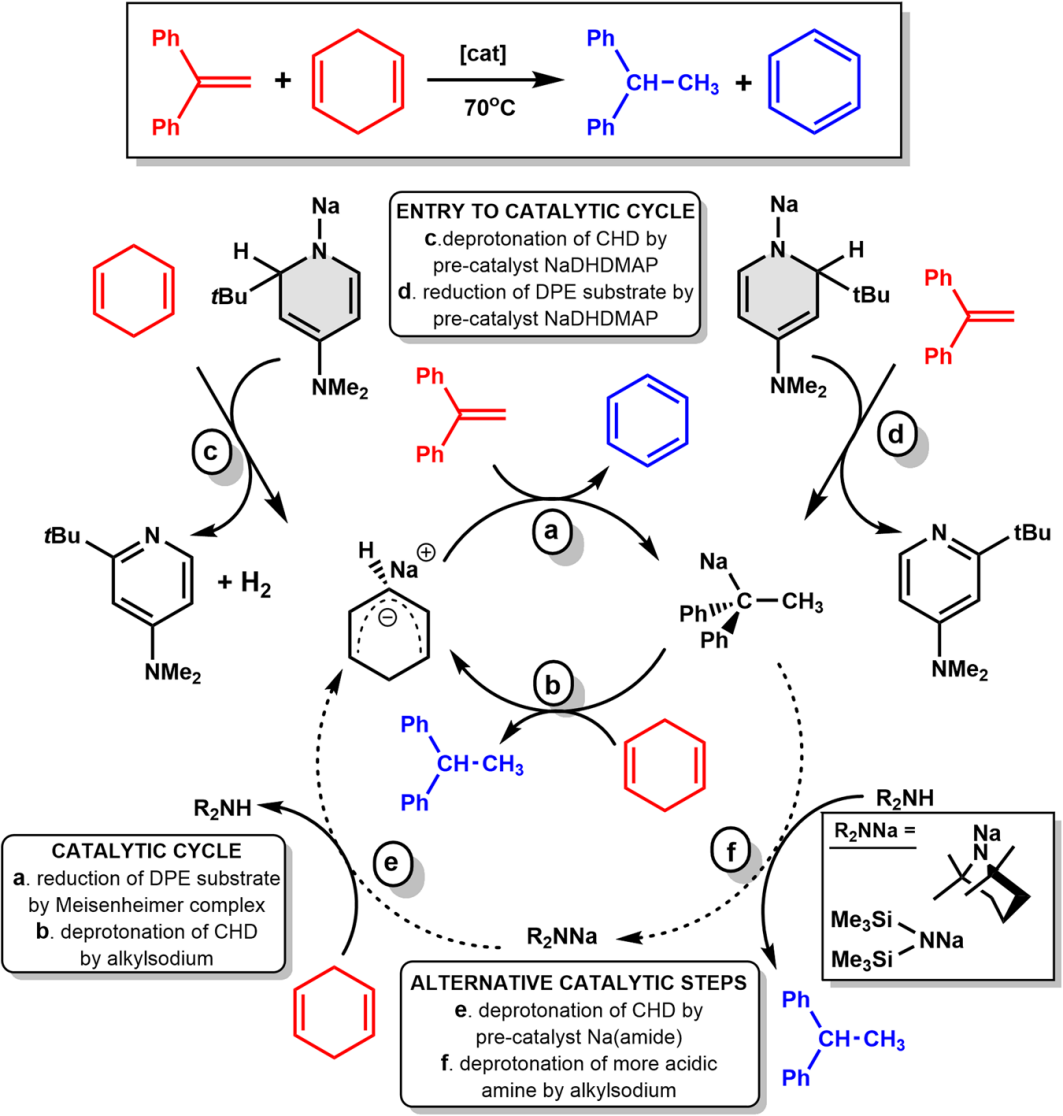
Proposed catalytic mechanism for transfer hydrogenation of 1,1-diphenylethylene with 1,4-cyclohexadiene to yield 1,1-diphenylethane and benzene. All reactants are shown in red and final products are shown in blue.

To test whether monomeric sodium dihydropyridyl **1·Me**_**6**_**TREN** showed any catalytic activity we chose the conversion of 1,1-diphenylethylene (DPE) to 1,1-diphenylethane as a representative reduction reaction (see inset reaction in Fig. 4). Previous studies^41–43^ have established that 1,4-cyclohexadiene (CHD) is a convenient, easily handled liquid transfer hydrogenation reagent so it was employed as the hydrogen source. Alkali-metal-mediated reactions are often solvent dependent with some of the most distinct behaviours found between low-polarity arene and polar ether solvents, therefore C_6_D_6_ and d_8_-THF respectively were investigated as the medium in this study with the temperature fixed at 70 °C and catalyst loading at 10 mol%. For **1**, in the absence of Me_6_TREN, the reaction took 24 hours in C_6_D_6_ to reach quantitative (99%) conversion as measured by consumption of the alkene substrate 1,1-diphenylethylene with virtually quantitative yield of the product,1,1-diphenylethane (Table 1, entry 1). However, in d_8_-THF, 99% conversion was accomplished in just 0.5 hours, with essentially no reduction in product yield (entry 3). Solubility is not an issue as **1** is fully soluble at 70 °C in both solvents though its solubility drops in C_6_D_6_ at 25 °C. Analogous reactions with monomeric **1·Me**_**6**_**TREN** reached full conversion in both solvents in less than one hour though there were small reductions of 6% and 10% in product yields (93% and 89%) in C_6_D_6_ and THF-d_8_ respectively (entries 2 and 4). These results strongly hint that the key factor in catalytic performance is the size of the molecular structure of the catalyst in solution with the aforementioned DOSY studies implicating monomeric structures for both **1·Me**_**6**_**TREN** in C_6_D_6_ (consistent with its solid-state structure) and **1** in d_8_-THF which is likely to be **1·(THF-d**_**8**_**)**_**4**_. These values are far superior to their lithium counterparts demonstrating the importance of sodium in this catalysis. In benzene solution, complete conversion is not achieved after 24 h, with yields of only 24 and 31% witnessed in the absence and presence of Me_6_TREN respectively (entries 5 and 6). In THF solution, reactions reach 99% conversion after 6 hours (no Me_6_TREN, entry 7) and 3 hours (with Me_6_TREN, entry 8) but with inferior yields of 68 and 44%.

**Table 1:** Comparison of data for sodium-catalysed conversion of 1,1-diphenylethylene to 1,1-diphenylethane

Entry	Catalyst	Equivalents of Me_6_TREN	Solvent	T [^o^C]	Time [h]	Conversion [%]	Yield [%]
1	**NaDH(DMAP)**	**0**	**C**_**6**_**D**_**6**_	**70**	**24**	**99**	**99**
2	**NaDH(DMAP)**	**1**	**C**_**6**_**D**_**6**_	**70**	**0.5**	**99**	**93**
3	**NaDH(DMAP)**	**0**	**THF-d**_**8**_	**70**	**0.5**	**99**	**98**
4	**NaDH(DMAP)**	**1**	**THF-d**_**8**_	**70**	**1**	**99**	**89**
5	**LiDH(DMAP)**	**0**	**C**_**6**_**D**_**6**_	**70**	**24**	**30**	**24**
6	**LiDH(DMAP)**	**1**	**C**_**6**_**D**_**6**_	**70**	**24**	**80**	**31**
7	**LiDH(DMAP)**	**0**	**THF-d**_**8**_	**70**	**6**	**99**	**68**
8	**LiDH(DMAP)**	**1**	**THF-d**_**8**_	**70**	**3**	**99**	**44**
9	**NaH**	**1**	**C**_**6**_**D**_**6**_	**70**	**24**	**0**	**0**
10	**NaH**	**1**	**THF-d**_**8**_	**70**	**24**	**0**	**0**
11	**NaHMDS**	**1**	**C**_**6**_**D**_**6**_	**70**	**5**	**95**	**79**
12	**NaHMDS**	**1**	**THF-d**_**8**_	**70**	**24**	**85**	**80**
13	**NaTMP**	**1**	**C**_**6**_**D**_**6**_	**70**	**2.5**	**99**	**95**
14	**NaTMP**	**1**	**THF-d**_**8**_	**70**	**24**	**99**	**57**
15	***n*BuNa**	**1**	**C**_**6**_**D**_**6**_	**70**	**3**	**99**	**96**
16	***n*BuNa**	**1**	**THF-d**_**8**_	**70**	**3**	**99**	**50**
17	**NaDHP**	**0**	**C**_**6**_**D**_**6**_	**70**	**24**	**50**	**29**
18	**NaDHP**	**1**	**C**_**6**_**D**_**6**_	**70**	**3**	**99**	**99**
19	**NaDHP**	**0**	**THF-d**_**8**_	**70**	**3**	**99**	**80**
20	**NaDHP**	**1**	**THF-d**_**8**_	**70**	**3**	**99**	**81**

Reactions all carried out with 10 mol% catalyst loading using 0.3 mmole scale reaction in 0.5 mL using 1.5 equivalents of 1,4-CHD. Conversions were calculated by depletion of 1,1-diphenylethylene (integration versus SiMe_4_ standard) while yields were calculated on generation of 1,1-diphenylethane (also integration versus SiMe_4_ standard).

The next part of our study compared these catalytic performances with those of other related sodium compounds under the same conditions. These comprised NaDHP, [Na-1,2-*t*Bu-DHP], where the 4-substituent has switched from a dimethylamino group in **1** to a hydrogen atom, the aforementioned utility amides NaHMDS and NaTMP, an alkylsodium reagent (*n*BuNa) and the common bench reagent NaH. The most predictable outcome was the failure of the salt NaH (entries 9 and 10) to generate any product at all due to its insolubility in both organic solvents even in the additional presence of a molar equivalent of Me_6_TREN. We note that Chiba recently reduced 1,1-DPE using excess of a NaH/NaI mixture in 87% yield although this required a solvothermal process at 100 ^o^C for one day^44^. Sterically more demanding NaTMP is known to be a stronger more reactive Brønsted base than NaHMDS^12^, which may give it an advantage in any initial deprotonation of cyclohexadiene (see mechanism discussion, *vide infra*) but the comparative data in Table 1, though in the expected order of reactivity, are not significantly different (see Supplementary Information section 3 - Supplementary Figs. 17–36, for all related spectra). In the presence of a molar equivalent of Me_6_TREN each amide performs well in C_6_D_6_ with NaTMP having a slight edge in both reaction time and product yield (by 2.5 hours and 16%, respectively, entries 11 and 13), though significantly these reactions are slower than those with **1·Me**_**6**_**TREN** which are complete within 0.5 hours (entry 2). The performances of NaHMDS and NaTMP fall off sharply in THF-d_8_ (entries 12 and 14), taking 24 hours to reach completion (in the case of NaHMDS to only 85%) and a degree of decomposition is apparent with NaTMP reflected by a drop in product yield to 57%. Degradation of reaction mixtures of NaTMP in THF have previously been noted^45^ and stoichiometric reaction of NaTMP with DPE shows deprotonative processes occur. NMR spectra show a number of unidentified resonances although some appear to be consistent with formation of 1,1,2-triphenylethane^46^ and display no evidence for dimerization products as witnessed previously by Harder^47^. These substantially longer reaction times suggest that the mechanisms in bulk THF-d_8_ medium for both NaTMP and NaHMDS with added Me_6_TREN differ substantially from those in C_6_D_6_. No such large solvent-dependent distinction was witnessed with **1·Me**_**6**_**TREN**, implying that the mechanism/s of its reactions are likely to be similar, though the possibility of different mechanisms due to different aggregations/stabilities of these amides in the solvents employed cannot be ruled out. The strong alkyl base *n*BuNa was also tested, showing similar results to NaTMP. Specifically, conversion was complete within 3 h, with the yield of the desired product dropping from 96% in C_6_D_6_ to 50% in THF (entries 15 and 16). Moving to the results for NaDHP, where the Me_2_N group has been removed from the dihydropyridyl ring, the trends observed seem more akin to those of NaDH(DMAP) than to those of the utility amides. Entry 17 bears some resemblance to that of entry 1 since in the absence of any Lewis base donor solvent catalytic reaction time is extended considerably in C_6_D_6_ though the DH(DMAP) pre-catalyst is superior to its less substituted rival in both conversion (99% vs. 50%) and product yield (99% vs. 29%). On addition of monomerising agent Me_6_TREN, NaDHP improved by orders of magnitude with 99% conversion achieved in only 3 h in both C_6_D_6_ and THF-d_8_ with product yields high (99% and 81%, entries 18 and 20), though as seen with NaDH(DMAP) some instability of the reaction solution is seen in bulk THF-d_8_. NaDHP also performs well in neat THF-d_8_ without any Me_6_TREN presence, exactly matching its performance in that donor solvent when an equivalent of Me_6_TREN is present (entry 19). The data collected for NaDHP in entries 18-20, lie in the same ballpark as those for NaDH(DMAP) in entries 2–4, though in every case NaDH(DMAP) is modestly superior with reactions completed in about 1 hour compared to 3 hours in 93-99% compared to 80-99% product yields. A plausible empirical conclusion at this stage could be that NaDHP and NaDH(DMAP) could be operating in a similar way in these catalytic processes and that the relatively small differences between them could be due to the electronic and/or steric influences of the Me_2_N substituent attached to the ring in the latter compound. This is corroborated by preliminary theoretical investigations, wherein comparison of the DHP and DH(DMAP) charge densities reveal minor subtle differences between the two ring systems. CHELPG charges highlight the effect of including an electron donating group, as a slight increase in the negative charge is seen on the nitrogen atom (NaDHP -0.986 vs NaDHDMAP -1.068) in line with the expected increase in nucleophilic character (see Supplementary Information section 5—Supplementary Figs. 40–41 and Supplementary Tables 10–13).

The development of catalysts and pre-catalysts for environmentally benign organic transformations including alkene to alkane transformation is a highly active area of research. Most literature catalysts to date feature transition-metal complexes^48–51^, but increasing emphasis is being placed on examples based on main-group elements for the reasons alluded to in the introduction. The specific reduction reaction of 1,1-diphenylethylene to 1,1-diphenylethane investigated here has been studied with several such main group element catalysts, since it is least prone to unwanted side reactions so is a good model alkene^42,47,52–56^. Most relevant to our present study are those based on sodium’s nearest heavier congener potassium^57^. Guan found that KH (10 mol% loading) afforded less than 5% product when using direct hydrogenation via H_2_ at 6 bar in C_6_D_6_ for 6 h at 60 ^o^C, but this improved to a quantitative yield on mixing KH with KHMDS or M(HMDS)_2_ where M = Mg, Ca, or Zn under the same conditions. We also used a bimetallic approach via the defined compound KMg(HMDS)_3_ (10 mol%) achieving quantitative yield in 1.5 hours at 75 ^o^C using transfer hydrogenation from 1,4-cyclohexadiene^8^.

We are therefore able to propose a catalytic cycle, taking the above cited previous literature and our observations into account (Fig. 4). The principal catalytic cycle likely involves reduction of the DPE substrate by the sodium pseudo-Meisenheimer intermediate (step a) which generates 1,1-diphenylethylsodium with concomitant release of benzene. This substituted alkyl sodium can then operate as a Brønsted base in its own right, deprotonating CHD to reform the sodium pseudo-Meisenheimer complex and release the 1,1-diphenylethane product (step b). It is plausible that the variable results discussed above are a consequence of distinct entry points available to the sodium pre-catalysts. Dihydropyridyl complexes have access to two points of entry as they can act as conventional sodium amide bases, deprotonating CHD to generate the intermediate Na pseudo-Meisenheimer complex (path c) or alternatively as a molecular sodium-hydride induced reducing agent for the generation of the alkyl sodium intermediate (path d). Both paths c and d generate rearomatized 2-*t*butyl-4-dimethylaminopyridine, as witnessed in their ^1^H NMR spectra (see Supplementary Information section 4 - Supplementary Figs. 37-39). The possibility of **1** acting as a reducing agent is supported by its stoichiometric reaction with the unsaturated substrate, which after 2 hours at 70^o^C in C_6_D_6_ solvent results in essentially complete conversion to 1,1-diphenylethylsodium, as evidenced by generation of a singlet representing the methyl group, at 2.34 ppm. We also probed pathway c stoichiometrically and duly observed evolution of H_2_ at 4.55 ppm in the ^1^H NMR spectrum after one hour at 70 ^o^C in THF-d_8_ alongside rearomatization of the pyridine, confirming the viability of this alternative entry point. In contrast, the sodium utility amides can act as bases only, accessing the catalytic cycle by deprotonating CHD to give Na pseudo-Meisenheimer complex with concomitant formation of secondary amine (step e). This then introduces the possibility of this non-volatile by-product being preferentially deprotonated at a later stage by the ethylsodium (step f), forming the reduced organic product whilst reforming the sodium utility amide and introducing an alternative catalytic cycle with an additional step.

## Conclusions

In this research we have synthesised a target sodium amide complex, but a specialised type in that it is a DMAP-substituted dihydropyridyl complexed by the polydentate polyamine Me_6_TREN. As planned for, the reported complex here is a monomer in the solid-state and from DOSY studies appears to retain this state in C_6_D_6_ solution. The specialness of this sodium amide lies in its twofold reactivity as our observations show that it can act both as a conventional metal amide in performing deprotonating reactions and a molecular hydride source, whereas conventional metal amides only possess the former reactivity. This reactivity of **1·Me**_**6**_**TREN** has proved useful in the representative reduction reaction of the alkene Ph_2_C = CH_2_ to the alkane Ph_2_(H)C-CH_3_, in which it has outperformed the common utility amides NaHMDS and NaTMP as well as the insoluble, inert sodium hydride which showed no catalytic reactivity at all (although stoichiometric reactivity is possible under extreme conditions). These results underline that with proper development compounds of earth abundant sodium could begin to join the elite compounds of scarce transition metals with regard to their usefulness in homogeneous catalysis of fundamentally important organic transformations. Future work will examine the scope of this reaction with a library of substrates, probe the mechanisms of the catalyses by theoretical calculations, and extend the use of this sodium amide monomer (and other related dihydropyridyl monomers yet to be synthesised) to other industrially important organic reactions.

## Methods

For general experimental procedures see Supplementary Information section 1 - Supplementary Methods, Supplementary Figs. 1–12 and Supplementary Table 1.

### Synthesis of Na-1,2-*t*Bu-DH(DMAP) (1)

DMAP (0.366 g, 3 mmol) was added to a Schlenk flask along with hexane (15 ml) and NaO*t*Bu (0.288 g, 3 mmol). The solution mixture was cooled to 0 ^o^C using an ice bath and then *t*BuLi (1.7 M in pentane, 1.76 ml, 3 mmol) was added dropwise via syringe. The resultant cloudy yellow solution was left to stir for 4 hours producing an off-white suspension. The solvent was then removed via cannula filtration and the white solid was dried under reduced pressure. The compound was then transferred into the glove box, weighed and stored at -20 ^o^C in the freezer as a white powder material (0.55 g, 90% yield).

^1^H NMR [400.03 MHz, 300 K, THF(d_8_)]: δ 0.82 (s, 9H, *t*Bu), 2.54 (s, 6H, NMe_2_), 3.10 (dd, 1H, C1-**H**), 3.45 (d, 1H, C2-**H**), 4.06 (dd, 1H, C4-**H**), 6.80 ppm (d, 1H, C5-**H**); ^13^C {^1^H} NMR [100.60 MHz, 300 K, THF(d_8_)]: δ 152.00 (**C5**-H), 80.17 (**C4**-H), 69.59 (**C1**-H) 68.78 (**C2**-H), 39.61 (NMe_2_), 39.12 (quaternary [DH(DMAP)]), 24.70 ppm (*t*Bu).

### Synthesis of [Na-1,2-*t*Bu-DH(DMAP)]·Me_6_TREN (1·Me_6_TREN)

Na-1,2-*t*Bu-DH(DMAP) (1 mmol, 0.202 g) was added to a vial of hexane (5 ml) in the glovebox. Me_6_TREN (1 mmol, 0.26 ml) was subsequently added, and the suspension was left to stir for 5 minutes. The resultant yellow solution was placed into the freezer at −20 ^o^C. After 24 hours transparent large block crystals had formed which were isolated and stored in the glovebox freezer (0.34 g, 80% crystalline yield).

^1^H NMR [400.03 MHz, 300 K, C_6_D_6_]: δ 1.45 (s, 9H, *t*Bu), 2.07 (s, 30H, Me_6_TREN), 2.95 (s, 6H, NMe_2_), 3.76 (dd, 1H, C1-**H**), 4.02 (d, 1H, C2-**H**), 4.70 (dd, 1H, C4-**H**), 7.23 ppm (d, 1H, C5-**H**); ^13^C {^1^H} NMR [100.60 MHz, 300 K, C_6_D_6_]: δ 153.00 (**C5**-H), 79.85 (**C4**-H), 71.79 (**C1**-H), 70.60 (**C2**-H), 56.60 (CH_2_ Me_6_TREN), 50.88 (CH_2_ Me_6_TREN), 44.58 (CH_3_ Me_6_TREN), 42.68 (NMe_2_), 41.05 (quaternary [DH(DMAP)]), 25.40 ppm (*t*Bu).

### Supplementary information

Supplementary Information

Description of Additional Supplementary Files

Supplementary Data 1

## Data Availability

The datasets generated during and/or analysed during the current study are available in the PURE repository, 10.15129/3fb4efe4-1841-4fa0-a326-cd0e3cbc866b. The X-ray crystallographic coordinates for the structure reported in this study have been deposited at the Cambridge Crystallographic Data Centre (CCDC), under deposition number 2327713. These data can be obtained free of charge from The Cambridge Crystallographic Data Centre via www.ccdc.cam.ac.uk/data_request/cif. General experimental procedures, DOSY NMR spectroscopic details, NMR spectra from catalytic and stoichiometric reactions and computational details are provided in Supplementary Information and cif file for complex **1·Me**_**6**_**TREN** is available as Supplementary Data file 1.

## References

[1] Gentner TX, Mulvey RE (2021). Alkali-metal mediation: Diversity of applications in main-group organometallic chemistry. Angew. Chem. Int. Ed..

[2] Spikes GH, Fettinger JC, Power PP (2005). Facile activation of dihydrogen by an unsaturated heavier main group compound. J. Am. Chem. Soc..

[3] Power PP (2010). Main-group elements as transition metals. Nature.

[4] Weetman C, Inoue S (2018). The road travelled: After main-group elements as transition metals. ChemCatChem.

[5] Roy MMD (2021). Molecular main group metal hydrides. Chem. Rev..

[6] Coles MP, Evans MJ (2023). The emerging chemistry of the aluminyl anion. Chem. Commun..

[7] Hobson K, Carmalt CJ, Bakewell C (2020). Recent advances in low oxidation state aluminium chemistry. Chem. Sci..

[8] Gentner TX, Kennedy AR, Hevia E, Mulvey RE (2021). Alkali Metal (Li, Na, K, Rb, Cs) mediation in magnesium hexamethyldisilazide [Mg(HMDS)_2_] catalysed transfer hydrogenation of alkenes. ChemCatChem.

[9] Wong HNC (2019). Is sodium finally coming of age?. Nat. Catal..

[10] Tabelin CB (2021). Towards a low-carbon society: A review of lithium resource availability, challenges and innovations in mining, extraction and recycling, and future perspectives. Miner. Eng..

[11] https://www.rsc.org/periodic-table/element/11/sodium and https://www.rsc.org/periodic-table/element/3/lithium.

[12] Mulvey RE, Robertson SD (2013). Synthetically Important Alkali-Metal Utility Amides: Lithium, Sodium, and Potassium Hexamethyldisilazides, Diisopropylamides and Tetramethylpiperidides. Angew. Chem. Int. Ed..

[13] Woltornist RA (2020). Structure, reactivity, and synthetic applications of sodium diisopropylamide. Synthesis.

[14] Woltornist RA, Collum DB (2021). Aggregation and solvation of sodium hexamethyldisilazide: Across the solvent spectrum. J. Org. Chem..

[15] You Q, Collum DB (2023). Carbon-nitrogen bond formation using sodium hexamethyldisilazide: Solvent-dependent reactivities and mechanisms. J. Am. Chem. Soc..

[16] Tortajada A, Hevia E (2022). Perdeuteration of arenes via hydrogen isotope exchange catalyzed by the superbasic sodium amide donor species NaTMP·PMDETA. J. Am. Chem. Soc..

[17] Asako S, Nakajima H, Takai K (2019). Organosodium compounds for catalytic cross-coupling. Nat. Catal..

[18] Dilauro G (2023). Introducing water and deep eutectic solvents in organosodium chemistry: Chemoselective nucleophilic functionalizations in air. Angew. Chem. Int. Ed..

[19] Anderson DE, Tortajada A, Hevia E (2024). New frontiers in organosodium chemistry as sustainable alternatives to organolithium reagents. Angew. Chem. Int. Ed..

[20] Anderson DE, Tortajada A, Hevia E (2023). Highly reactive hydrocarbon soluble alkylsodium reagents for benzylic aroylation of toluenes using Weinreb amides. Angew. Chem. Int. Ed..

[21] Davison N (2023). Li vs Na: Divergent reaction patterns between organolithium and organosodium complexes and ligand-catalyzed ketone/aldehyde methylenation. J. Am. Chem. Soc..

[22] Whitelaw MT (2022). Catalytic hydrophosphination of alkynes using structurally diverse sodium diphenylphosphide donor complexes. Cell Rep. Phys. Sci..

[23] Robertson SD, Kennedy AR, Liggat JJ, Mulvey RE (2015). Facile synthesis of a genuinely alkane-soluble but isolable lithium hydride transfer reagent. Chem. Commun..

[24] Macdonald PA (2023). Alkali metal dihydropyridines in transfer hydrogenation catalysis of imines: Amide basicity versus hydride surrogacy. Angew. Chem. Int. Ed..

[25] Cousins DM, Davidson MG, Frankis CJ, Garcia-Vivo D, Mahon MF (2010). Tris(2-dimethylaminoethyl)amine: a simple new tripodal polyamine ligand for Group 1 metals. Dalton Trans..

[26] Davidson MG, Garcia-Vivo D, Kennedy AR, Mulvey RE, Robertson SD (2011). Exploiting σ/π coordination isomerism to prepare homologous organoalkali metal (Li, Na, K) monomers with identical ligand sets. Chem. Eur. J..

[27] Armstrong DR (2013). Monomerizing alkali-metal 3,5-dimethylbenzyl salts with Tris(*N*,*N*-dimethyl-2-aminoethyl)amine (Me_6_TREN): Structural and bonding implications. Inorg. Chem..

[28] Banerjee S, Ankur, Andrews A, Venugopal A (2018). A disguised hydride in a butylmagnesium cation. Chem. Commun..

[29] Orr SA (2016). Accessible heavier s-block dihydropyridines: structural elucidation and reactivity of isolable molecular hydride sources. Dalton Trans..

[30] Bachmann S, Neufeld R, Dzemski M, Stalke D (2016). New external calibration curves (ECCs) for the estimation of molecular weights in various common NMR solvents. Chem. Eur. J..

[31] Armstrong DR (2015). Developing lithium chemistry of 1,2-dihydropyridines: From kinetic intermediates to isolable characterized compounds. Chem. Eur. J..

[32] Mukherjee D, Osseili H, Spaniol TP, Okuda J (2016). Alkali metal hydridotriphenylborates [(L)M][HBPh_3_] (M = Li, Na, K): Chemoselective catalysts for carbonyl and CO_2_ hydroboration. J. Am. Chem. Soc..

[33] Kennedy AR, McLellan R, McNeil GJ, Mulvey RE, Robertson SD (2016). Tetraamine Me_6_TREN induced monomerization of alkali metalborohydrides and aluminohydrides. Polyhedron.

[34] Barker J, Davison N, Waddell PG, Lu E (2023). Monomeric lithium and sodium silylbenzyl complexes: syntheses, structures and C=O bond olefination. Chem. Commun..

[35] Borys, A. M. & Hevia, E. Beyond Ni{N(SiMe_3_)_2_}_2_: Synthesis of a Stable Solvated Sodium Tris-Amido Nickelate. *Organometallics* **40**, 442–447 (2021).

[36] Kennedy AR, Mulvey RE, Urquhart RI, Robertson SD (2014). Lithium, sodium and potassium picolyl complexes: Syntheses, structures and bonding. Dalton Trans..

[37] Rae A (2022). Sigma/pi bonding preferences of solvated alkali-metal cations to ditopic arylmethyl anions. Chem. Eur. J..

[38] Reckziegel A, Battistella B, Schmidt A, Werncke CG (2022). Intricate road to linear anionic nickel(I) hexamethyldisilazanide [Ni(N(SiMe_3_)_2_)_2_]^-^. Inorg. Chem..

[39] Banerjee, S. et al. Hydrocarbon Soluble Alkali-Metal-Aluminium Hydride Surrog[ATES]. *Chem. Eur. J.* **28**, e202201085 (2022). 10.1002/chem.202201085 PMC9804340 35811447

[40] Docherty SP (2022). Incorporating trimethylamluminium into the structures of alkali-metal (Li, Na, K, Cs) dihydropyridines. Z. Anorg. Allg. Chem..

[41] Knoevenagel E, Bergdolt B (1903). Ueber das Verhalten des D_2.5_-Dihydroterephtalsäuredimethylesters bei höheren Temperaturen und in Gegenwart von Palladiummohr. Ber. Dtsch. Chem. Ges..

[42] Bauer H (2018). Simple alkaline-earth metal catalysts for effective alkene hydrogenation. Angew. Chem. Int. Ed..

[43] Taleb B (2023). Exploring hydrogen sources in catalytic transfer hydrogenation: A review of unsaturated compound reduction. Molecules.

[44] Chaisan N, Tan EYK, Chiba S (2024). Hydroalkylation of 1,1-diarylalkenes mediated by magnesium hydride in ethereal solvents. Helv. Chim. Acta.

[45] Armstrong DR, Garcia-Alvarez P, Kennedy AR, Mulvey RE, Robertson SD (2011). Molecular structures of thf-solvated alkali-metal 2,2,6,6-tetramethylpiperidides finally revealed: X-ray crystallographic, DFT, and NMR (including DOSY) spectroscopic studies. Chem. Eur. J..

[46] Gieshoff TN, Chakraborty U, Villa M, Jacobi von Wangelin A (2017). Alkene hydrogenations by soluble iron nanocluster catalysts. Angew. Chem. Int. Ed..

[47] Spielmann J, Buch F, Harder S (2008). Early main-group metal catalysts for the hydrogenation of alkenes with H_2_. Angew. Chem. Int. Ed..

[48] Chirik PJ (2015). Iron- and cobalt-catalyzed alkene hydrogenation: Catalysis with both redox-active and strong field ligands. Acc. Chem. Res..

[49] Santana CG, Krische MJ (2021). From hydrogenation to transfer hydrogenation to hydrogen auto-transfer in enantioselective metal-catalyzed carbonyl reductive coupling: past, present and future. ACS Catal..

[50] Wen J, Wang F, Zhang X (2021). Asymmetric hydrogenation catalyzed by first-row transition metal complexes. Chem. Soc. Rev..

[51] Baidilov D, Hayrapetyan D, Khalimon AY (2021). Recent advances in homogeneous base-metal-catalyzed transfer hydrogenation reactions. Tetrahedron.

[52] Schuhknecht D, Lhotzky C, Spaniol TP, Maron L, Okuda J (2017). Calcium hydride cation [CaH]^+^ Stabilized by an NNNN-type macrocyclic ligand: A selective catalyst for olefin hydrogenation. Angew. Chem. Int. Ed..

[53] Shi X (2019). Super-bulky penta-arylcyclopentadienyl ligands: Isolation of the full range of half-sandwich heavy alkaline-earth metal hydrides. Angew. Chem. Int. Ed..

[54] Shi X, Hou C, Zhao L, Deng P, Cheng J (2020). Mononuclear calcium complex as effective catalyst for alkenes hydrogenation. Chem. Commun..

[55] Martin J (2020). Highly active superbulky alkaline earth metal amide catalysts for hydrogenation of challenging alkenes and aromatic rings. Angew. Chem. Int. Ed..

[56] Shi X, Cheng J (2019). Reversible addition and hydrogenation of 1,1-diphenylethylene with a barium complex. Dalton Trans..

[57] Zhang X-Y, Du H-Z, Zhai D-D, Guan B-T (2020). Combined KH/alkaline-earth metal amide catalysts for hydrogenation of alkenes. Org. Chem. Front..

