# Supplementary material for: Synthesis, characterisation, and catalytic application of a soluble molecular carrier of sodium hydride activated by a substituted 4-(dimethylamino)pyridine
Source: Commun Chem. 2024 Apr 27;7:94. doi: 10.1038/s42004-024-01184-5 (PMC11055874; doi:10.1038/s42004-024-01184-5)
Supplement: Supplementary file 1 — Supplementary Information [file 42004_2024_1184_MOESM1_ESM.pdf]

Synthesis, characterisation, and catalytic application of a soluble molecular carrier of sodium hydride activated by a substituted 4-(dimethylamino)pyridine

Peter A. Macdonald, Alan R. Kennedy, Catherine E. Weetman, Stuart D. Robertson and Robert E. Mulvey

SUPPLEMENTARY INFORMATION

Contents

1. Supplementary Methods: General experimental procedures 2
2. DOSY (Diffusion Ordered Spectroscopy) studies 10
   2.1 Na-1,2-*t*Bu-DH(DMAP) 10
   2.2 Na-1,2-*t*Bu-DH(DMAP)·Me_6TREN (1·Me_6TREN) 14
3. Transfer hydrogenation experiments 17
4. Stoichiometric experiments 37
5. Computational Details 40
   5.1 Structural Parameters 40
   5.2 Frontier Orbitals 42
   5.3 Charge Distributions 42
   5.4 Cartesian Coordinates: 43
6. Supplementary References 48

1. Supplementary Methods: General experimental procedures

All synthetic procedures were performed under a dry nitrogen (N_2) atmosphere using standard Schlenk techniques or in a glove box under a recirculating argon (Ar) atmosphere. Prior to use, glassware was dried at 150 ^oC under vacuum and solvents were dried, distilled and degassed using standard methods.[1] Benzene (C_6D_6), and THF-d_8 were dried over molecular sieves (4 Å) and stored in the glove box. n-Hexane, toluene and tetrahydrofuran (THF) were dried in a Solvent Purification System (Innovative Technology, PS-Micro), degassed, and stored under an inert atmosphere over activated 4 Å molecular sieves. Benzene and pyridine were dried over CaH_2, distilled under a N_2 atmosphere, and stored over activated 4 Å molecular sieves prior to use. *Tert*-Butyllithium (*t*BuLi), 4-dimethylaminopyridine (DMAP), 1,1-diphenylethylene (DPE), sodium *tert*-butoxide (NaO*t*Bu), sodium hydride (NaH) and Tris[2-(dimethylamino)ethyl]amine (Me_6TREN) were all purchased from commercial sources. Sodium HMDS, $Na[N(SiMe_3)_2]$, was synthesised following a literature procedure and stored at room temperature in the glove box as a white solid.[2] NMR spectroscopic data for the product was equivalent to reported literature.

^1H, ^{13}C, DEPTQ135, COSY, DOSY and HSQC NMR spectra were recorded on an AV300 or AV 400 MHz spectrometer. All ^{13}C spectra were proton decoupled. Chemical shifts (δ in ppm) in the ^1H and ^{13}C NMR spectra were referenced to the residual signals of the deuterated solvents. Common abbreviations have been used to describe signal multiplicities: s (singlet), d (doublet), t (triplet), q (quartet), dd (doublet of a doublet), m (multiplet) and br (broad).

Catalytic reactions were performed as follows: standard solutions were made up of each catalyst in deuterated solvents (C_6D_6, THF-d_8). The catalyst (0.03 mmol for 10 mol%;) was added into a J Youngs tube along with 0.45 mL of the deuterated solvent (C_6D_6 or THF-d_8). The chosen alkene substrate (0.3 mmol) was subsequently added to the J Youngs tube along with a suitable standard (tetramethylsilane) and 1.5 molar equivalents of 1,4-cyclohexadiene (0.45 mmol). The reaction could then be heated to its desired temperature and monitored via NMR spectroscopy until it reached completion. ^1H NMR spectra were recorded every 30 minutes capped at a total time of 24 hours.

For crystallographic determination, single crystals were layered with perfluoropolyalkylether oil before mounting on the X-Ray diffractometer. The oil-coated crystals started bubbling on the glass slide as soon as they contacted the atmosphere. Care was taken to quickly mount a single crystal on the goniometer. Data for all compounds were measured with a Rigaku Synergy-I diffractometer using monochromated λ = 1.54184 Å radiation. Experimental details can be found in table S1. The Me_6TREN ligand is disordered by a rotation about the Na1-N4 axis. This has been modelled by assigning all C and H atoms of the Me_6TREN ligand to two positions. Occupancies refined to 0.623: 0.377. Appropriate restraints were applied to the bond lengths of the disordered groups to ensure that these approximated to expected geometry.

Synthesis of Li-1,2-*t*Bu-DH(DMAP)

DMAP (0.366 g, 3 mmol) was added to a Schlenk flask along with hexane (15 ml). The solution mixture was cooled to 0 °C using an ice bath and then *t*BuLi (1.7 M in pentane, 1.76 ml, 3mmol) was added dropwise via syringe. The resultant cloudy yellow solution was left to stir for 2 hours producing an off-yellow suspension. The solvent was then removed via cannula filtration and the white solid was dried under reduced pressure. The compound was then transferred into the glove box, weighed and stored at -20 °C in the freezer as a off-white powder material (0.55 g, 90% yield).

^{1}H NMR [400.03 MHz, 300 K, C_6D_6]: δ 1.34 (s, 9H, *t*Bu), 2.07 (s, 30H, TREN), 2.91 (s, 6H, NMe_2), 3.82 (dd, 1H, C1-H), 4.09 (d, 1H, C2-H), 4.70 (dd, 1H, C4-H), 6.88 ppm (d, 1H, C5-H); ^{13}C {^{1}H} NMR [100.60 MHz, 300 K, C_6D_6]: δ 150.90 (C5-H), 81.00 (C4-H), 74.50 (C1-H), 69.06 (C2-H), 56.52 (CH_2 Me_6TREN), 51.02 (CH_2 Me_6TREN), 44.77 (CH_3 Me_6TREN), 41.22 (NMe_2), 40.85 (quaternary[DH(DMAP)]), 25.08 ppm (*t*Bu).

Supplementary Table 1 Selected crystallographic data collection and refinement parameters.

	**1·Me$_6$TREN**
Empirical Formula	$C_{23}H_{49}N_6Na$
MW	432.67
Crystal system	Monoclinic
Space group	C 2/c
a/Å	27.7658(4)
b/Å	11.2429(2)
c/Å	18.0148(3)
α/°	90
β/°	102.141(2)
γ/°	90
V/Å^3	5497.86(16)
Z	8
ρ/gcm^{-3}	1.045
Reflns measured	25987
Unique reflns	5482
R_{int}	0.0268
Obs. reflns [I>2σI]	4562
GooF	1.051
R	0.0654
ω*R*	0.0743
Largest diff peak/hole eÅ^{-3}	0.645/-0.255

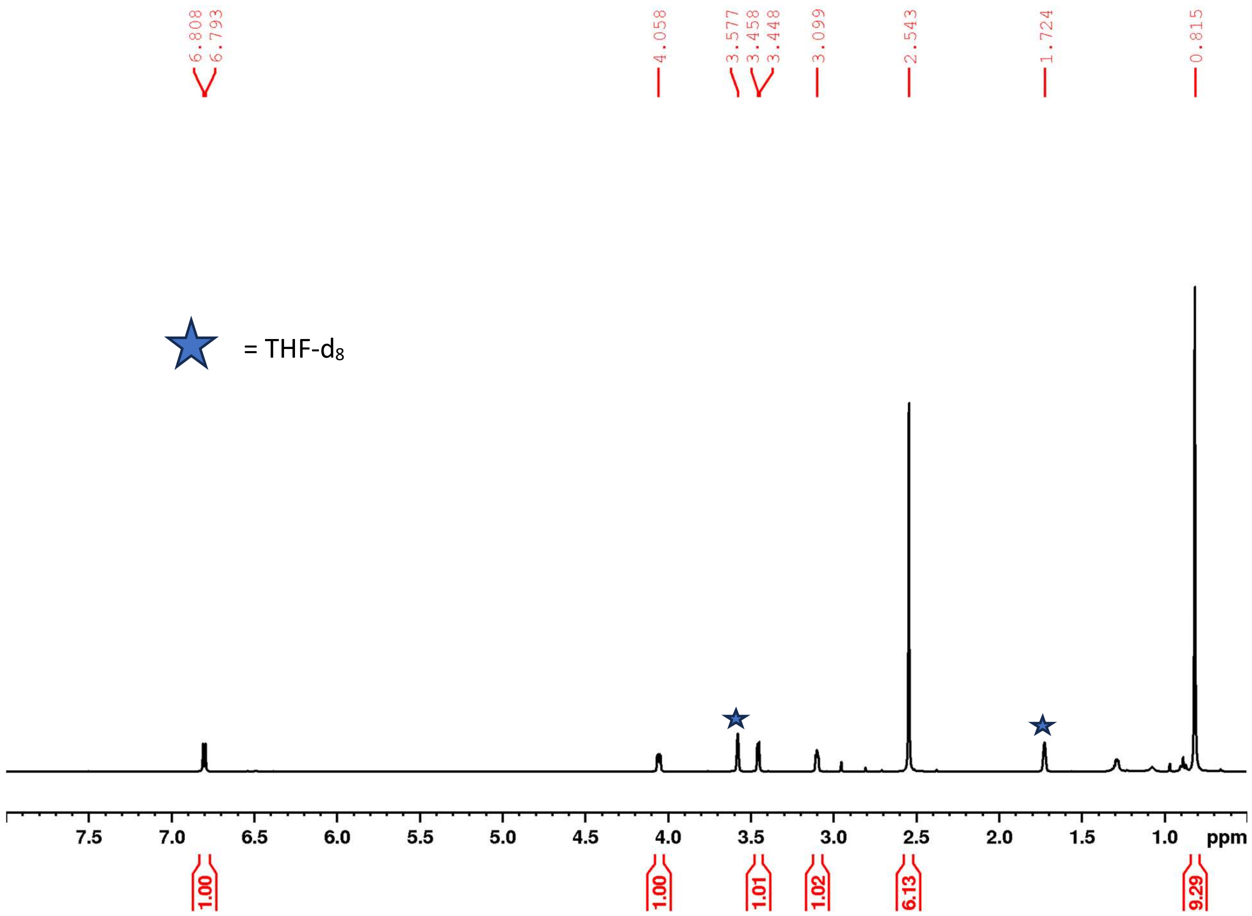

Supplementary Figure 1 – ^{1}H NMR spectrum of Na-1,2-*t*Bu-DH(DMAP) (**1**) in THF-d_8.

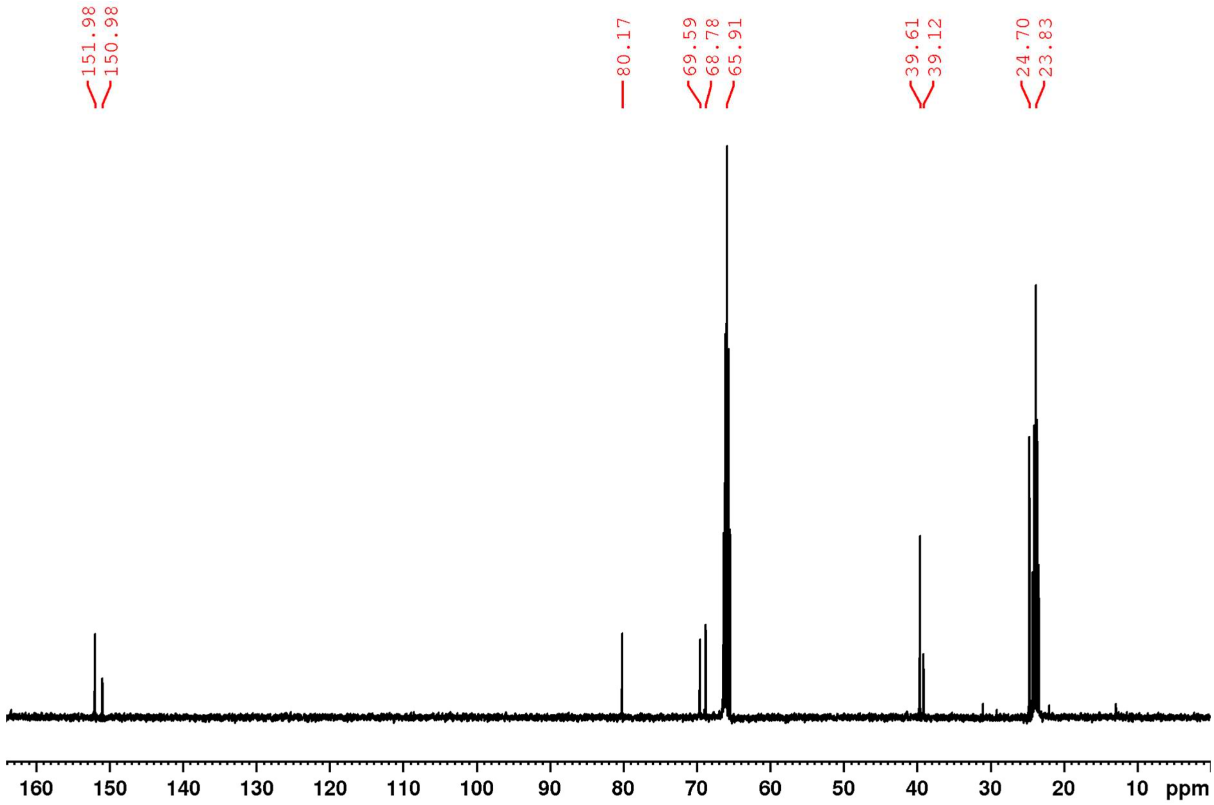

Supplementary Figure 2 – ^{13}C NMR spectrum of Na-1,2-*t*Bu-DH(DMAP) (**1**) in THF-d_8.

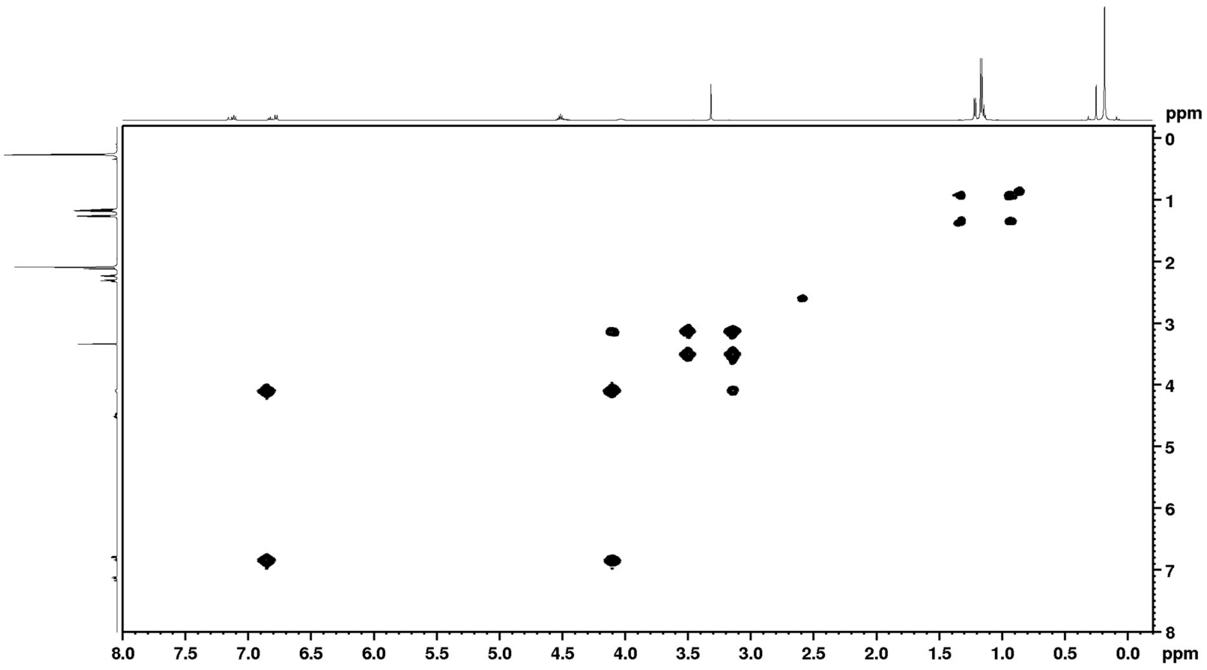

Supplementary Figure 3 – ^1H-^1H COSY NMR spectrum of Na-1,2-*t*Bu-DH(DMAP) (**1**) in THF-d_8.

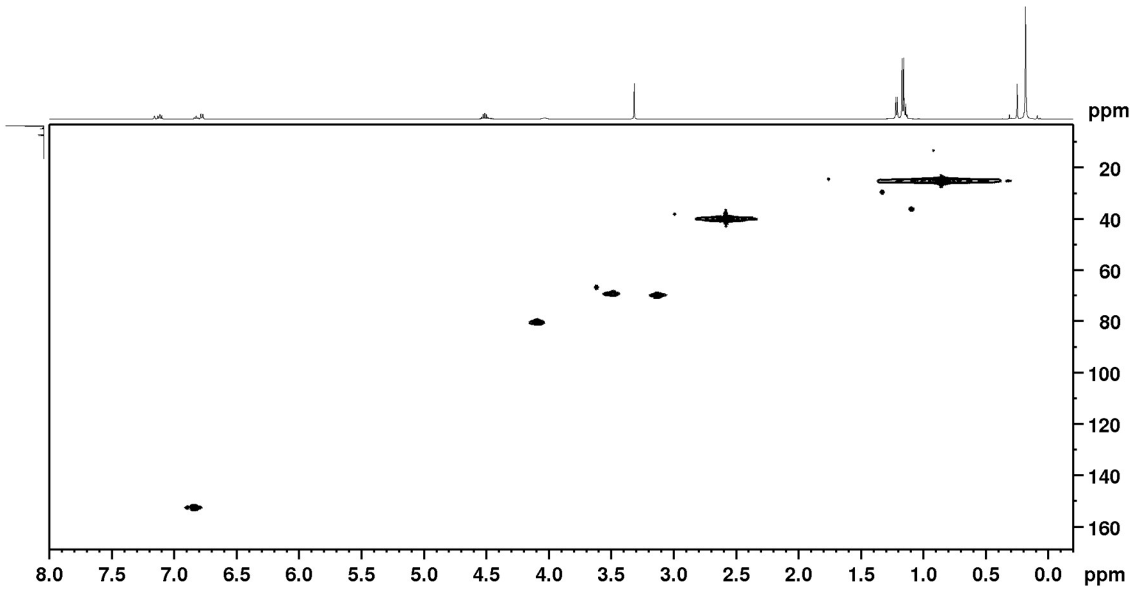

Supplementary Figure 4 – ^1H-^{13}C HSQC NMR spectrum of Na-1,2-*t*Bu-DH(DMAP) (**1**) in THF-d_8.

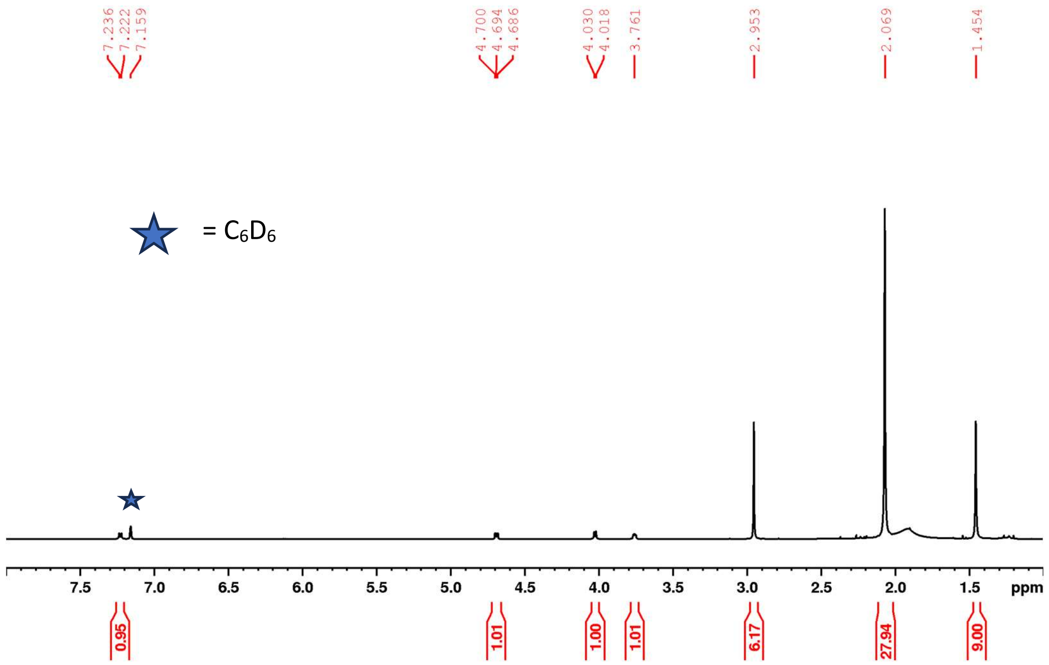

Supplementary Figure 5 – ^{1}H NMR spectrum of [Na-1,2-*t*Bu-DH(DMAP)]·Me_6TREN (**1·Me_6TREN**) in C_6D_6.

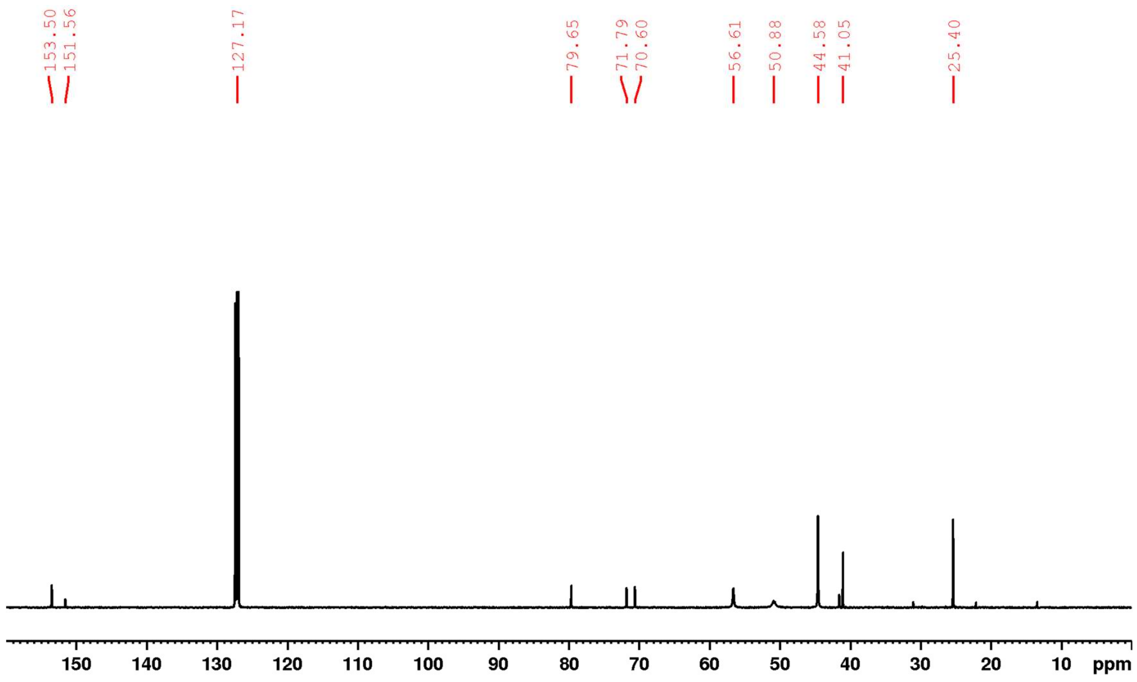

Supplementary Figure 6 – ^{13}C NMR spectrum of [Na-1,2-*t*Bu-DH(DMAP)]·Me_6TREN (**1·Me_6TREN**) in C_6D_6.

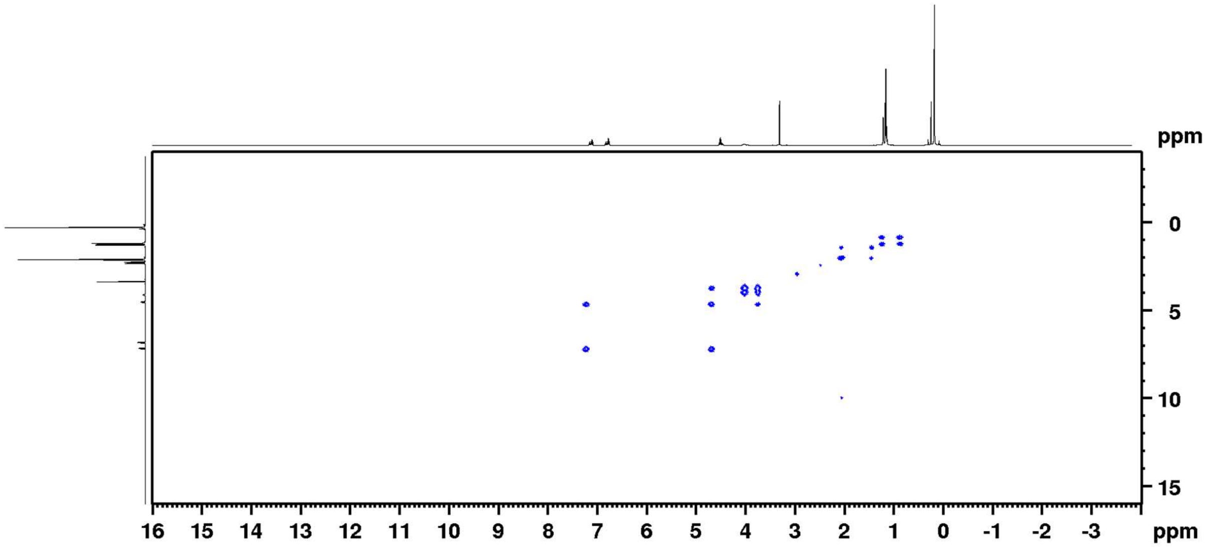

Supplementary Figure 7 – ^{1}H-^{1}H COSY NMR spectrum of [Na-1,2-*t*Bu-DH(DMAP)]·Me_6TREN (**1·Me_6TREN**) in C_6D_6.

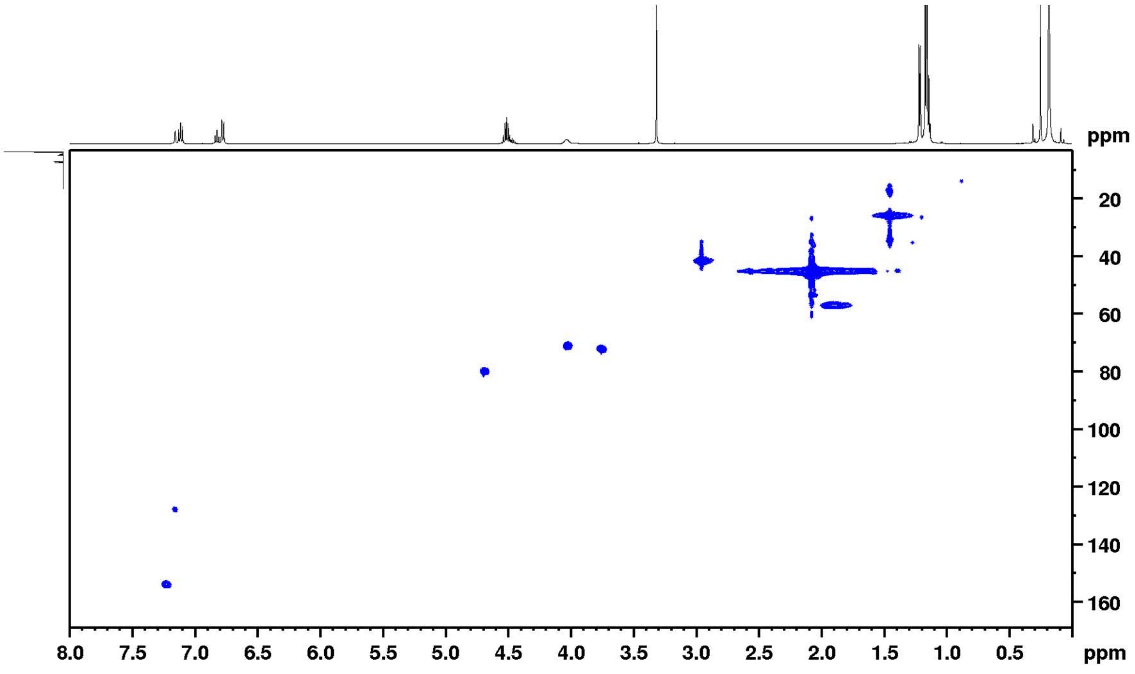

Supplementary Figure 8 – ^{1}H-^{13}C HSQC NMR spectrum of [Na-1,2-*t*Bu-DH(DMAP)]·Me_6TREN (**1·Me_6TREN**) in C_6D_6.

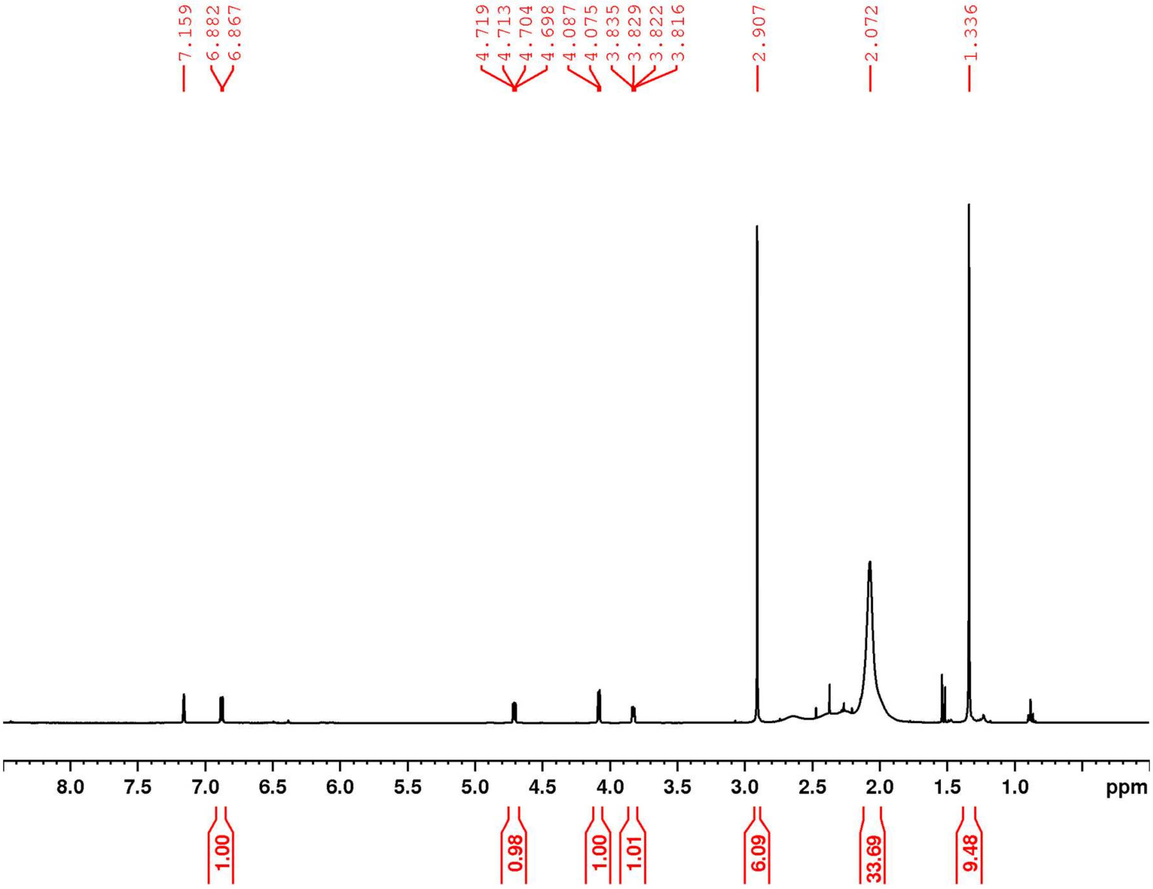

Supplementary Figure 9 - ^{1}H NMR spectrum of [Li-1,2-*t*Bu-DH(DMAP)]·Me_6TREN in C_6D_6.

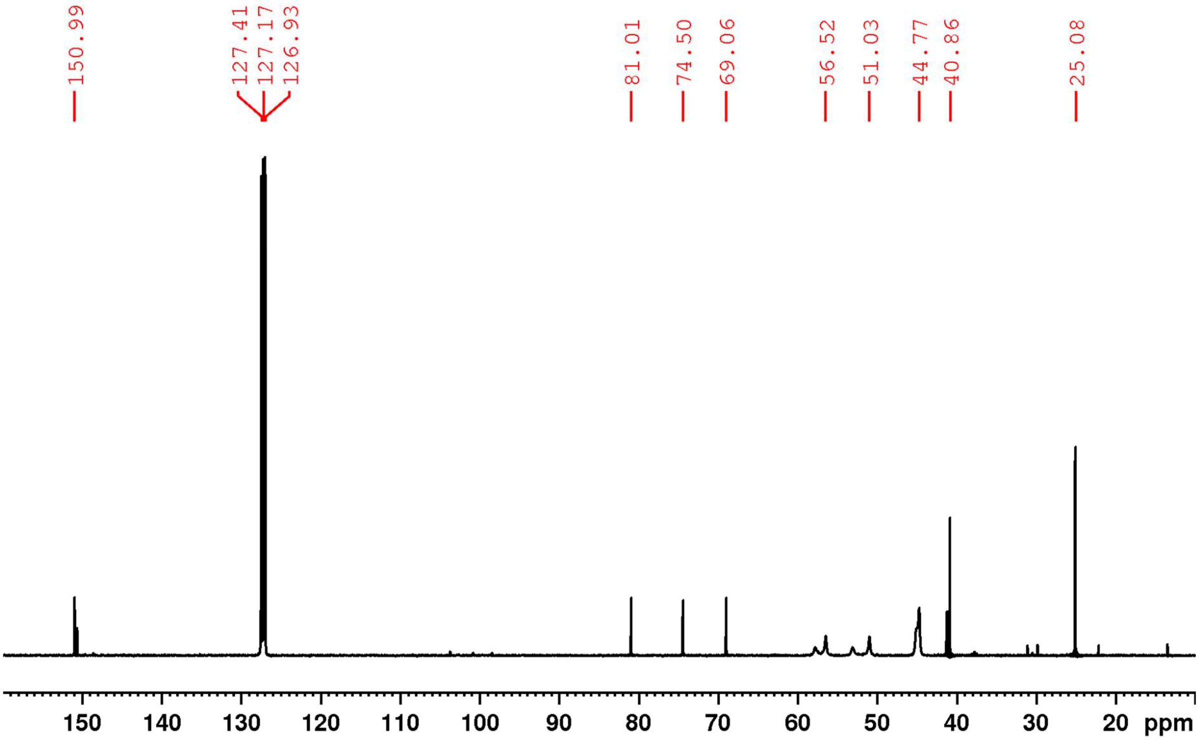

Supplementary Figure 10 - ^{13}C NMR spectrum of [Li-1,2-*t*Bu-DH(DMAP)]·Me_6TREN in C_6D_6.

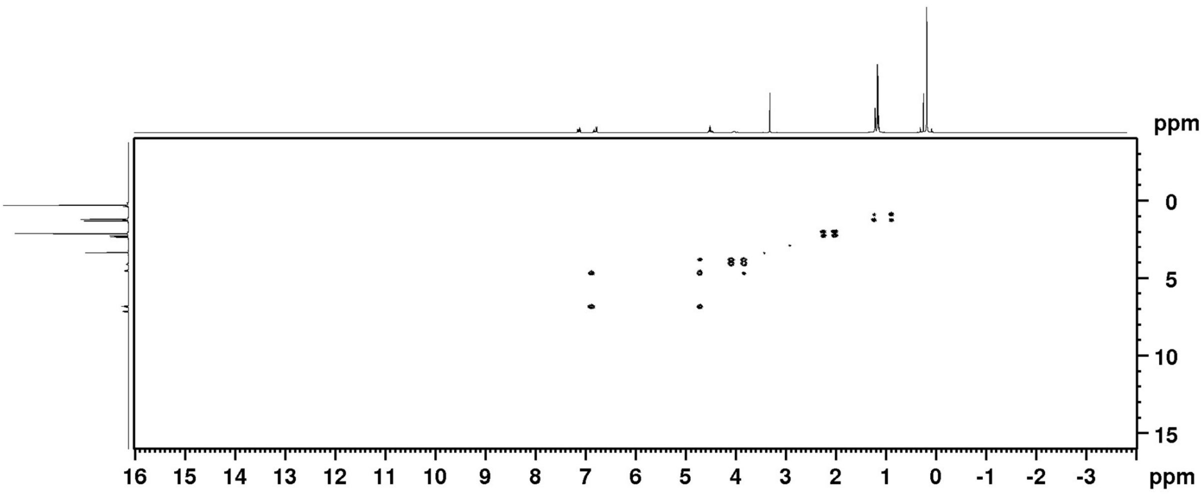

Supplementary Figure 11 - ^{1}H-^{1}H COSY NMR spectrum of [Li-1,2-*t*Bu-DH(DMAP)]·Me_6TREN in C_6D_6.

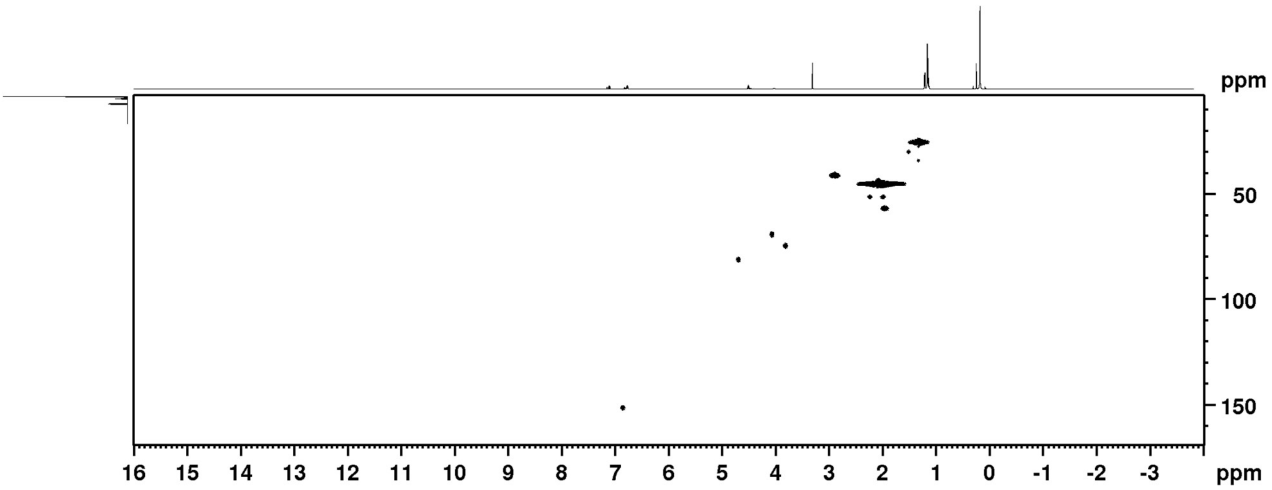

Supplementary Figure 12 - ^{1}H-^{13}C HSQC NMR spectrum of [Li-1,2-*t*Bu-DH(DMAP)]·Me_6TREN in C_6D_6.

2. DOSY (Diffusion Ordered Spectroscopy) studies

In order to estimate the aggregation state of Na-1,2-*t*Bu-DH(DMAP) (**1**) and Na-1,2-*t*Bu-DH(DMAP)·Me_6TREN (**1·Me_6TREN**) in solution, we estimated its molecular weight (MW) using ^{1}H DOSY. The $ECC\frac{DSE}{THF}$ (external calibration curve for ECC dissipated spheres and ellipsoids in THF-d_8 or C_6D_6) or $ECC\frac{MERGE}{THF}$ (external calibration curve for ECC merger between dissipated spheres and ellipsoids and contacted spheres in THF-d_8 or C_6D_6) was suitable to estimate the MW of both compounds[3].

2.1 Na-1,2-*t*Bu-DH(DMAP)

^{1}H DOSY-ECC-MW estimation of Na-1,2-*t*Bu-DH(DMAP) (**1**) in THF-d_8 was carried out at 298 K. Tetramethylsilane (TMS) was used as an internal reference.

Supplementary Table 2 Diffusion coefficient analysis using ^{1}H DOSY NMR data obtained for a mixture of compound **1** and TMS (internal reference) at 298 K in THF-d_8.

Peak name	F2 [ppm]	Io	error	D [m2/s]	error
1	6.835	1.66e+09	6.933e+06	9.22e-10	8.791e-12
2	4.093	1.80e+09	7.411e+06	9.13e-10	8.600e-12
3	3.490	2.08e+09	3.723e+06	9.26e-10	3.801e-12
4	3.134	2.16e+09	5.868e+06	9.19e-10	5.720e-12
5	2.574	1.24e+10	6.041e+07	9.07e-10	1.014e-11
6	0.850	2.05e+10	7.517e+07	9.28e-10	7.789e-12
7	0.024	6.86e+09	6.516e+07	2.38e-09	5.079e-11

Supplementary Table 3 Average diffusion coefficient analysis using ^{1}H DOSY NMR data obtained for a mixture of compound **1** and TMS (internal reference) at 298 K in THF-d_8.

Compound	D m^2/s	Log D
TMS	$2.38e^{-09}$	-8.623423043
Na-1,2-*t*Bu-DH(DMAP)	$9.192e^{-10}$	-9.036589984

Supplementary Table 4 $ECC\frac{DSE}{THF}$ $and\ ECC\frac{MERGE}{THF}$ were used to determine the MW_{det} of compound **1** in THF-d_8 at 298 K and the MW_{dif} for the proposed species. MW_{det} extracted from ^{1}H DOSY data. Note: max. error = ± 8.

**Aggregate**	**MW_{det}(^{1}H-DOSY)**	**MW_{cal} (theoretical)**	**MW_{dif} [%]**
[Na-1,2-*t*Bu-DH(DMAP)]$_2$ (MERGE)	549	404	-26
[Na-1,2-*t*Bu-DH(DMAP)]$_2$ (DSE)	508	404	-20
**[Na-1,2-*t*Bu-DH(DMAP)]$_2$·2THF (MERGE)**	**549**	**564**	**3**
[Na-1,2-*t*Bu-DH(DMAP)]$_2$·2THF (DSE)	508	564	11

Supplementary Table 5 $ECC\frac{DSE}{THF}$ was used to determine the MW_{det} of compound **1** in THF-d_8 at 298 K and the MW_{dif} for the proposed $ECC\frac{DSE}{THF}$species. MW_{det} extracted from ^{1}H DOSY data. Note: max. error = ± 8.

**Aggregate**	**MW_{det}(^{1}H-DOSY)**	**MW_{cal} (theoretical)**	**MW_{dif} [%]**
[Na-1,2-*t*Bu-DH(DMAP)]·2THF	508	362	-26
[Na-1,2-*t*Bu-DH(DMAP)]·3THF	508	442	-13
**[Na-1,2-*t*Bu-DH(DMAP)]·4THF**	**508**	**522**	**3**

Supplementary Table 6 $ECC\frac{MERGE}{THF}$ was used to determine the MW_{det} of compound **1** in THF-d_8 at 298 K and the MW_{dif} for the proposed $ECC\frac{MERGE}{THF}$species. MW_{det} extracted from ^{1}H DOSY data. Note: max. error = ± 8.

**Aggregate**	**MW_{det}(^{1}H-DOSY)**	**MW_{cal} (theoretical)**	**MW_{dif} [%]**
[Na-1,2-*t*Bu-DH(DMAP)]·2THF	549	362	-34
[Na-1,2-*t*Bu-DH(DMAP)]·3THF	549	442	-19
**[Na-1,2-*t*Bu-DH(DMAP)]·4THF**	**549**	**522**	**-5**

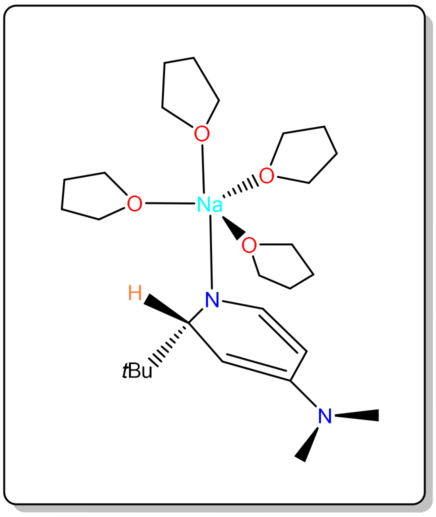

Supplementary Figure 13 – Proposed solution state structure from ^{1}H DOSY NMR spectroscopic analysis of [Na-1,2-*t*Bu-DH(DMAP)]·4THF at 298 K in THF-d_8.

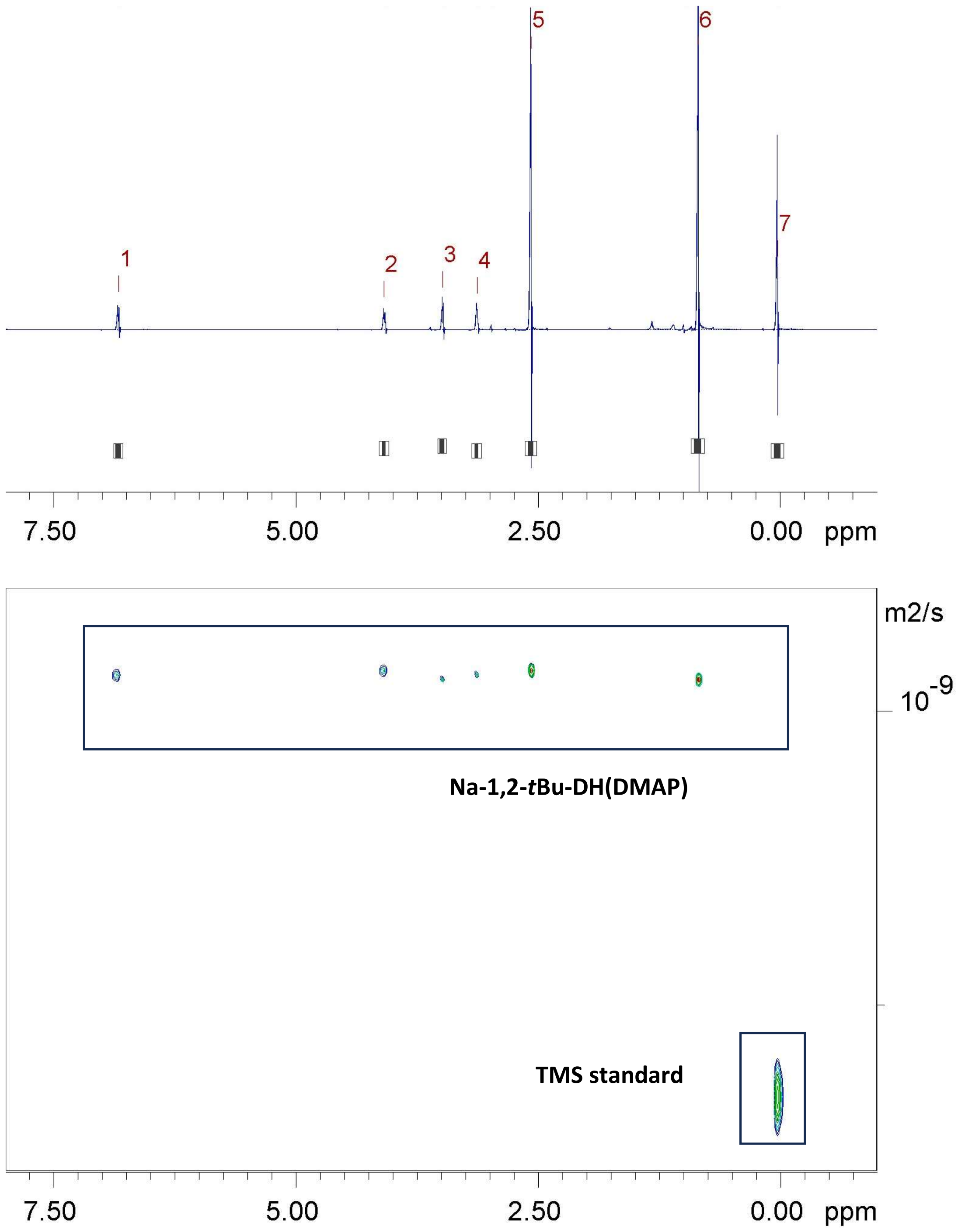

Supplementary Figure 14 – ^{1}H NMR and DOSY correlation analysis of [Na-1,2-*t*Bu-DH(DMAP)]·4THF at 298 K in THF-d_8.

2.2 Na-1,2-*t*Bu-DH(DMAP)·Me_6TREN (1·Me_6TREN)

^{1}H DOSY-ECC-MW estimation of Na-1,2-*t*Bu-DH(DMAP)·Me_6TREN (**1·Me_6TREN**) in C_6D_6 was carried out at 298 K. Tetramethylsilane (TMS) was used as an internal reference.

Supplementary Table 7 Diffusion coefficient analysis using ^{1}H DOSY NMR data obtained for a mixture of compound **1·Me_6TREN** and TMS (internal reference) at 298 K in C_6D_6.

Peak name	F2 [ppm]	Io	error	D [m2/s]	error
1	7.239	1.32e+08	2.509e+05	8.22e-10	3.559e-12
2	7.146	1.65e+07	2.070e+05	2.06e-09	5.688e-11
3	4.698	1.54e+08	2.714e+05	8.16e-10	3.271e-12
4	4.023	1.70e+08	3.312e+05	8.18e-10	3.617e-12
5	3.763	1.74e+08	4.079e+05	8.14e-10	4.350e-12
6	2.949	9.78e+08	3.000e+06	8.15e-10	5.686e-12
7	2.065	3.76e+09	7.115e+06	8.87e-10	3.794e-12
8	1.443	1.39e+09	1.722e+06	8.03e-10	2.261e-12
9	-0.011	5.84e+08	3.599e+06	2.11e-09	2.864e-11

Supplementary Table 8 Average diffusion coefficient analysis using the ^{1}H DOSY data obtained for a mixture of compound **1·Me_6TREN** and TMS (internal reference) at 298 K in C_6D_6.

Compound	D m^2/s	Log D
TMS	$2.11e^{-09}$	-8.675717545
Na-1,2-*t*Bu-DH(DMAP)·Me_6TREN	$8.25e^{-10}$	-9.083546051

Supplementary Table 9 $ECC\frac{DSE}{THF}$ and $ECC\frac{MERGE}{THF}$ were used to determine the MW_{det} of compound **1·Me_6TREN** in C_6D_6 at 298 K and the MW_{dif} for the proposed species. MW_{det} extracted from ^{1}H DOSY NMR. Note: max. error = ± 8.

Aggregate	MW_{det}(^{1}H-DOSY)	MW_{cal} (theoretical)	MW_{dif} [%]
**Na-1,2-*t*Bu-DH(DMAP)·Me_6TREN (MERGE)**	**449**	**432**	**-4**
**Na-1,2-*t*Bu-DH(DMAP)·Me_6TREN (DSE)**	**413**	**432**	**5**

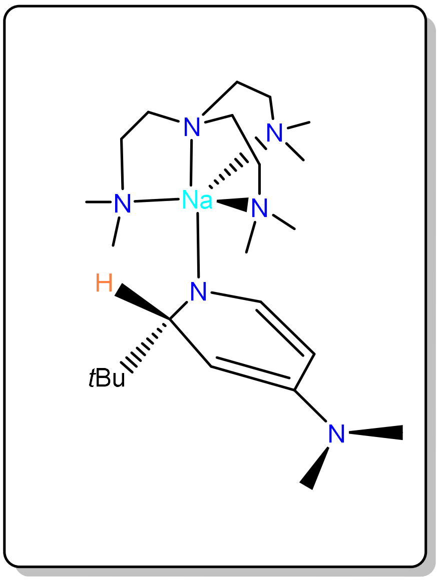

Supplementary Figure 15 – Proposed solution state structure from ^{1}H DOSY NMR spectroscopic analysis of [Na-1,2-*t*Bu-DH(DMAP)]·Me_6TREN at 298 K in C_6D_6.

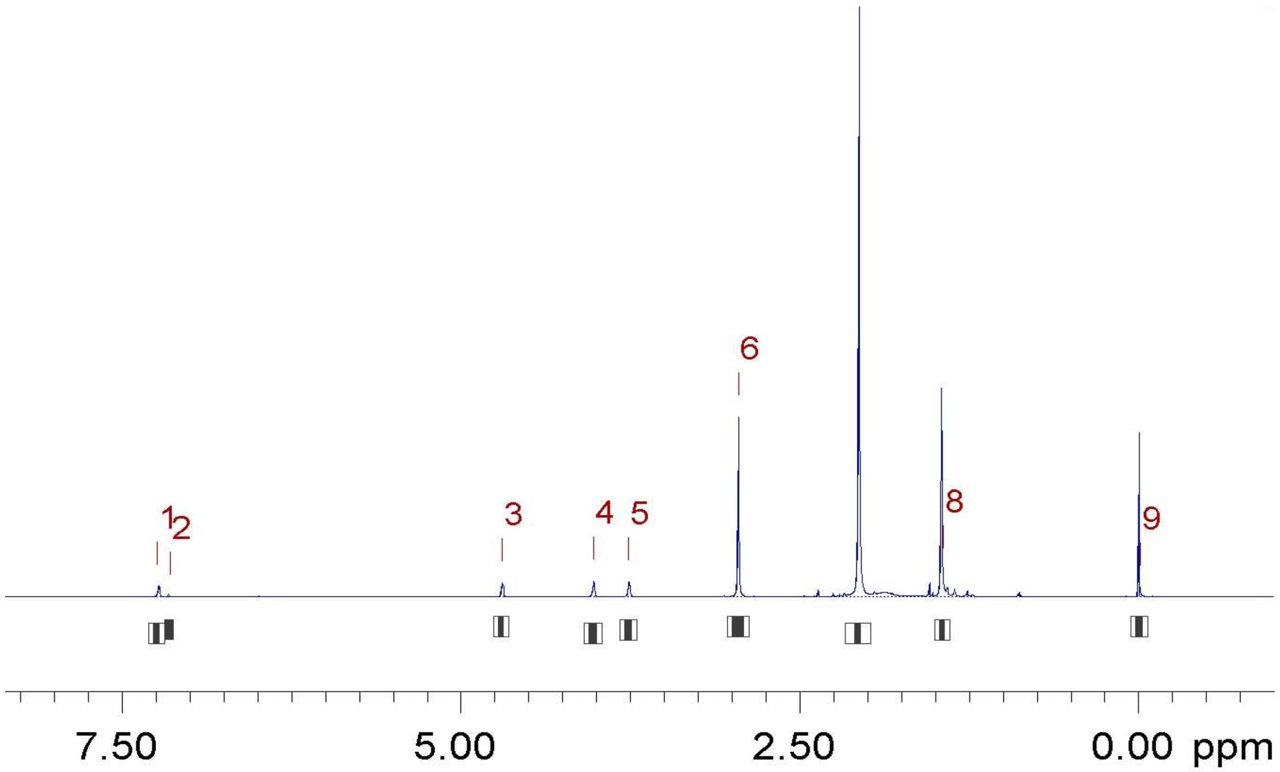

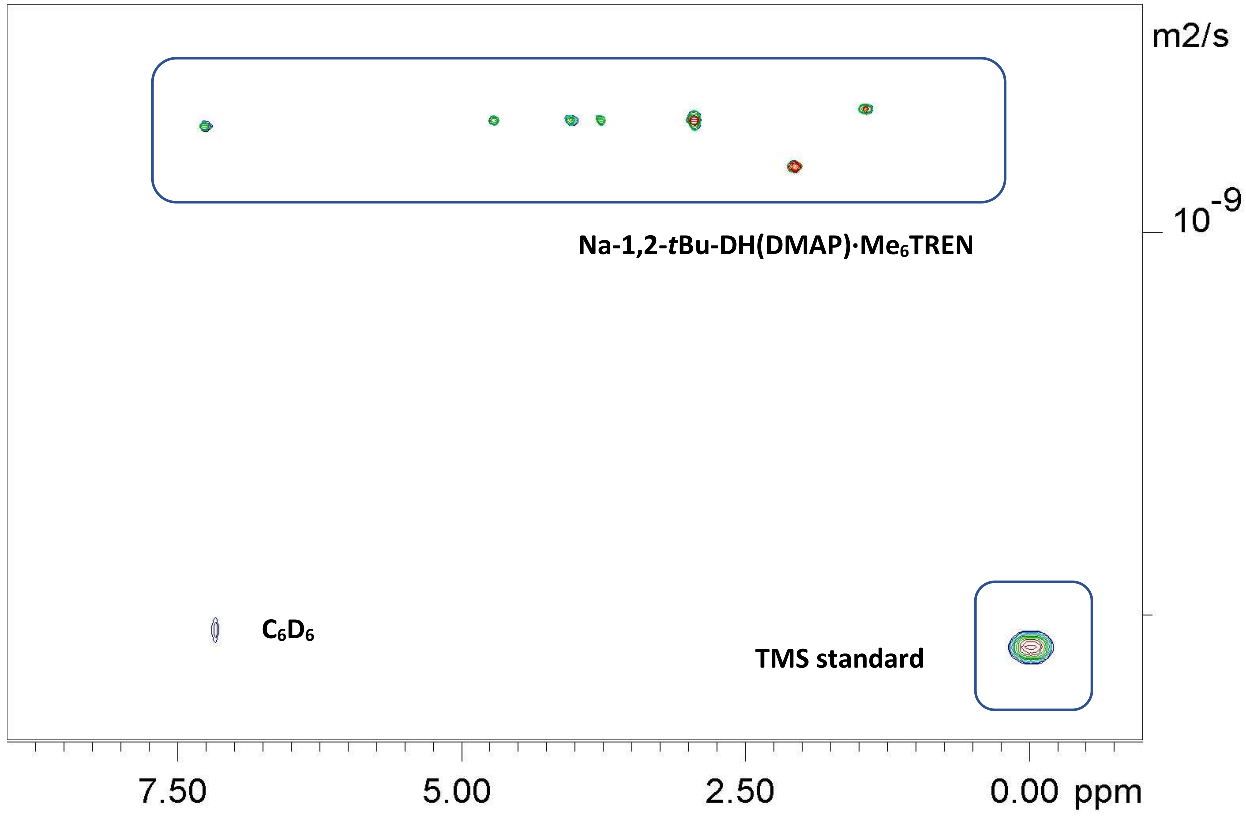

Supplementary Figure 16 – ^{1}H NMR and DOSY correlation analysis of [Na-1,2-*t*Bu-DH(DMAP)]·Me_6TREN (**1·Me_6TREN**) at 298 K in C_6D_6.

3. Transfer hydrogenation experiments

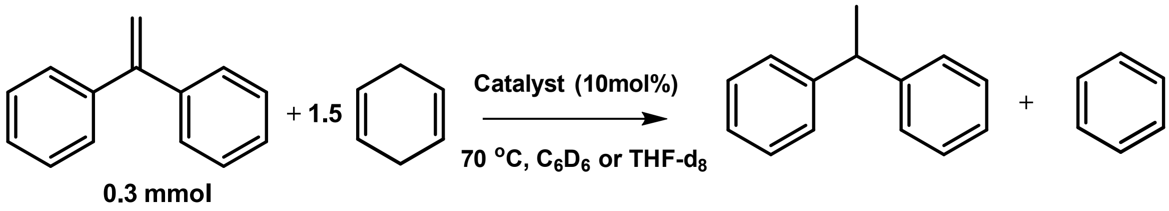

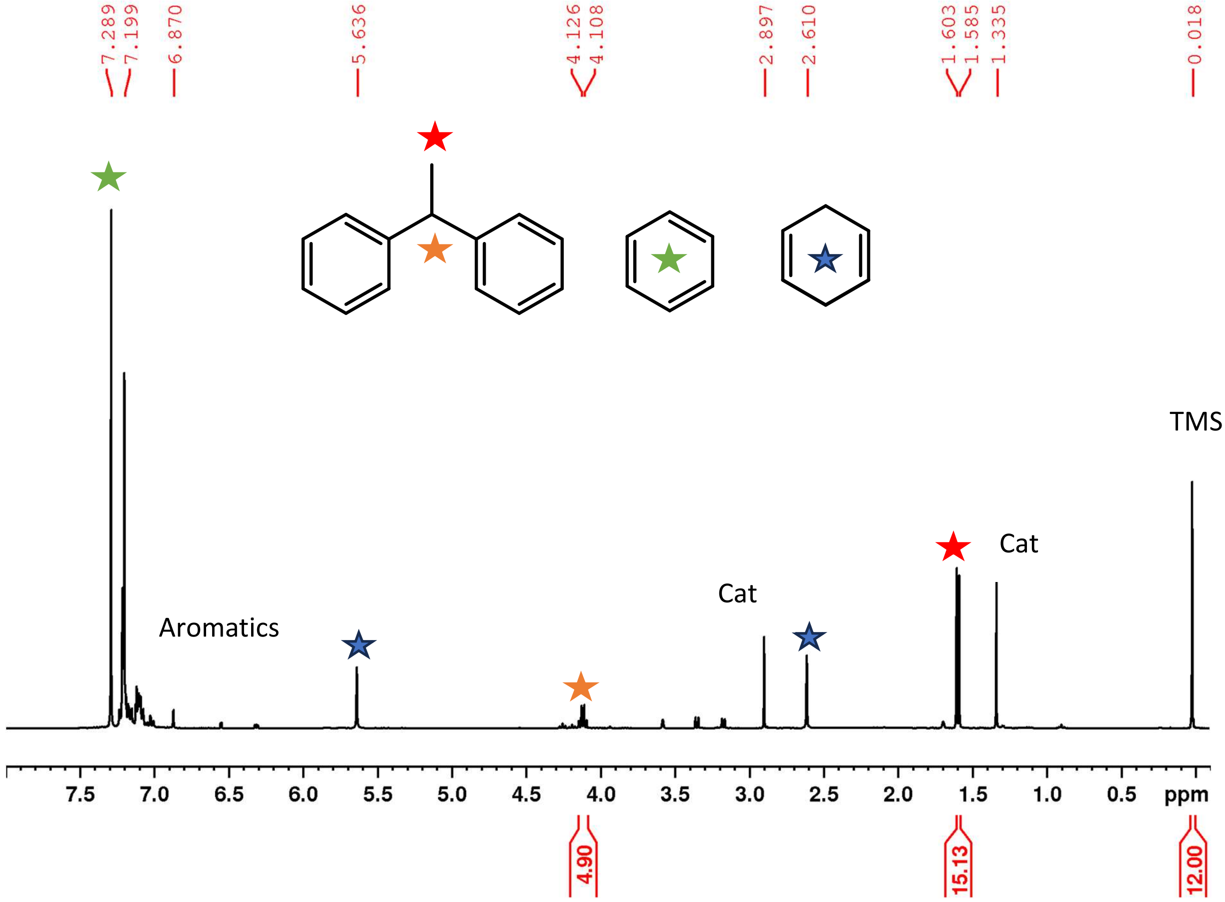

Supplementary Figure 17 – ^{1}H NMR spectrum of the completed transfer hydrogenation reaction between 1,1-diphenylethylene (0.3 mmol) and [Na-1,2-*t*Bu-DH(DMAP)] (10 mol%) as catalyst using 1.5 equiv. of 1,4-cyclohexadiene in C_6D_6 showing formation of the alkane product (1,1-diphenylethane) after heating for 24 hours at 70 °C. Tetramethylsilane standard was used (0.06 mmol) to calculate percentage yield (entry 1, yield 99%).

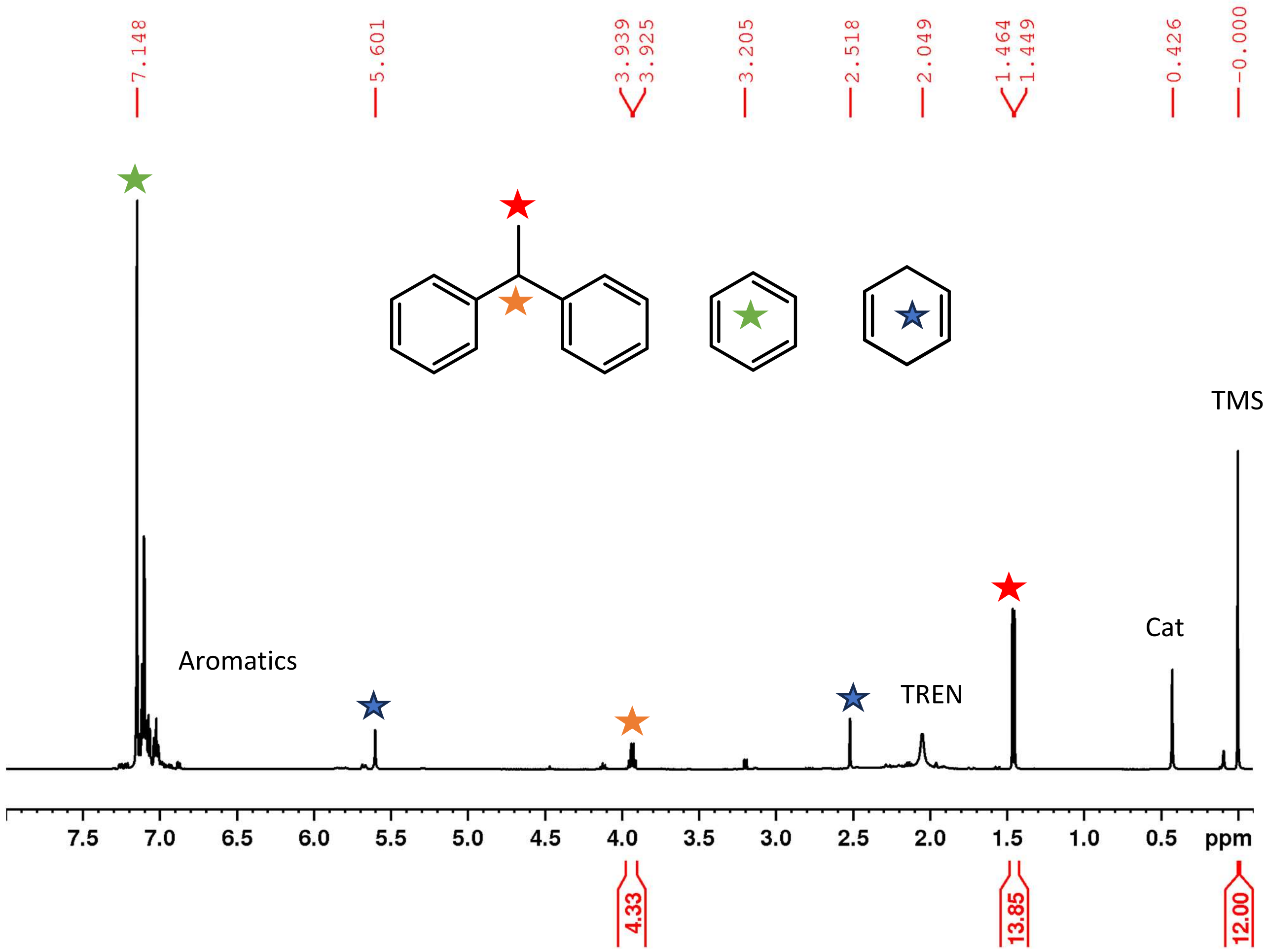

Supplementary Figure 18 – ^{1}H NMR spectrum of the completed transfer hydrogenation reaction between 1,1-diphenylethylene (0.3 mmol) and [Na-1,2-*t*Bu-DH(DMAP)]·Me_6TREN (10 mol%) as catalyst using 1.5 equiv. of 1,4-cyclohexadiene in C_6D_6 showing formation of the alkane product (1,1-diphenylethane) after heating for 0.5 hours at 70 °C. Tetramethylsilane standard was used (0.06 mmol) to calculate percentage yield (entry 2, yield 93%).

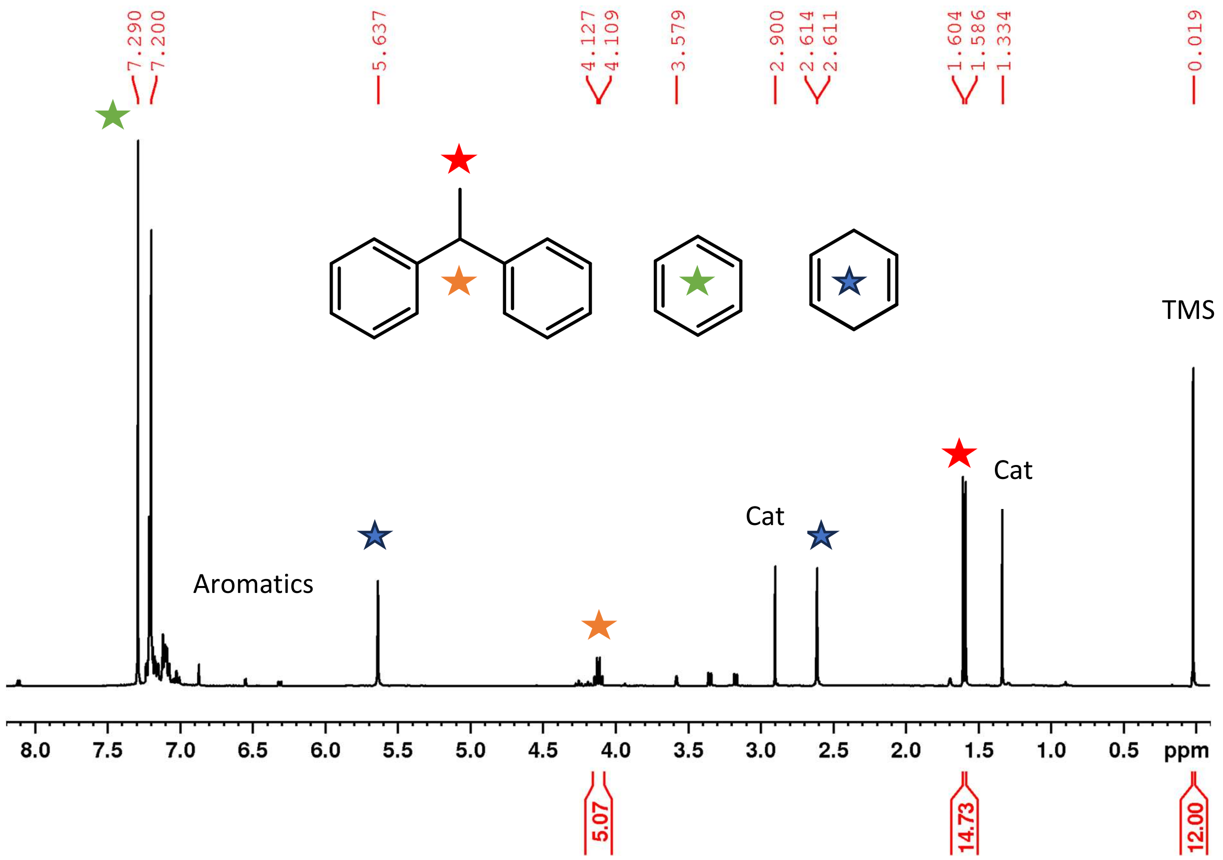

Supplementary Figure 19 – ^{1}H NMR spectrum of the completed transfer hydrogenation reaction between 1,1-diphenylethylene (0.3 mmol) and [Na-1,2-*t*Bu-DH(DMAP)] (10 mol%) as catalyst using 1.5 equiv. of 1,4-cyclohexadiene in THF-D_8 showing formation of the alkane product (1,1-diphenylethane) after heating for 0.5 hours at 70 °C. Tetramethylsilane standard was used (0.06 mmol) to calculate percentage yield (entry 3, yield 98%).

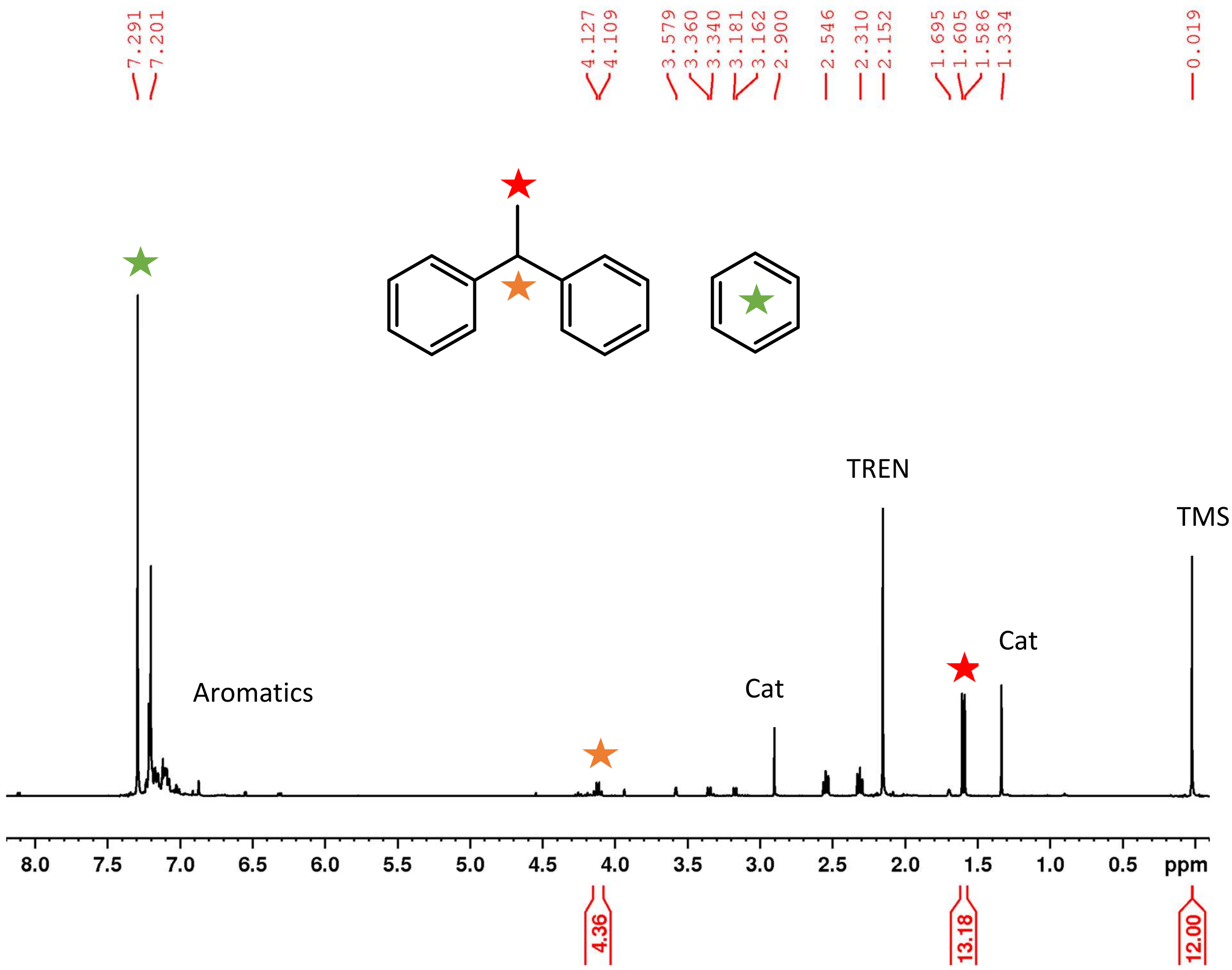

Supplementary Figure 20 – ^{1}H NMR spectrum of the completed transfer hydrogenation reaction between 1,1-diphenylethylene (0.3 mmol) and [Na-1,2-*t*Bu-DH(DMAP)]·Me$_6$TREN (10 mol%) as catalyst using 1.5 equiv. of 1,4-cyclohexadiene in THF-D$_8$ showing formation of the alkane product (1,1-diphenylethane) after heating for 1 hour at 70 °C. Tetramethylsilane standard was used (0.06 mmol) to calculate percentage yield (entry 4, yield 89%).

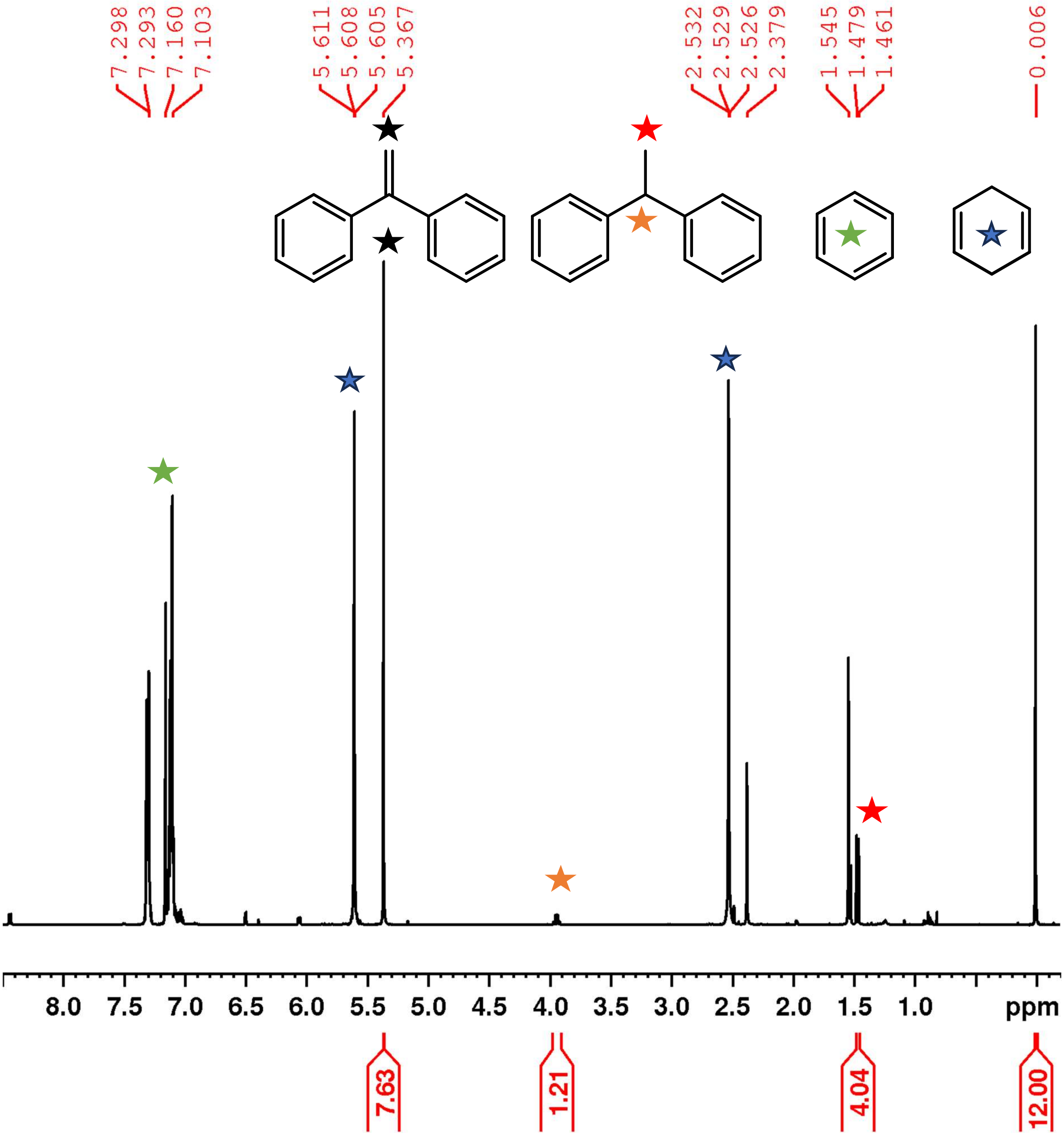

Supplementary Figure 21 - ^{1}H NMR spectrum of the completed transfer hydrogenation reaction between 1,1-diphenylethylene (0.3 mmol) and [Li-1,2-*t*Bu-DH(DMAP)] (10 mol%) as catalyst using 1.5 equiv. of 1,4-cyclohexadiene in C_6D_6 showing formation of the alkane product (1,1-diphenylethane) after heating for 24 hours at 70 °C. Tetramethylsilane standard was used (0.06 mmol) to calculate the percentage yield (entry 5, yield 24%).

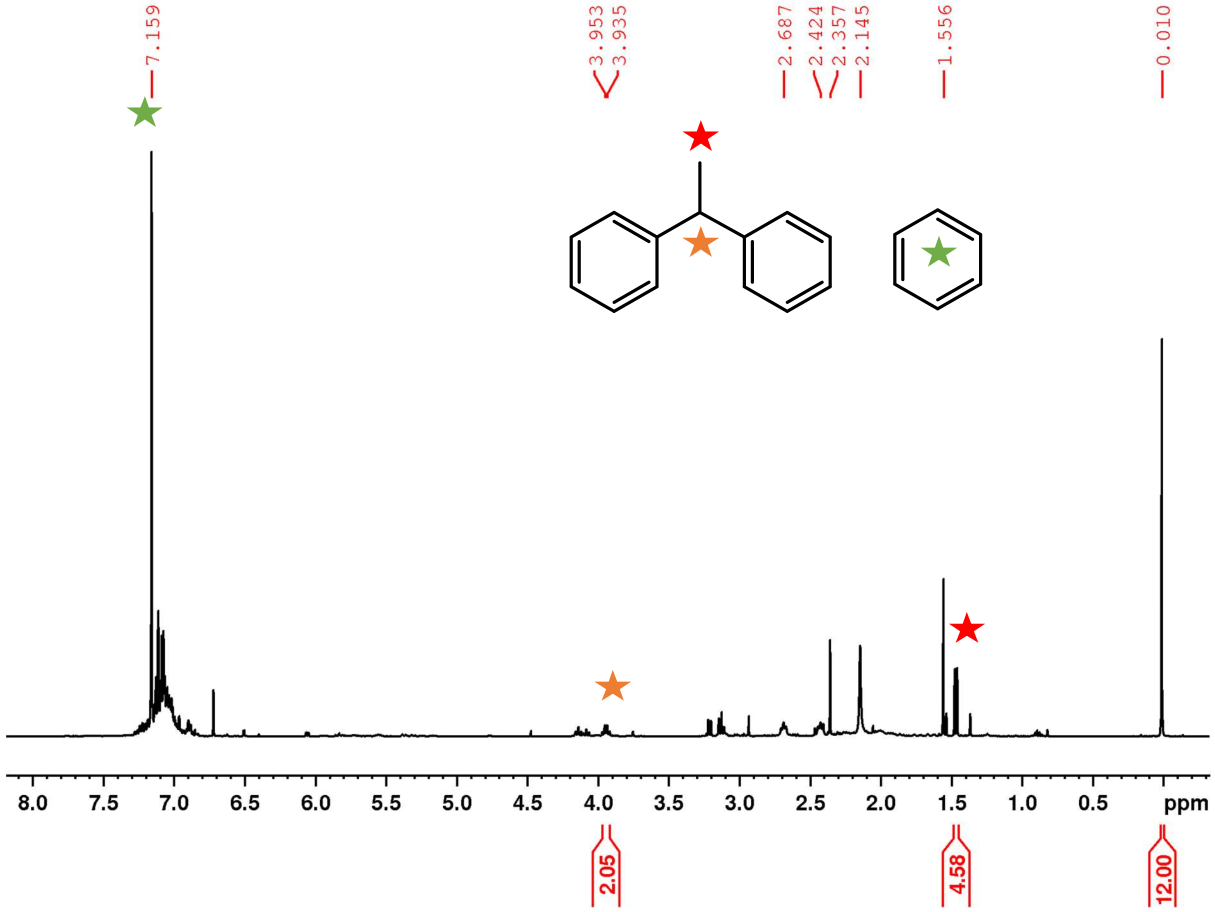

Supplementary Figure 22 - ^{1}H NMR spectrum of the completed transfer hydrogenation reaction between 1,1-diphenylethylene (0.3 mmol) and [Li-1,2-*t*Bu-DH(DMAP)]·Me_6TREN (10 mol%) as catalyst using 1.5 equiv. of 1,4-cyclohexadiene in C_6D_6 showing formation of the alkane product (1,1-diphenylethane) after heating for 24 hours at 70 °C. Tetramethylsilane standard was used (0.06 mmol) to calculate the percentage yield (entry 6, yield 31%).

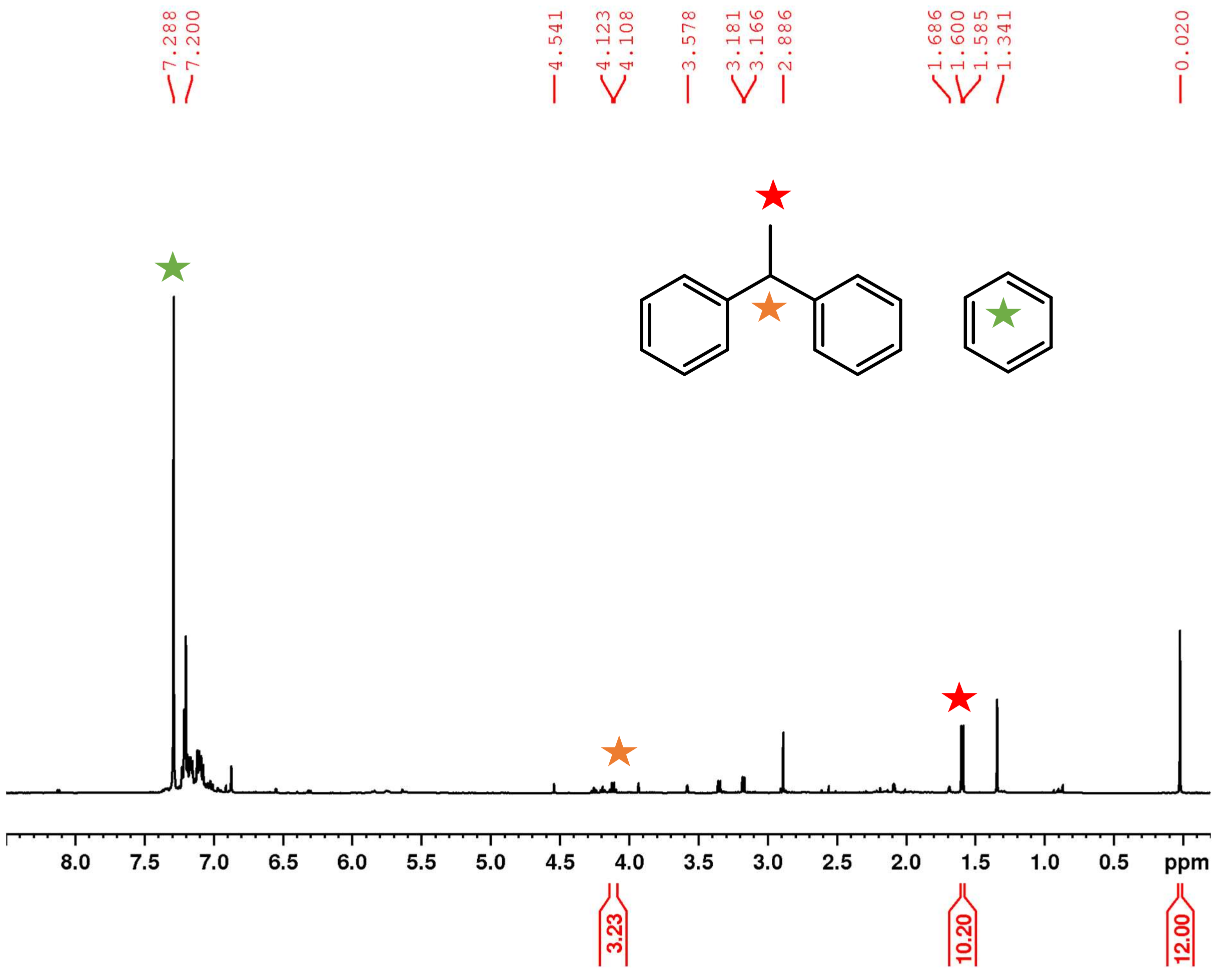

Supplementary Figure 23 - ^{1}H NMR spectrum of the completed transfer hydrogenation reaction between 1,1-diphenylethylene (0.3 mmol) and [Li-1,2-*t*Bu-DH(DMAP)] (10 mol%) as catalyst using 1.5 equiv. of 1,4-cyclohexadiene in THF-D_8 showing formation of the alkane product (1,1-diphenylethane) after heating for 6 hours at 70 °C. Tetramethylsilane standard was used (0.06 mmol) to calculate the percentage yield (entry 7, yield 68%).

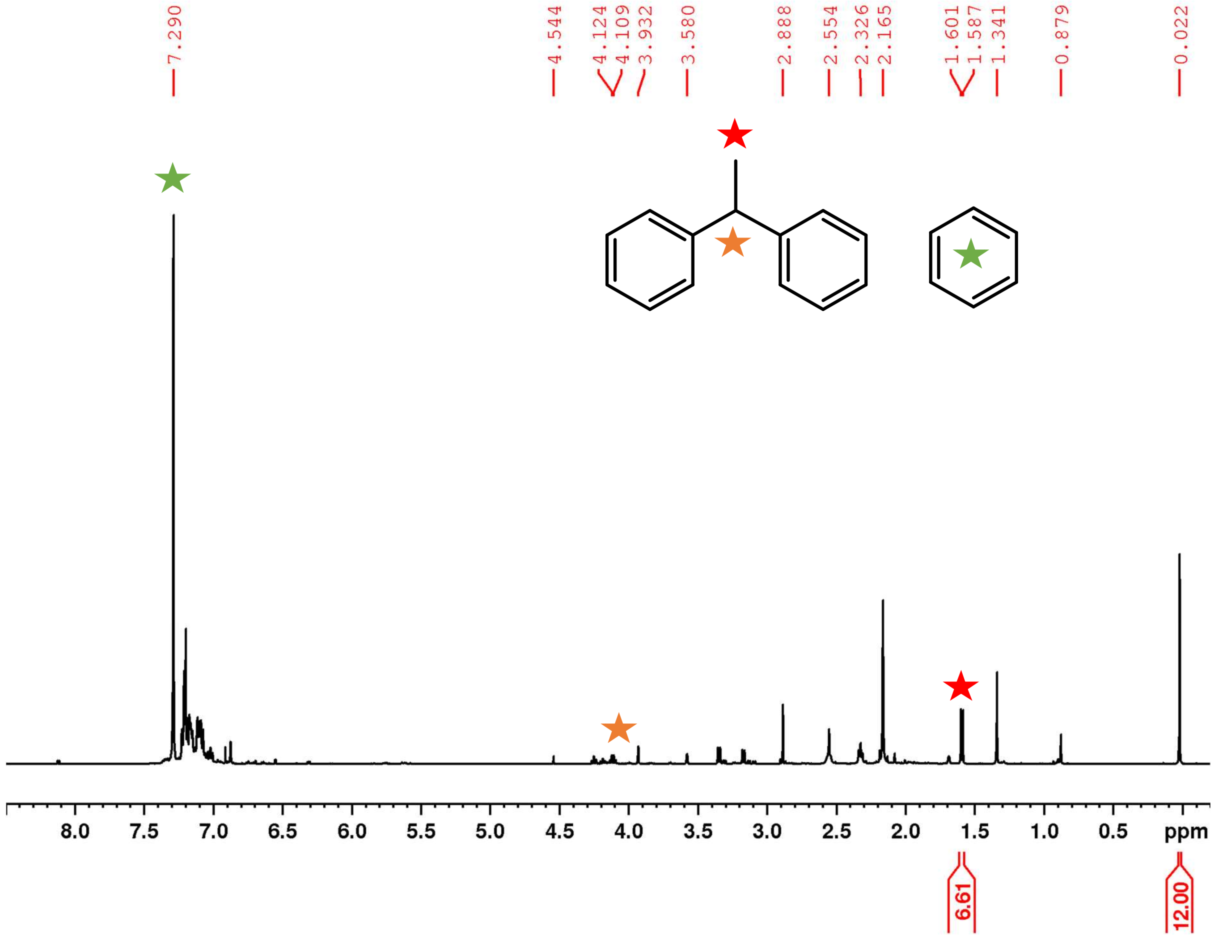

Supplementary Figure 24 - ^{1}H NMR spectrum of the completed transfer hydrogenation reaction between 1,1-diphenylethylene (0.3 mmol) and [Li-1,2-*t*Bu-DH(DMAP)]·Me_6TREN (10 mol%) as catalyst using 1.5 equiv. of 1,4-cyclohexadiene in THF-D_8 showing formation of the alkane product (1,1-diphenylethane) after heating for 3 hours at 70 °C. Tetramethylsilane standard was used (0.06 mmol) to calculate the percentage yield (entry 8, yield 44%).

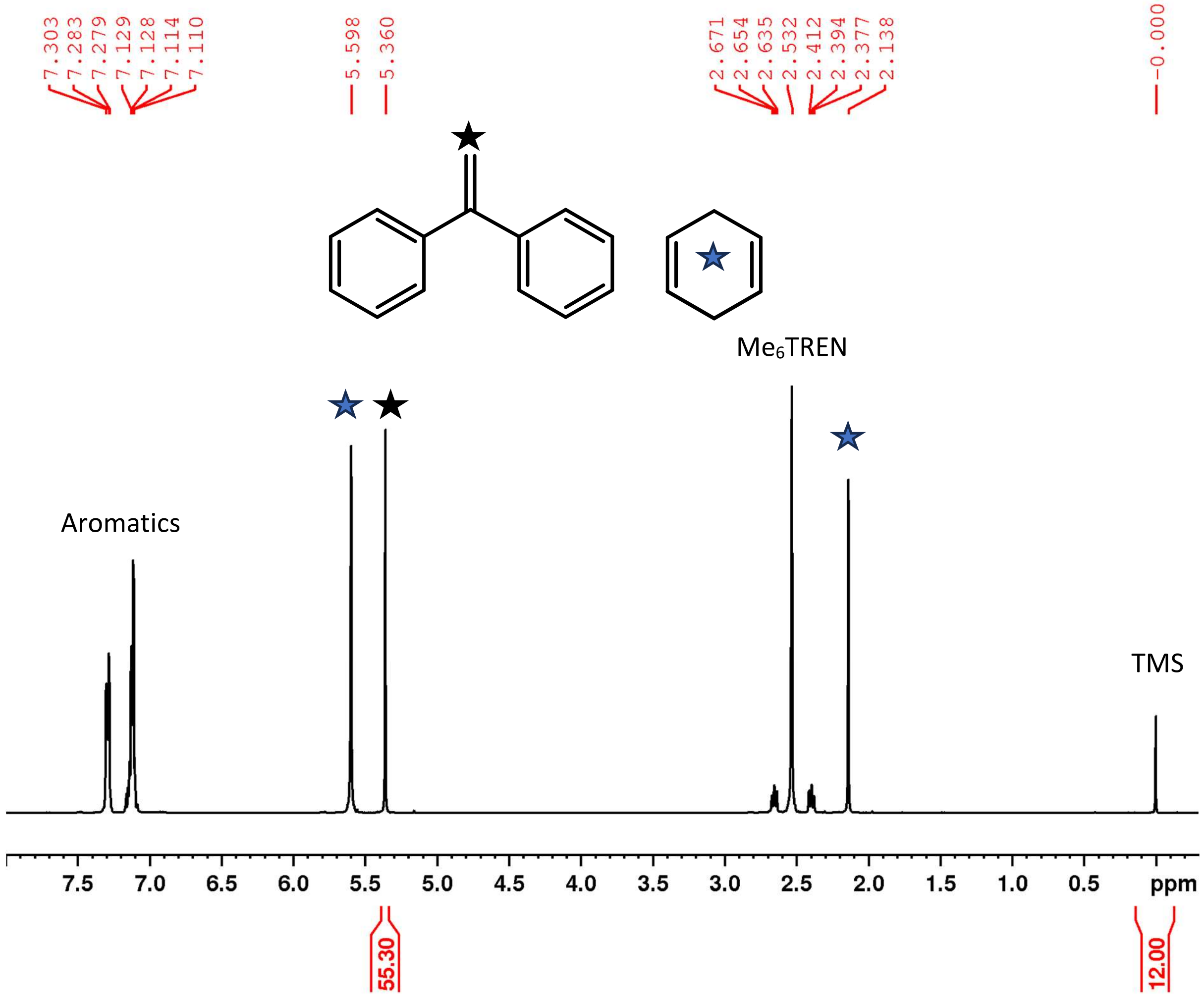

Supplementary Figure 25 – ^{1}H NMR spectrum of the completed transfer hydrogenation reaction between 1,1-diphenylethylene (0.3 mmol) and [NaH]·Me$_6$TREN (10 mol%) as catalyst using 1.5 equiv. of 1,4-cyclohexadiene in C_6D_6 showing no formation of the alkane product (1,1-diphenylethane) after heating for 24 hours at 70 °C. Tetramethylsilane standard was used (0.06 mmol) to calculate the percentage yield (entry 9, yield 0%).

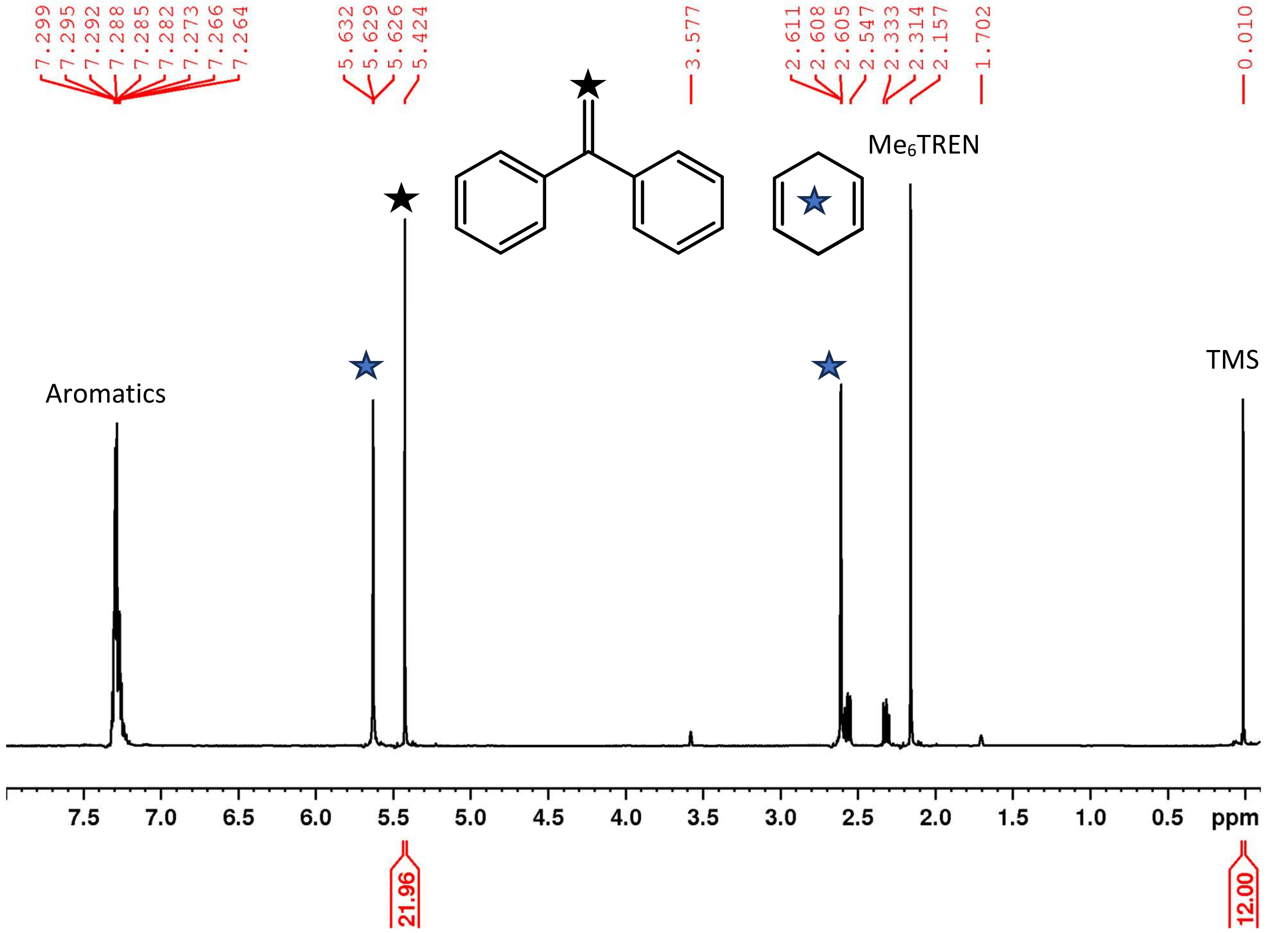

Supplementary Figure 26 – ^{1}H NMR spectrum of the completed transfer hydrogenation reaction between 1,1-diphenylethylene (0.3 mmol) and [Na(H)]·Me_6TREN (10 mol%) as catalyst using 1.5 equiv. of 1,4-cyclohexadiene in THF-D_8 showing no formation of the alkane product (1,1-diphenylethane) after heating for 24 hours at 70 °C. Tetramethylsilane standard was used (0.06 mmol) to calculate the percentage yield (entry 10, yield 0%).

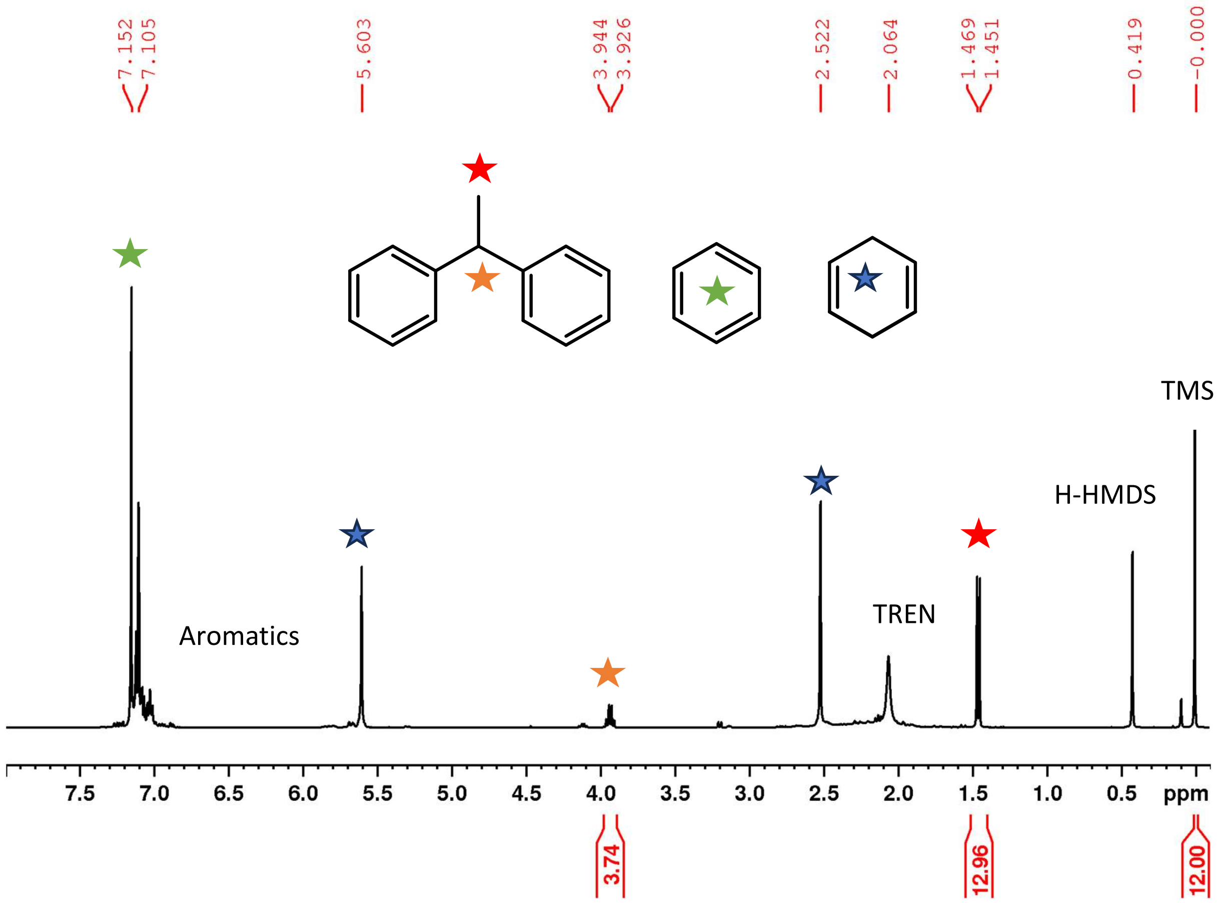

Supplementary Figure 27 – ^{1}H NMR spectrum of the completed transfer hydrogenation reaction between 1,1-diphenylethylene (0.3 mmol) and [Na(HMDS)]·Me_6TREN (10 mol%) as catalyst using 1.5 equiv. of 1,4-cyclohexadiene in C_6D_6 showing partial formation of the alkane product (1,1-diphenylethane) after heating for 5 hours at 70 °C. Tetramethylsilane standard was used (0.06 mmol) to calculate the percentage yield (entry 11, yield 79%).

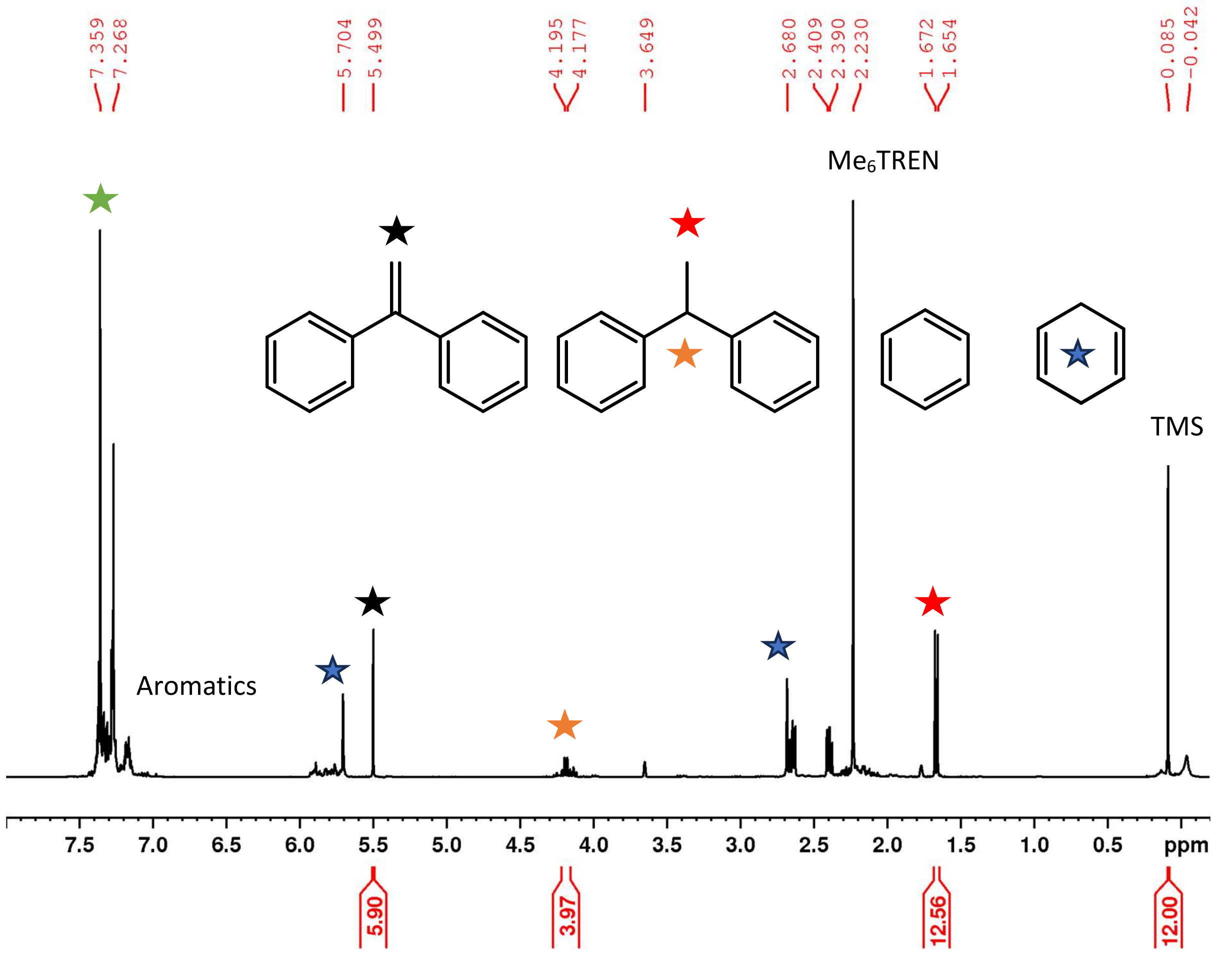

Supplementary Figure 28 – ^{1}H NMR spectrum of the completed transfer hydrogenation reaction between 1,1-diphenylethylene (0.3 mmol) and [Na(HMDS)]·Me$_6$TREN (10 mol%) as catalyst using 1.5 equiv. of 1,4-cyclohexadiene in THF-D$_8$ showing partial formation of the alkane product (1,1-diphenylethane) after heating for 24 hours at 70 °C. Tetramethylsilane standard was used (0.06 mmol) to calculate the percentage yield (entry 12, yield 80%).

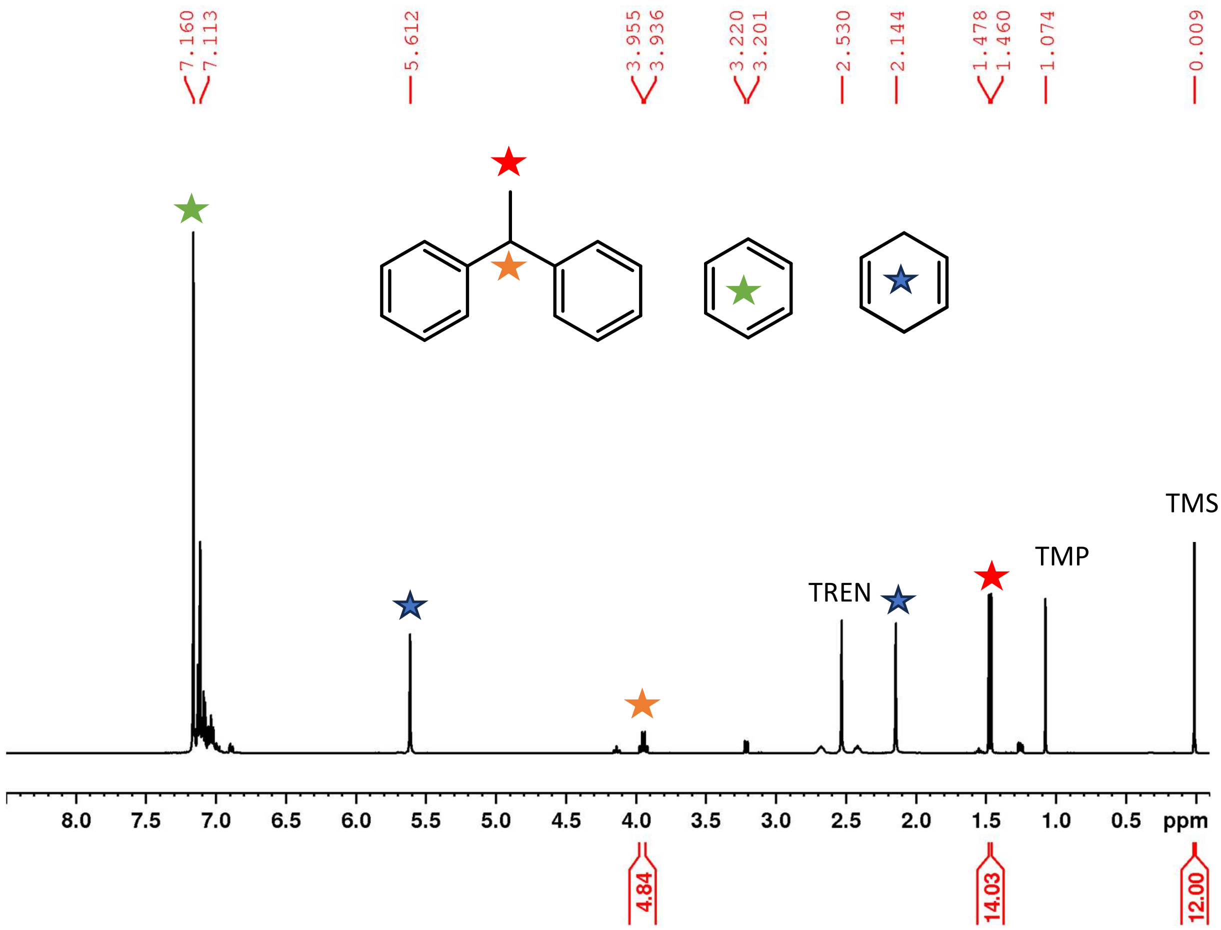

Supplementary Figure 29 – ^{1}H NMR spectrum of the completed transfer hydrogenation reaction between 1,1-diphenylethylene (0.3 mmol) and [Na(TMP)·Me$_6$TREN] (10 mol%) as catalyst using 1.5 equiv. of 1,4-cyclohexadiene in C_6D_6 showing formation of the alkane product (1,1-diphenylethane) after heating for 2.5 hours at 70 °C. Tetramethylsilane standard was used (0.06 mmol) to calculate the percentage yield (entry 13, yield 95%).

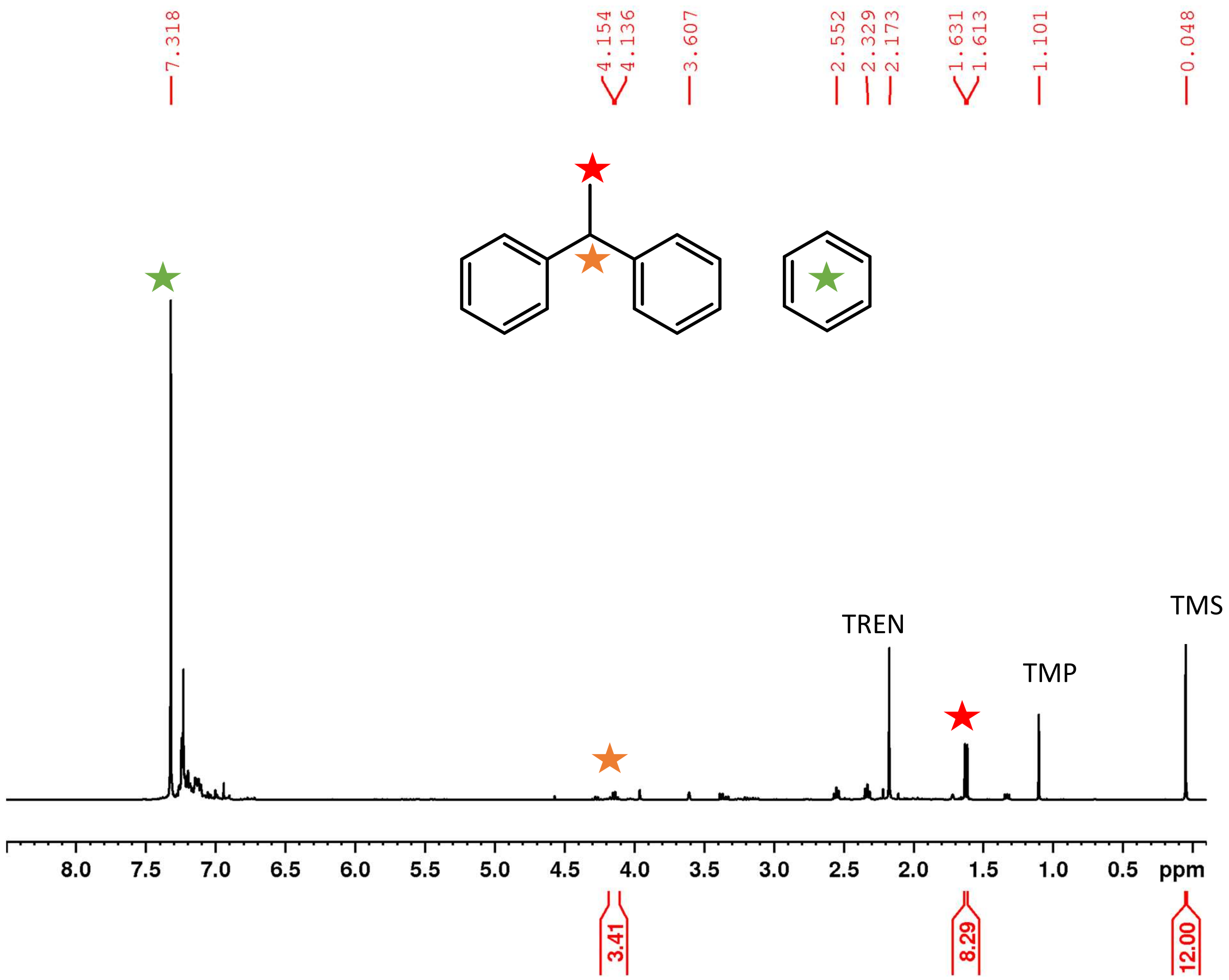

Supplementary Figure 30 – ^{1}H NMR spectrum of the completed transfer hydrogenation reaction between 1,1-diphenylethylene (0.3 mmol) and [Na(TMP)·Me_6TREN] (10 mol%) as catalyst using 1.5 equiv. of 1,4-cyclohexadiene in THF-D_8 showing partial formation of the alkane product (1,1-diphenylethane) after heating for 24 hours at 70 °C. Tetramethylsilane standard was used (0.06 mmol) to calculate the percentage yield (entry 14, yield 57%).

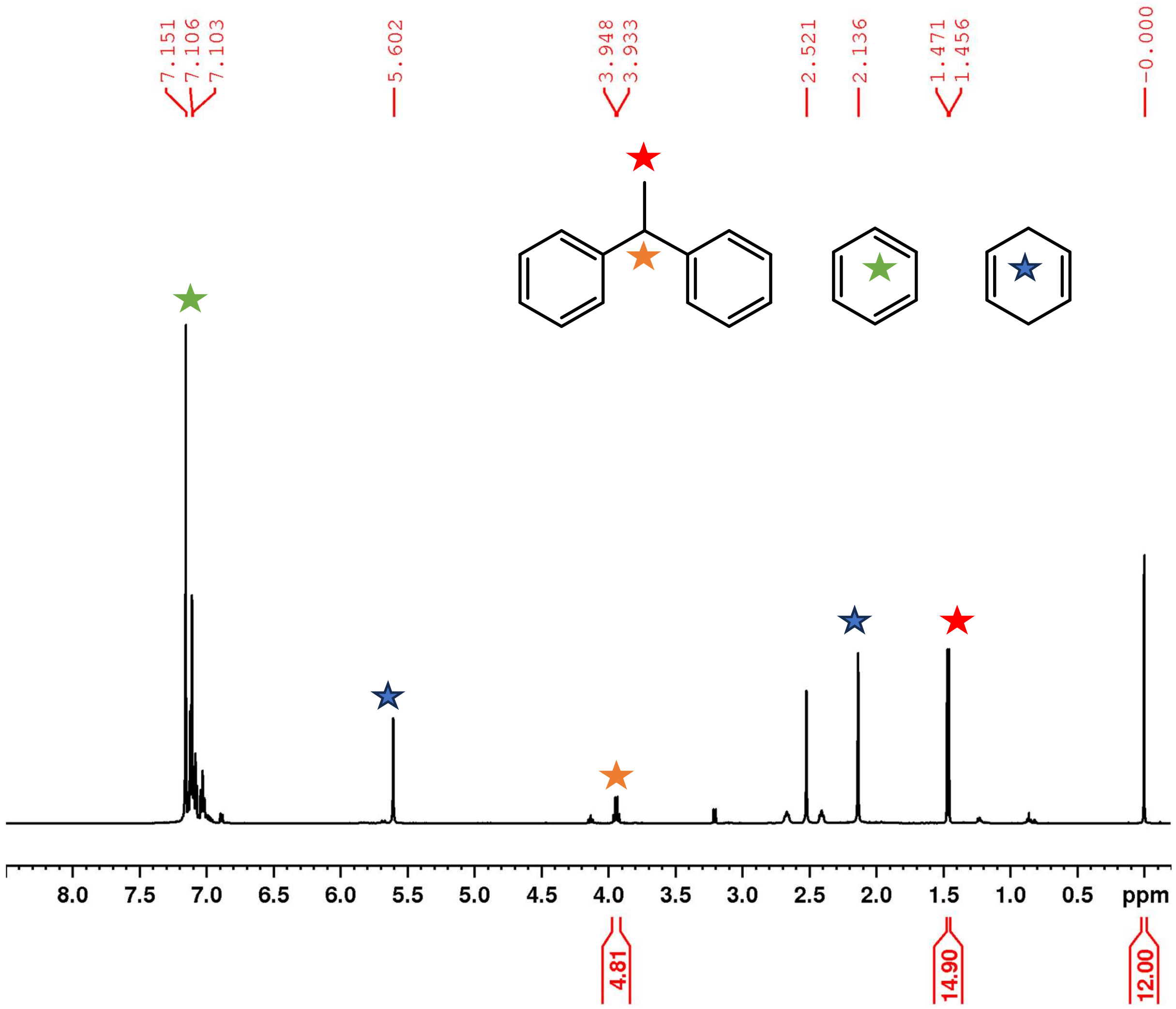

Supplementary Figure 31 - ^1H NMR spectrum of the completed transfer hydrogenation reaction between 1,1-diphenylethylene (0.3 mmol) and [BuNa]·Me_6TREN (10 mol%) as catalyst using 1.5 equiv. of 1,4-cyclohexadiene in C_6D_6 showing formation of the alkane product (1,1-diphenylethane) after heating for 3 hours at 70 °C. Tetramethylsilane standard was used (0.06 mmol) to calculate the percentage yield (entry 15, yield 96%).

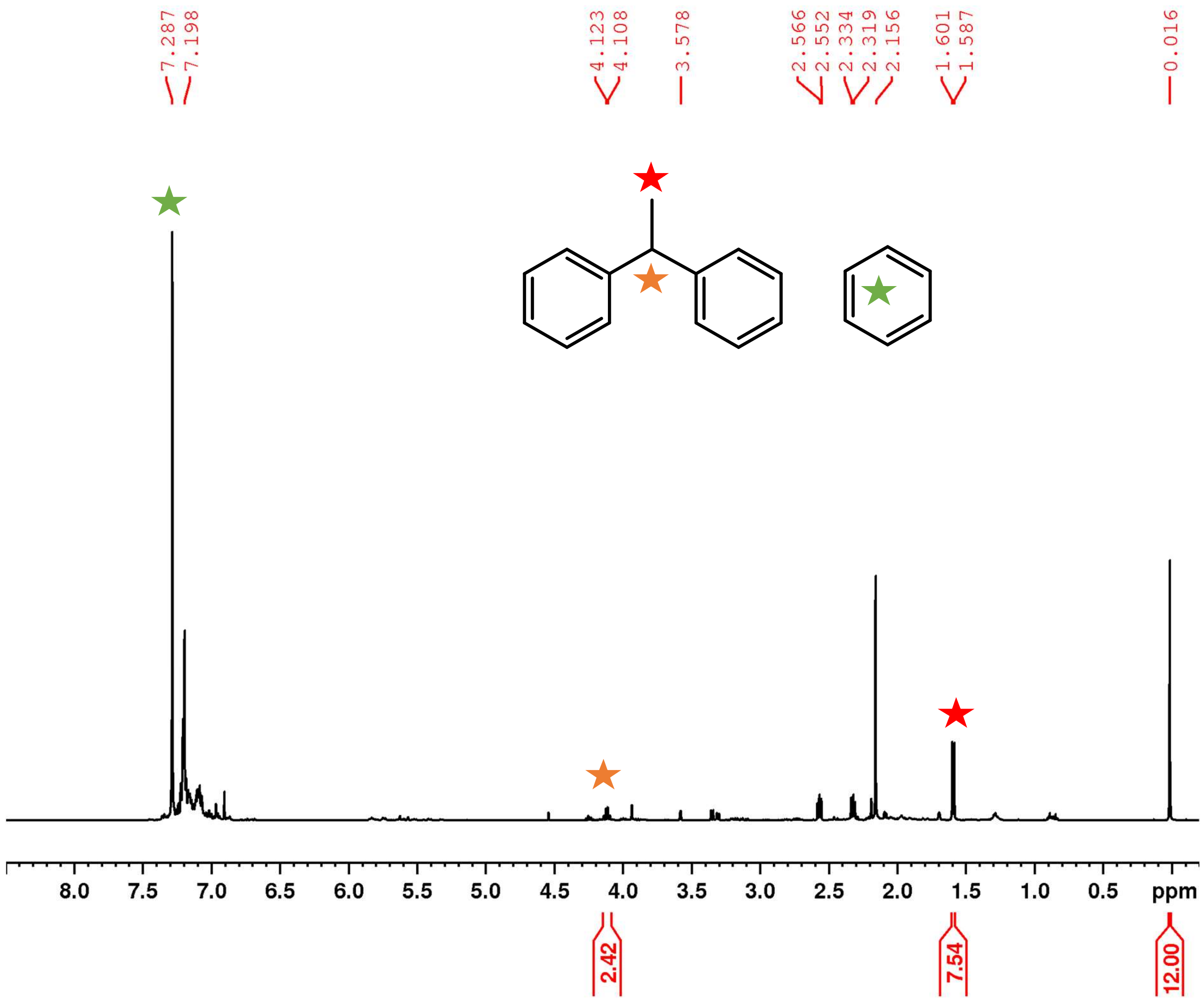

Supplementary Figure 32 - ^{1}H NMR spectrum of the completed transfer hydrogenation reaction between 1,1-diphenylethylene (0.3 mmol) and [BuNa]·Me_6TREN (10 mol%) as catalyst using 1.5 equiv. of 1,4-cyclohexadiene in THF-d_8 showing formation of the alkane product (1,1-diphenylethane) after heating for 3 hours at 70 °C. Tetramethylsilane standard was used (0.06 mmol) to calculate the percentage yield (entry 16, yield 50%).

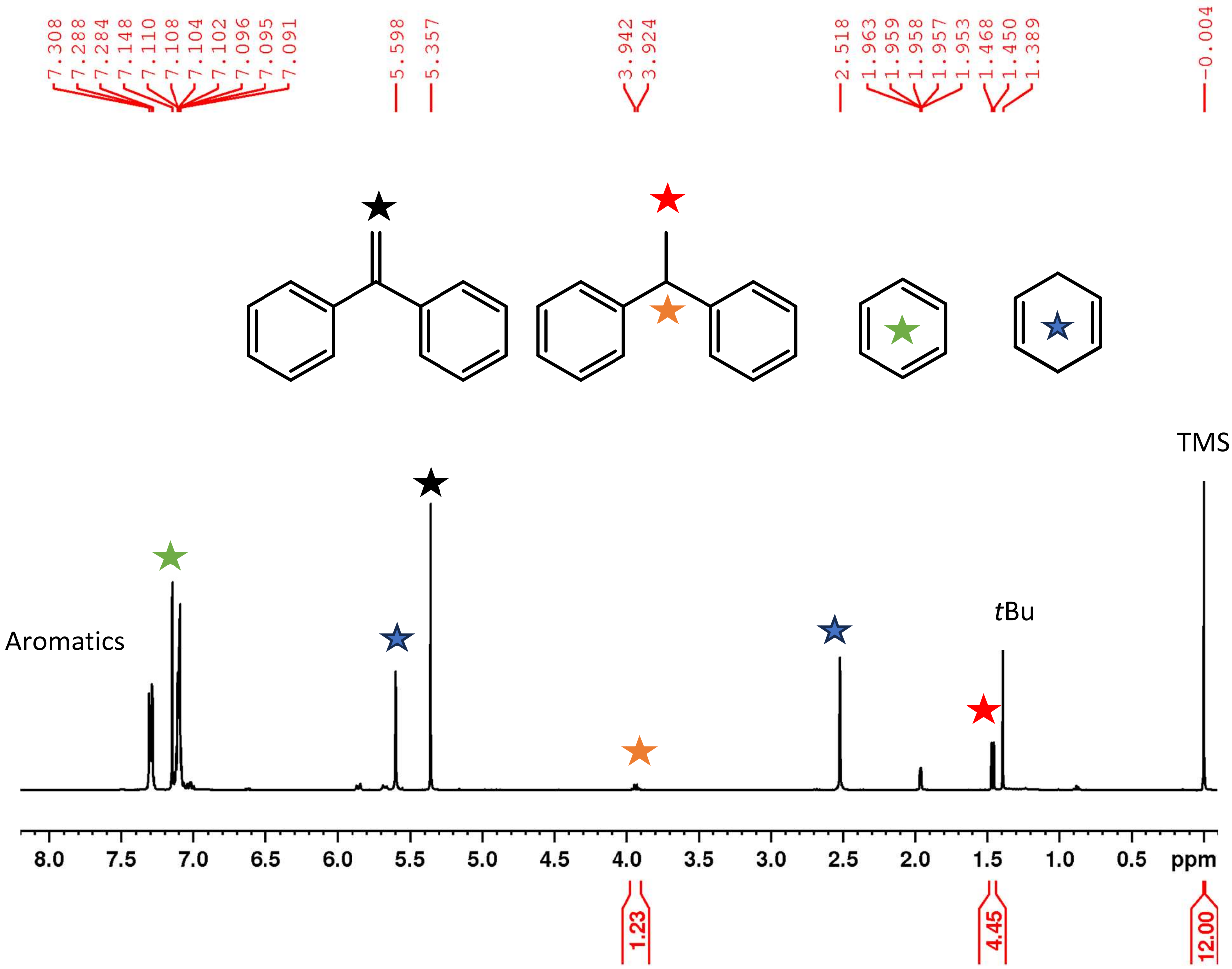

Supplementary Figure 33 – ^{1}H NMR spectrum of the completed transfer hydrogenation reaction between 1,1-diphenylethylene (0.3 mmol) and NaDHP (10 mol%) as catalyst using 1.5 equiv. of 1,4-cyclohexadiene in C_6D_6 showing formation of the alkane product (1,1-diphenylethane) after heating for 24 hours at 70 °C. Tetramethylsilane standard was used (0.06 mmol) to calculate the percentage yield (entry 17, yield 29%).

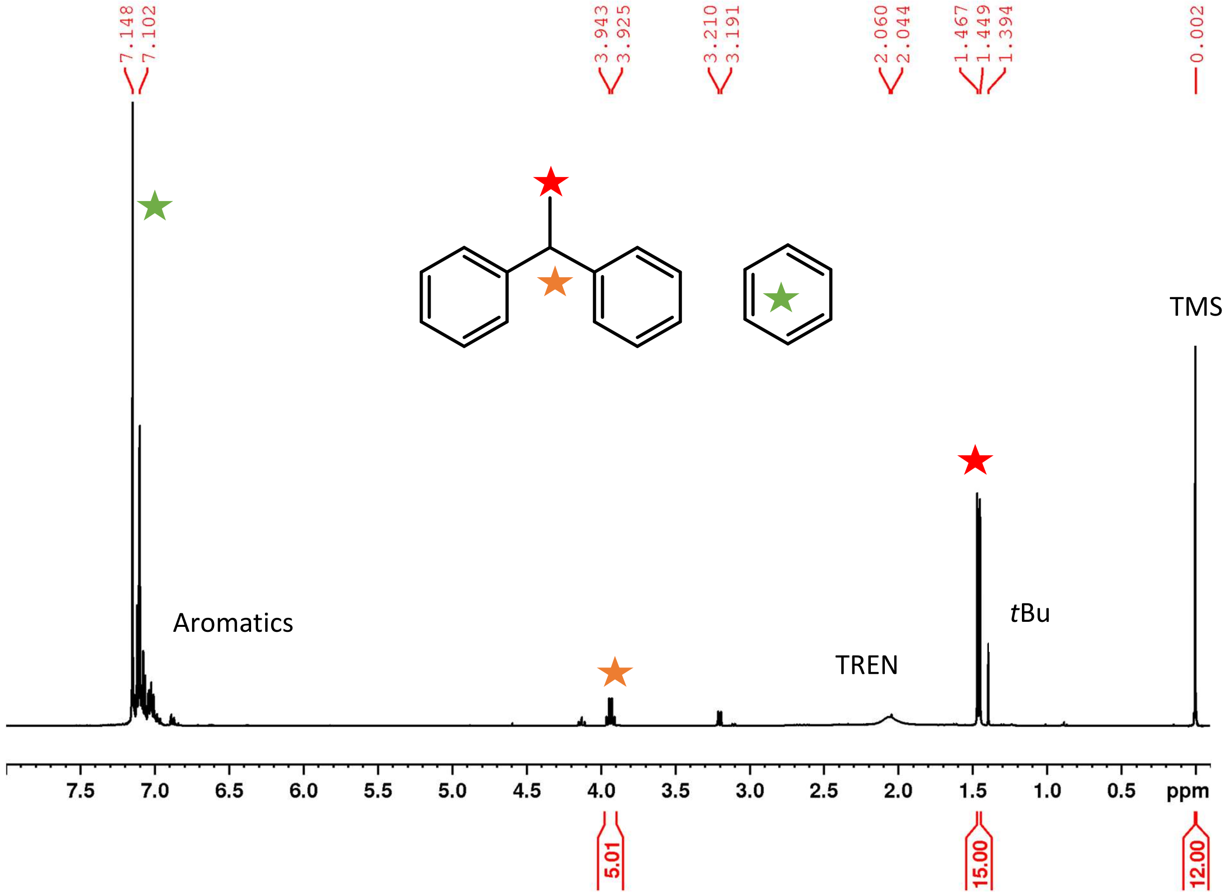

Supplementary Figure 34 – ^{1}H NMR spectrum of the completed transfer hydrogenation reaction between 1,1-diphenylethylene (0.3 mmol) and [Na(DHP)]·Me_6TREN (10 mol%) as catalyst using 1.5 equiv. of 1,4-cyclohexadiene in C_6D_6 showing formation of the alkane product (1,1-diphenylethane) after heating for 3 hours at 70 °C. Tetramethylsilane standard was used (0.06 mmol) to calculate the percentage yield (entry 18, yield 99%).

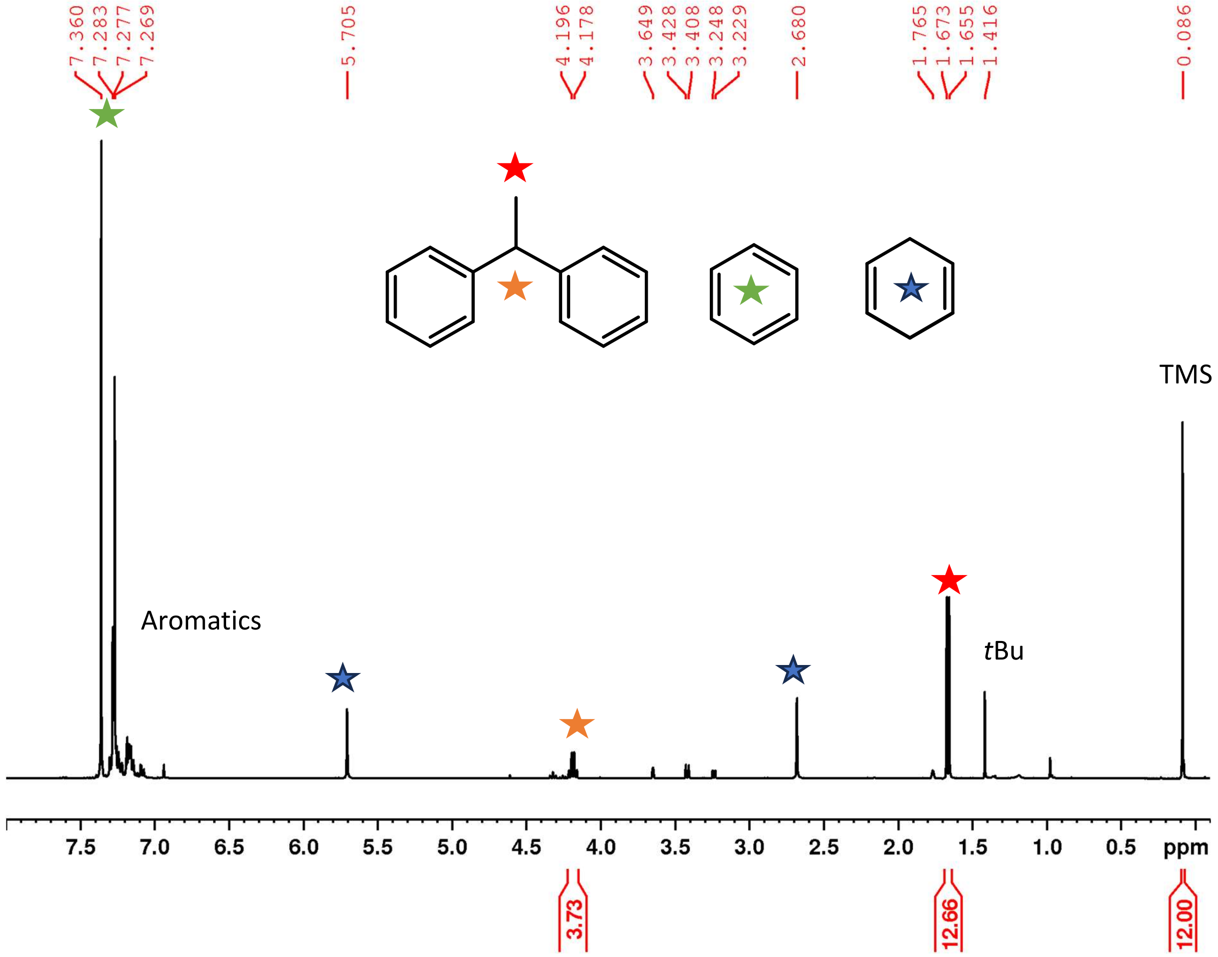

Supplementary Figure 35 – ¹H NMR spectrum of the completed transfer hydrogenation reaction between 1,1-diphenylethylene (0.3 mmol) and NaDHP (10 mol%) as catalyst using 1.5 equiv. of 1,4-cyclohexadiene in THF-D_8 showing formation of the alkane product (1,1-diphenylethane) after heating for 3 hours at 70 °C. Tetramethylsilane standard was used (0.06 mmol) to calculate the percentage yield (entry 19, yield 80%).

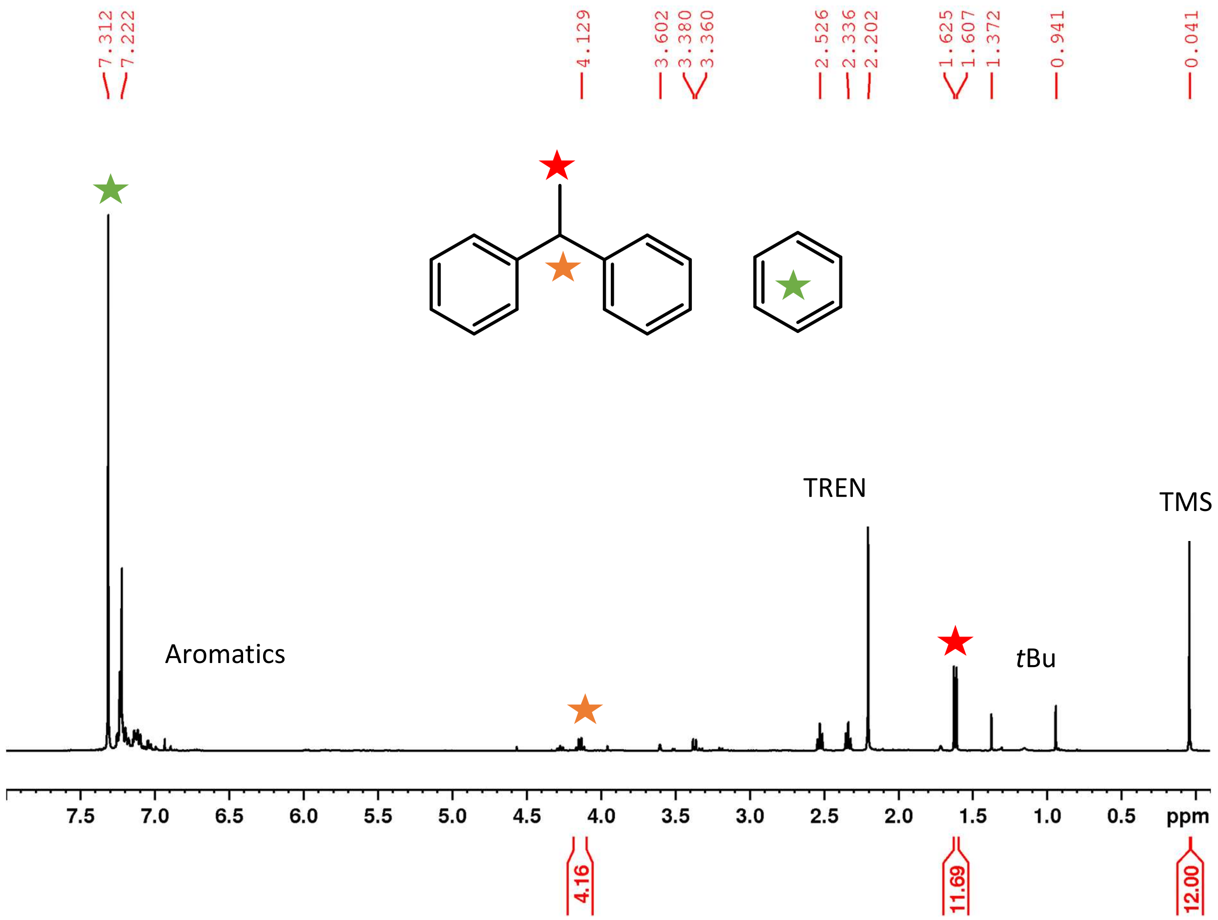

Supplementary Figure 36 – ^{1}H NMR spectrum of the completed transfer hydrogenation reaction between 1,1-diphenylethylene (0.3 mmol) and [Na(DHP)]·Me_6TREN (10 mol%) as catalyst using 1.5 equiv. of 1,4-cyclohexadiene in THF-D_8 showing formation of the alkane product (1,1-diphenylethane) after heating for 3 hours at 70 °C. Tetramethylsilane standard was used (0.06 mmol) to calculate the percentage yield (entry 20, yield 81%).

4. Stoichiometric experiments

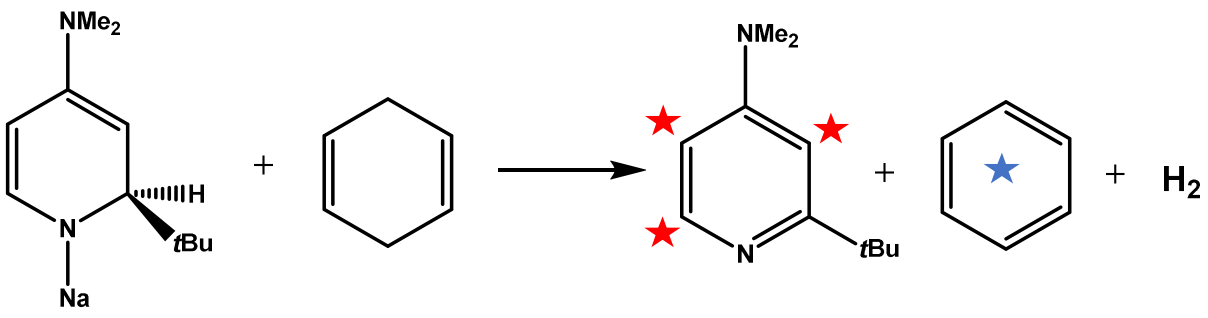

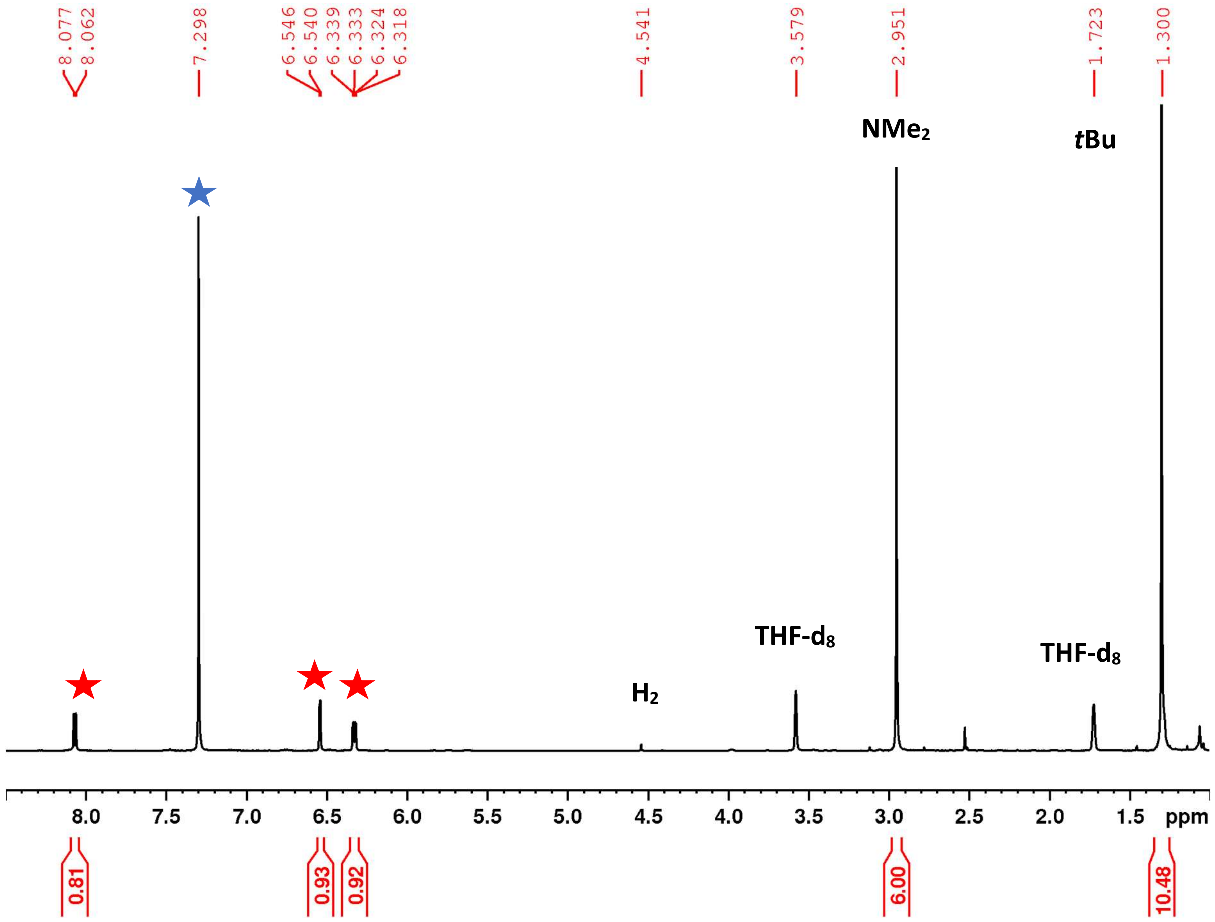

Supplementary Figure 37 – ^{1}H NMR spectrum of the completed reaction between **1·Me$_6$TREN** and 1,4–cyclohexadiene in THF-d_8 confirming the rearomatization of the substituted pyridine, formation of benzene and evolution of H_2.

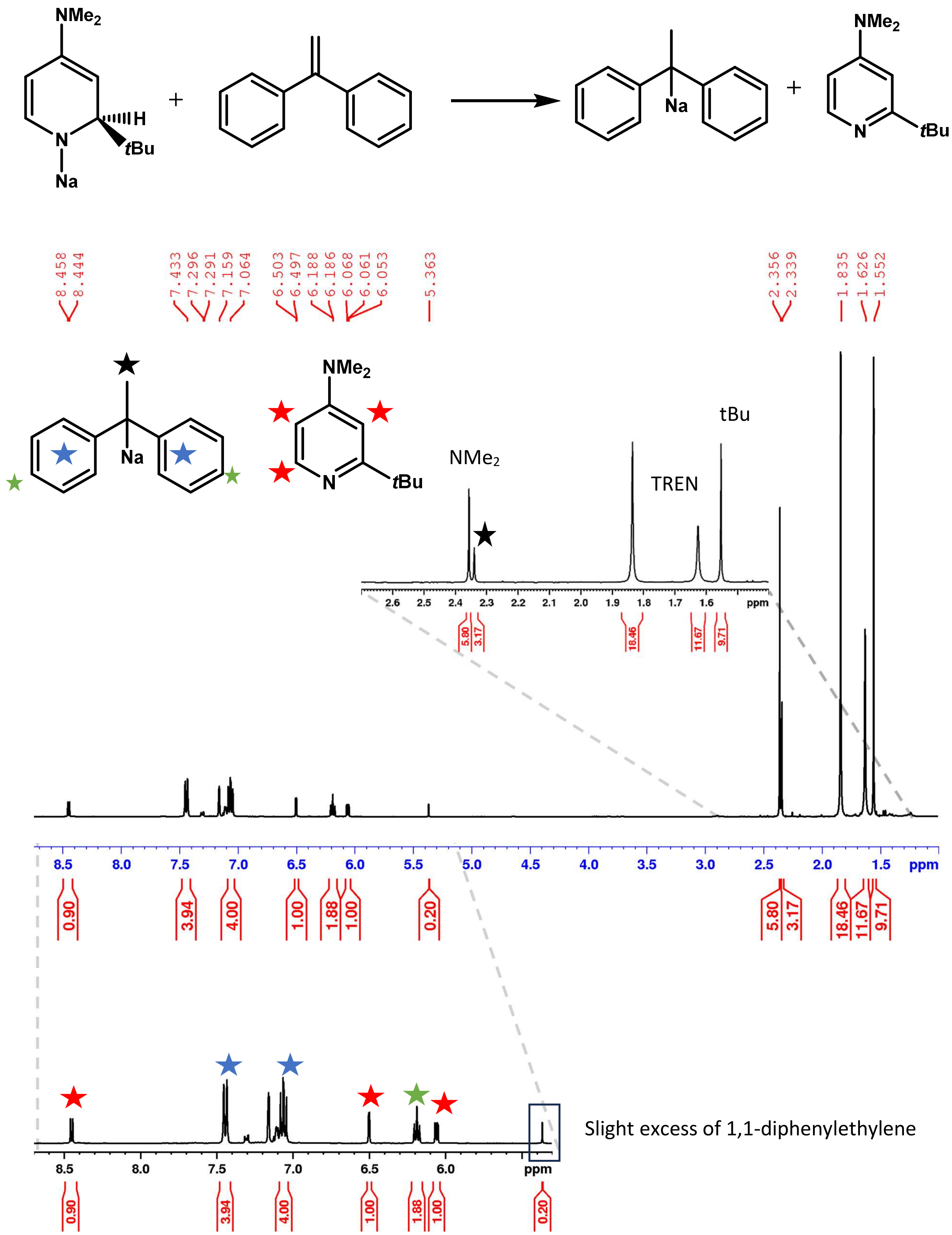

Supplementary Figure 38 – ^{1}H NMR spectrum of the completed reaction between **1·Me$_6$TREN** and 1,1-Diphenylethylene in C_6D_6 confirming the formation of 1,1-diphenylethylsodium and rearomatization of the substituted pyridine after 2 hours of heating at 70 °C.

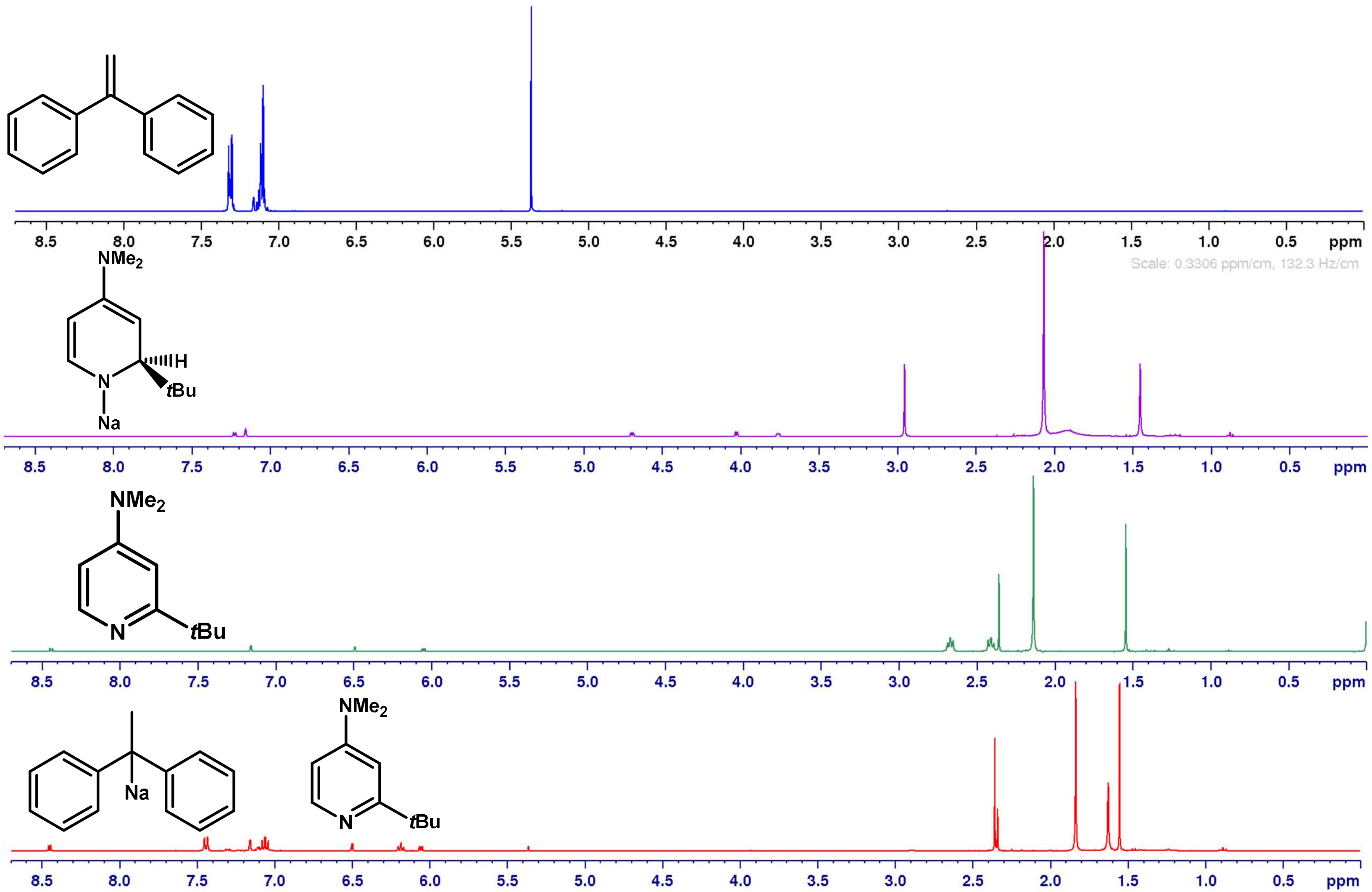

Supplementary Figure 39 – Stacked plot of ^{1}H NMR spectra showing reaction between **1·Me$_6$TREN** and 1,1-diphenylethylene (bottom) and standards of starting materials/products above.

5. Computational Details

All quantum chemical calculations were carried out using the Gaussian16 package[4]. The NBO analysis[5] was performed with version 6.0 which was implemented in the G09 D.01[6] version of the Gaussian program[7]. The molecular structure optimisations were performed using the M06-2X[8] functional along with the 6-311+G(d,p) basis set. Each stationary point was identified by a subsequent frequency calculation as minimum (Number of imaginary frequencies NIMAG: 0). All corresponding computed molecular structures are given in the cartesian coordinates supplied in section 1.4. For the NBO analysis a density was obtained using the M06-2X functional along with the 6-311+G(d,p) basis set was applied. AIM and CHELPG charges were calculated using multiwfn [9-11].

Three classes of compounds studied for both DHP and DH(DMAP) systems. 'Naked' anion, sodium anion and Me_6TREN coordinated sodium complex.

5.1 Structural Parameters

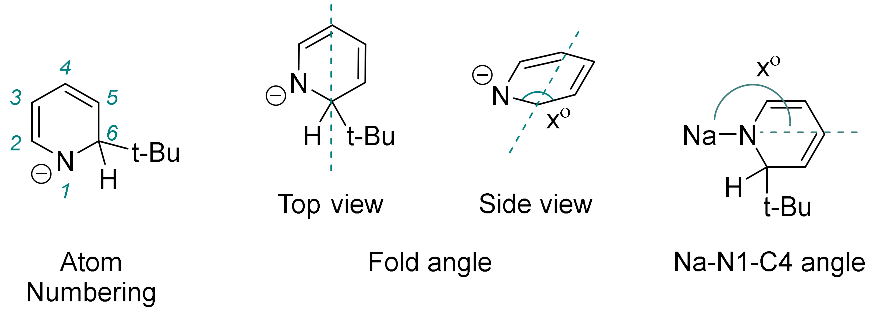

Supplementary Figure 40. Atom Labelling and bond angle definitions

Supplementary Table 10. Calculated Structural Parameters

	DHP	DHDMAP	NaDHP	NaDH (DMAP)	NaDHP·Me_6TREN	NaDH(DMAP)·Me_6TREN
**Bond Lengths / Å**						
AM-N1	n/a	n/a	2.340	2.333	2.239	2.244
N1-C2	1.309	1.308	1.324	1.323	1.333	1.331
C2-C3	1.414	1.412	1.407	1.403	1.381	1.378
C3-C4	1.429	1.432	1.427	1.437	1.434	1.443
C4-C5	1.360	1.365	1.369	1.384	1.351	1.358
C5-C6	1.509	1.510	1.514	1.516	1.513	1.512
C6-N1	1.465	1.465	1.478	1.477	1.471	1.469
**Bond Angles / °**						
Fold angle	142.5	142.9	143.7	144.9	161.1	166.3
Na-N1-C4 angle	n/a	n/a	62.2	63.8	156.5	139.5

Supplementary Table 11. Optimised Structures

DHP	NaDHP	NaDHP·Me_6TREN
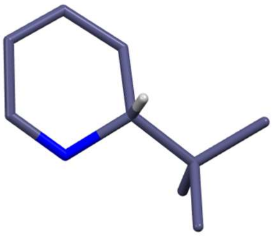	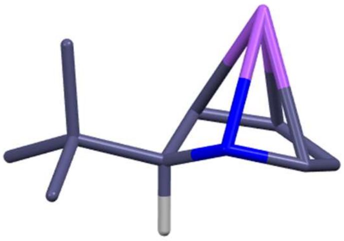	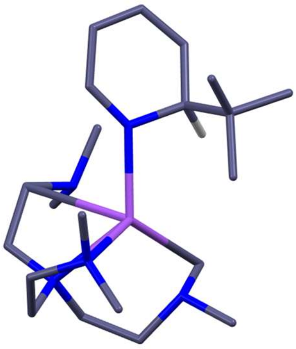
**DH(DMAP)**	**NaDH(DMAP)**	**NaDH(DMAP)·Me_6TREN**
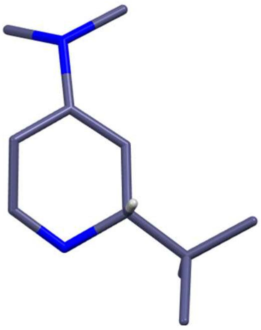	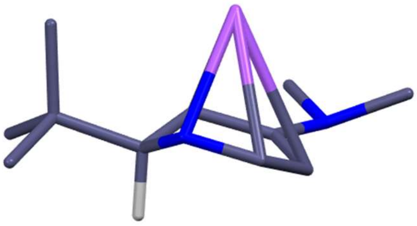	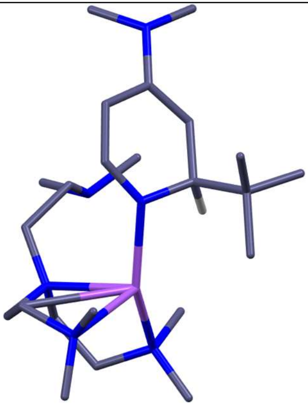

The naked anion and sodium anion display similar geometries, with the *sp*^{3} carbon distorting the plane of the ring to a similar degree. Inclusion of the Me_6TREN multidentate ligand results in a change in coordination of the Na atom from η^4 to η^1. Additionally, there is an increased planarity of the ring (fold angle 143.7° **NaDHP** vs 161.1° **NaDHP·Me_6TREN**) and the t-Bu group now perpendicular to the DHP. The same trend is observed with the DH(DMAP) derivatives.

On comparing DHP vs. DH(DMAP) structural parameters, there is no significant difference between the 'naked' anions and the sodium anions or within the bond lengths of the Me_6TREN derivatives. The most notable difference from the optimised structures is in the position of the sodium atom in the Me_6TREN derivates. In each case the sodium is η^1 bound directly to the nitrogen, however, in the DH(DMAP) system the sodium is tending more towards the ring as evidenced by the change in Na-N1-C4 bond angle (156.5° (**NaDHP·Me_6TREN**) vs 139.5° (**NaDH(DMAP)·Me_6TREN**) (Supplementary figure 41).

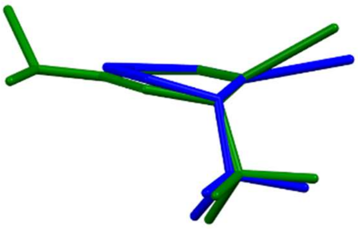

Supplementary Figure 41. Structural overlay NaDHP NaDHP·Me_6TREN (blue) vs. NaDH(DMAP)·Me_6TREN (green).

5.2 Frontier Orbitals

Supplementary Table 12. Frontier Orbital Energies

	DHP	DH (DMAP)	NaDHP	NaDH (DMAP)	NaDHP·Me_6TREN	NaDH(DMAP)·Me_6TREN	NaHMDS·Me_6TREN
**Energies (eV)**							
HOMO	-0.82	-0.97	-5.51	-5.40	-4.23	-4.37	-6.58
LUMO	3.16	2.96	-1.23	-1.14	-0.61	-0.57	-0.55
$\Delta_{H\text{-}L}$	3.98	3.93	4.28	4.26	3.62	3.80	6.03

In all cases the HOMO consists of the DHP/DH(DMAP) ring and hydride. Incorporation of sodium notably lowers the overall energies of the frontier orbitals (Table C3: HOMO -0.82 eV (DHP) vs -5.51 eV (NaDHP)), suggesting a more stable system is obtained. This matches that of the experimentally observed data, with NaDHP/NaDH(DMAP) being less reactive in contrast to when donor ligands are employed. Incorporation of Me_6TREN raises the overall energies of the frontier orbitals and narrows the HOMO-LUMO gaps, suggesting a more reactive system. Again in line with experimental observations, the decreased reactivity the NaHMDS also coincides with a large $\Delta_{H\text{-}L}$ suggesting a less reactive system.

5.3 Charge Distributions

Supplementary Table 13. Charge Distributions of selected atoms

	DHP	DH (DMAP)	NaDHP	NaDH (DMAP)	NaDHP·Me_6TREN	NaDH(DMAP)·Me_6TREN
**NPA Charges**						
Na	n/a	n/a	0.930	0.918	0.852	0.863
N1	-0.653	-0.653	-0.703	-0.713	-0.835	-0.838
C6	-0.064	-0.054	-0.073	-0.064	-0.043	-0.027
H	0.143	0.146	0.178	0.177	0.154	0.136
**AIM Charges**						
Na	n/a	n/a	0.882	0.875	0.864	0.866
N1	-1.194	-1.197	-1.204	-1.213	-1.266	-1.266
C6	0.359	0.367	0.340	0.341	0.365	0.371
H	-0.059	-0.057	-0.019	-0.018	-0.078	-0.101
**CHELPG Charges**						
Na	n/a	n/a	0.715	0.736	0.019	0.266
N1	-0.986	-1.013	-0.986	-1.068	-0.500	-0.616
C6	1.166	0.948	1.342	1.285	1.224	0.939
H	-0.327	-0.265	-0.205	-0.217	-0.297	-0.219

To assess the charge distribution around the central ring unit, and examine the influence of the NMe_2 group, three population analysis methods were used: (a) Natural Population analysis (NPA) (based on the wave function), (b) Atoms in Molecules (AIM) (based on electron density) and (c) Charges from electrostatic potentials using a grid-based method (CHELPG) (based on electrostatic potential). Assessment of NPA and AIM charges reveals little

difference between the DHP and DHDMAP systems. However, in the CHELPG electrostatic derived charges a general trend can be observed. For the DHP systems, the hydride is more negative whereas in the DH(DMAP) systems the Nitrogen is more negative.

5.4 Cartesian Coordinates:

Naked DHP anion

C	-2.013600	1.200800	0.052900
C	-2.803300	0.076100	-0.280000
C	-2.178900	-1.175000	0.015200
C	-0.825800	-1.241000	0.133800
C	-0.086200	-0.008600	-0.326600
N	-0.713800	1.203900	0.205600
H	-2.796800	-2.042800	0.242700
H	-2.525300	2.135200	0.306800
H	-3.881900	0.166600	-0.330700
H	-0.318700	-2.120000	0.517200
C	1.431700	-0.003500	-0.026400
C	2.099700	-1.216900	-0.683000
H	3.189500	-1.160800	-0.576000
H	1.763500	-2.155000	-0.235300
H	1.862100	-1.251100	-1.751700
C	2.046300	1.272000	-0.613500
H	1.565000	2.147500	-0.175500
H	3.125200	1.308600	-0.419600
H	1.889600	1.306200	-1.697200
C	1.679800	-0.024600	1.484300
H	2.754300	0.020600	1.701800
H	1.179300	0.830300	1.944000
H	1.270400	-0.932900	1.935000
H	-0.178100	0.043300	-1.441000

Naked DH(DMAP) anion

C	-0.245400	2.240800	-0.041300
C	-1.403400	1.614600	-0.552400
C	-1.463100	0.200300	-0.338900
C	-0.299500	-0.475700	-0.112500
C	0.960200	0.313100	-0.377800
N	0.893100	1.651000	0.216200
H	-0.309400	3.298800	0.234100
H	-2.297800	2.189300	-0.757200
H	-0.272000	-1.500000	0.236000
C	2.264700	-0.400200	0.052900
C	2.390200	-1.748300	-0.666100
H	3.368000	-2.201400	-0.464500
H	1.619200	-2.452600	-0.345200
H	2.292200	-1.613700	-1.748700
C	3.457700	0.477500	-0.342300
H	3.373000	1.452500	0.139400
H	4.402100	0.002600	-0.050500
H	3.471800	0.635500	-1.426300

C	2.286300	-0.612100	1.569200
H	3.229900	-1.076500	1.882100
H	2.172200	0.351700	2.069900
H	1.459900	-1.253500	1.887400
N	-2.752400	-0.428600	-0.293200
C	-3.648900	0.118400	0.710600
H	-4.661100	-0.268000	0.549500
H	-3.329700	-0.155500	1.732600
H	-3.669900	1.203600	0.644500
C	-2.740100	-1.872000	-0.223200
H	-2.380900	-2.252400	0.750300
H	-3.757700	-2.246200	-0.376000
H	-2.092200	-2.270700	-1.004800
H	1.044400	0.461000	-1.483000

NaDHP

C	-1.815500	0.841500	-0.972500
C	-2.540700	-0.354700	-0.825000
C	-1.941100	-1.332800	0.023300
C	-0.611800	-1.238800	0.338300
C	0.179300	-0.272600	-0.518100
N	-0.550700	1.008700	-0.616900
H	-2.561700	-2.105500	0.471200
H	-2.351800	1.748600	-1.263900
H	-3.577300	-0.418200	-1.124700
H	-0.137000	-1.927400	1.027700
C	1.637900	-0.032100	-0.071000
C	2.398600	-1.362900	-0.061500
H	3.463200	-1.189700	0.120100
H	2.035700	-2.041700	0.713900
H	2.298800	-1.868800	-1.026300
C	2.308400	0.915900	-1.070900
H	1.773400	1.865100	-1.115500
H	3.348600	1.100900	-0.786400
H	2.303300	0.477200	-2.073100
C	1.694300	0.598800	1.324900
H	2.731500	0.754600	1.635000
H	1.201700	1.576700	1.318900
H	1.224600	-0.048100	2.075500
H	0.230500	-0.692700	-1.544500
Na	-1.453100	0.830300	1.535100

NaDH(DMAP)

C	0.239100	-2.084500	-0.634500
C	1.415600	-1.368200	-0.900300
C	1.455700	0.000700	-0.464200
C	0.260200	0.593500	-0.097200
C	-0.985900	-0.119500	-0.584400
N	-0.916800	-1.559200	-0.262100
H	0.289400	-3.176600	-0.632800
H	2.293600	-1.884200	-1.256700

H	0.208800	1.610600	0.263400
C	-2.318000	0.483400	-0.080700
C	-2.418300	1.952600	-0.507100
H	-3.420100	2.339700	-0.299200
H	-1.702700	2.587800	0.020300
H	-2.233300	2.053800	-1.580500
C	-3.475900	-0.295100	-0.714900
H	-3.413800	-1.351800	-0.453400
H	-4.437300	0.105300	-0.379100
H	-3.436000	-0.213700	-1.805200
C	-2.436700	0.387600	1.444900
H	-3.379000	0.826100	1.785700
H	-2.432000	-0.661400	1.760400
H	-1.626800	0.933000	1.944500
N	2.687900	0.666800	-0.377700
C	3.821400	-0.079800	0.137000
H	4.734800	0.493800	-0.028500
H	3.728800	-0.268500	1.221900
H	3.926700	-1.035300	-0.368000
C	2.664200	2.032600	0.100400
H	2.386800	2.106000	1.165700
H	3.656500	2.467800	-0.025000
H	1.954700	2.619800	-0.482300
H	-1.017600	-0.047400	-1.692100
Na	0.199300	-1.116200	1.738600

NaDHP·Me_6TREN

N	-1.626100	0.268400	-0.679700
N	1.019200	-2.163300	-1.530200
N	2.583900	0.274400	-0.635000
N	1.688400	-0.789500	2.069200
N	1.091700	2.774100	0.219900
C	-2.119900	0.750300	-1.820000
C	-3.275600	1.488900	-1.982500
C	-3.914800	1.916000	-0.771500
C	-3.522100	1.436100	0.428400
C	-2.491700	0.331200	0.508300
C	-3.106300	-1.075300	0.840600
C	-3.953300	-0.969800	2.111000
H	-4.820800	-0.326900	1.948100
H	-3.369300	-0.549400	2.938400
H	-4.314000	-1.956200	2.418400
C	-1.963900	-2.065300	1.091700
H	-2.347400	-3.045000	1.392900
H	-1.312100	-1.702600	1.898100
H	-1.366800	-2.202400	0.185500
C	-3.967800	-1.569000	-0.321700
H	-4.427000	-2.534200	-0.081700
H	-3.362300	-1.682300	-1.224200
H	-4.760600	-0.849000	-0.538800
C	-0.133500	-2.453900	-2.385600

H	-0.587900	-1.521700	-2.722300
H	-0.887100	-2.999300	-1.816100
H	0.154300	-3.063000	-3.257400
C	1.514500	-3.396400	-0.928600
H	1.866200	-4.113200	-1.688900
H	0.711000	-3.864400	-0.356400
H	2.336600	-3.190600	-0.240900
C	2.055800	-1.480000	-2.307800
H	1.559900	-0.706600	-2.903300
H	2.533900	-2.176600	-3.018100
C	3.128000	-0.826300	-1.440300
H	3.566500	-1.573700	-0.778700
H	3.947400	-0.473300	-2.085000
C	3.321500	0.460200	0.620100
H	3.010400	1.413600	1.043900
H	4.407000	0.525600	0.432900
C	3.084700	-0.639200	1.652900
H	3.415500	-1.606200	1.264100
H	3.723400	-0.421400	2.527100
C	1.552200	-1.996300	2.881200
H	1.829900	-2.873600	2.293200
H	0.516500	-2.112500	3.201500
H	2.190900	-1.957800	3.778300
C	1.224400	0.377100	2.826800
H	1.854300	0.558400	3.712900
H	0.199900	0.200000	3.161700
H	1.222600	1.267100	2.192400
C	2.531000	1.525600	-1.414500
H	2.527300	1.271300	-2.477600
H	3.448300	2.110300	-1.248300
C	1.276700	2.361300	-1.167300
H	1.308100	3.238400	-1.840100
H	0.401100	1.769100	-1.450700
C	-0.272600	3.265600	0.423300
H	-0.454700	4.196000	-0.139000
H	-0.430200	3.471300	1.485100
H	-0.996100	2.517500	0.096600
C	2.040700	3.809700	0.602800
H	3.071600	3.477400	0.463800
H	1.904500	4.057900	1.658200
H	1.898900	4.730900	0.012900
H	-1.841900	0.536300	1.388100
H	-3.923900	1.843300	1.350300
H	-1.487900	0.589000	-2.700100
H	-3.568300	1.863900	-2.952400
H	-4.677000	2.689700	-0.814800
Na	0.361900	-0.443300	0.063500

NaDH(DMAP)·Me_6TREN

N	-0.601100	-1.267500	-0.612200
N	-4.516800	0.421400	-0.828200

N	2.867300	-1.552700	-1.505200
N	1.981000	1.353900	-0.815300
N	2.664200	0.407100	1.936200
N	-0.337000	3.028300	0.601500
C	-1.249600	-1.015700	-1.746400
C	-2.536500	-0.547000	-1.898600
C	-3.254100	-0.205900	-0.694100
C	-2.698300	-0.486300	0.513000
C	-1.394000	-1.243900	0.624000
C	-1.550900	-2.694800	1.203400
C	-2.309500	-2.637300	2.531200
H	-3.340000	-2.308700	2.379400
H	-1.828300	-1.941000	3.228800
H	-2.334900	-3.623700	3.003700
C	-0.157200	-3.280200	1.452200
H	-0.222100	-4.279800	1.892000
H	0.406400	-2.651400	2.157500
H	0.398600	-3.356000	0.514000
C	-2.309800	-3.569700	0.205900
H	-2.461300	-4.576600	0.608300
H	-1.754600	-3.646700	-0.731800
H	-3.286500	-3.130700	-0.015100
C	-4.545400	1.589900	-1.693600
H	-4.070000	2.462600	-1.213000
H	-5.582600	1.847000	-1.921600
H	-4.023400	1.391400	-2.626300
C	-5.252900	0.657900	0.394000
H	-5.336700	-0.269500	0.962200
H	-6.256900	1.003200	0.138800
H	-4.780800	1.420800	1.037100
C	2.181300	-2.770800	-1.949800
H	1.100800	-2.614100	-1.909600
H	2.434200	-3.594700	-1.279100
H	2.482000	-3.052800	-2.971200
C	4.301800	-1.802100	-1.413100
H	4.730200	-2.078400	-2.390400
H	4.484400	-2.620800	-0.714900
H	4.830500	-0.922500	-1.043500
C	2.569100	-0.453800	-2.433600
H	1.497100	-0.490000	-2.647100
H	3.100900	-0.605900	-3.388000
C	2.912700	0.921900	-1.868900
H	3.926100	0.909400	-1.463700
H	2.922900	1.655100	-2.690300
C	2.657500	2.197300	0.177700
H	1.896900	2.773000	0.702900
H	3.337000	2.916800	-0.309000
C	3.465700	1.377200	1.181800
H	4.250800	0.814600	0.665900
H	3.973300	2.073800	1.871900
C	3.540800	-0.426400	2.753800

H	4.252700	-0.954400	2.115200
H	2.947400	-1.165400	3.295600
H	4.106100	0.168800	3.488700
C	1.669900	1.072200	2.781800
H	2.148200	1.757500	3.501000
H	1.112800	0.317900	3.342000
H	0.965500	1.641600	2.171900
C	0.817300	2.021200	-1.425100
H	0.507300	1.414500	-2.280400
H	1.116500	3.004000	-1.827200
C	-0.424600	2.123000	-0.545900
H	-1.268500	2.412000	-1.198700
H	-0.664700	1.122500	-0.181300
C	-1.483000	2.814600	1.475300
H	-2.438300	3.019200	0.962700
H	-1.406300	3.467200	2.348300
H	-1.508000	1.773700	1.804800
C	-0.272700	4.420900	0.188600
H	0.631200	4.610000	-0.394200
H	-0.242600	5.063800	1.070700
H	-1.144700	4.709700	-0.423400
H	-0.789800	-0.730700	1.416200
H	-3.163600	-0.160200	1.434200
H	-0.665000	-1.180900	-2.658200
H	-2.975000	-0.438400	-2.879000
Na	1.410600	-0.769500	0.247800

6. Supplementary References

[1] W. L. F. Armarego, C. Chai, in *Purification of Laboratory Chemicals (Seventh Edition)* (Eds.: W. L. F. Armarego, C. Chai), Butterworth-Heinemann, Boston, **2013**, pp. 1-70.

[2] Ojeda-Amador, A.I., Martinez-Martinez, A.J., Kennedy, A.R., Armstrong, D.R. & O'Hara, C.T. Monodentate coordination of the normally chelating chiral diamine (*R*,*R*)-TMCDA. *Chem. Commun.*, **53**, 324-327 (2017).

[3] Bachmann, S., Gernert, B. & Stalke, D. Solution structures of alkali metal cyclopentadienides in THF estimated by ECC-DOSY NMR-spectroscopy (incl. software). *Chem. Commun.*, **52**, 12861-12864 (2016).

[4] Gaussian 16 Revision A.03, Frisch, M. J., *et al*, Gaussian, Inc., Wallingford CT, (2016).

[5] Reed, A.E., Curtiss, L.A. & Weinhold, F. Intermolecular Interactions from a Natural Bond Orbital, Donor-Acceptor Viewpoint. *Chem. Rev.*, **88**, 899-926 (1988).

[6] Gaussian 09 Revision D.01, Frisch, M. J., *et al.,* Gaussian, Inc., Wallingford CT (2013).

[7] Glendening, E.D., Landis, C.R. & Weinhold, F. NBO 6.0: natural bond orbital analysis program *J. Comput. Chem.* **34**, 1429-1437 (2013).

[8] Zhao, Y. & Truhlar, D.G. The M06 suite of density functionals for main group thermochemistry, thermochemical kinetics, noncovalent interactions, excited states, and transition elements:

two new functionals and systematic testing of four M06-class functionals and 12 other functionals. *Theor. Chem. Acc.*, **120**, 215-241 (2008).

[9] Lu, T. & Chen, F. Multiwfn: A multifunctional wavefunction analyzer. *J. Comput. Chem.,* **33**, 580-592 (2012).

[10] Zhang, J. & Lu, T. Efficient evaluation of electrostatic potential with computerized optimized code. *Phys. Chem. Chem. Phys.*, **23**, 20323-20328 (2021).

[11] Lu, T. & Chen, F.-W. Comparison of Computational Methods for Atomic Charges, *Acta Phys. -Chim. Sin.*, **28**, 1-18 (2012).
