# Supplementary material for: Synthesis, characterisation, and catalytic application of a soluble molecular carrier of sodium hydride activated by a substituted 4-(dimethylamino)pyridine
Source: Commun Chem. 2024 Apr 27;7:94. doi: 10.1038/s42004-024-01184-5 (PMC11055874; doi:10.1038/s42004-024-01184-5)
Supplement: Supplementary file 2 — Description of Additional Supplementary Files [file 42004_2024_1184_MOESM2_ESM.pdf]

Description of Additional Supplementary Files

File name: Supplementary Data 1

Description: CIF file
